# Calcineurin Signaling and Membrane Lipid Homeostasis Regulates Iron Mediated MultiDrug Resistance Mechanisms in *Candida albicans*

**DOI:** 10.1371/journal.pone.0018684

**Published:** 2011-04-12

**Authors:** Saif Hameed, Sanjiveeni Dhamgaye, Ashutosh Singh, Shyamal K. Goswami, Rajendra Prasad

**Affiliations:** School of Life Sciences, Jawaharlal Nehru University, New Delhi, India; University of Minnesota, United States of America

## Abstract

We previously demonstrated that iron deprivation enhances drug susceptibility of *Candida albicans* by increasing membrane fluidity which correlated with the lower expression of *ERG11* transcript and ergosterol levels. The iron restriction dependent membrane perturbations led to an increase in passive diffusion and drug susceptibility. The mechanisms underlying iron homeostasis and multidrug resistance (MDR), however, are not yet resolved. To evaluate the potential mechanisms, we used whole genome transcriptome and electrospray ionization tandem mass spectrometry (ESI-MS/MS) based lipidome analyses of iron deprived *Candida* cells to examine the new cellular circuitry of the MDR of this pathogen. Our transcriptome data revealed a link between calcineurin signaling and iron homeostasis. Among the several categories of iron deprivation responsive genes, the down regulation of calcineurin signaling genes including *HSP90, CMP1* and *CRZ1* was noteworthy. Interestingly, iron deprived *Candida* cells as well as iron acquisition defective mutants phenocopied molecular chaperone *HSP90* and calcineurin mutants and thus were sensitive to alkaline pH, salinity and membrane perturbations. In contrast, sensitivity to above stresses did not change in iron deprived DSY2146 strain with a hyperactive allele of calcineurin. Although, iron deprivation phenocopied compromised *HSP90* and calcineurin, it was independent of protein kinase C signaling cascade. Notably, the phenotypes associated with iron deprivation in genetically impaired calcineurin and *HSP90* could be reversed with iron supplementation. The observed down regulation of ergosterol (*ERG1*, *ERG2, ERG11* and *ERG25*) and sphingolipid biosynthesis (*AUR1* and *SCS7*) genes followed by lipidome analysis confirmed that iron deprivation not only disrupted ergosterol biosynthesis, but it also affected sphingolipid homeostasis in *Candida* cells. These lipid compositional changes suggested extensive remodeling of the membranes in iron deprived *Candida* cells. Taken together, our data provide the first novel insight into the intricate relationship between cellular iron, calcineurin signaling, membrane lipid homeostasis and drug susceptibility of *Candida* cells.

## Introduction

Iron is an indispensable element required both by the host as well as by the microbial community residing within the host [1]. Since iron in a human host is not freely available, therefore pathogenic organisms needs to exploit host iron reservoirs for its survival. This competition between the pathogen and host for iron represents a critical virulence trait of many infectious diseases [2]. In pathogenic *Candida albicans*, iron deprivation represents one of the crucial environmental stress conditions it encounters during infection process. Many studies have already established roles for iron in epithelial invasions [3], infections in mouse model [4] and in cell injury [5], suggesting iron to play a vital role in the virulence of *C. albicans*.

Recent studies confirmed that frequently encountered multidrug resistance (MDR) in *C. albicans* is a multifactorial phenomenon. One of the major mechanisms of MDR phenotype in *C. albicans* cells is characterized by an over-expression of genes encoding ATP Binding Cassette (ABC) such as *CaCDR1/CaCDR2*) [6], [7] or Major Facilitators (MFS) such as *CaMDR1* multidrug transporters [8], [9].There are, however, reported MDR mechanisms which appear to be independent of drug efflux pumps. For example, low levels of a homolog of bacterial two-component response regulators *SSK1* display enhanced sensitivity to drugs in *Candida* cells [10]. The morphogenic regulator *EFG1* levels affect drug susceptibilities of *C. albicans* cells [11]. Additionally, there are azole resistant clinical isolates of *C. albicans* where mechanisms of resistance appear to be different than the commonly known strategies adopted by *Candida* [12]. In this context, we recently reported that cellular iron status affects drug susceptibilities of *C. albicans* cells. It was observed that iron deprivation enhanced membrane fluidity and passive diffusion of drugs leading to increased drug susceptibility of *C. albicans* cells [13].

MDR in *Candida* is also closely linked to the status of membrane lipids. It has been already established that the associated changes in membrane lipid composition (phospholipid and ergosterol, in particular), its order (fluidity), and asymmetry are important determinants in the drug susceptibilities of yeast cells [14]-[16]. The action of antifungal agents is modulated by subtle modification of the membrane lipid composition [17]. It has been demonstrated that the interactions between membrane ergosterol and sphingolipids are critical and reduction in the content of either of these two components results in enhanced drug susceptibilities of *C*. *albicans* cells [15].We also observed a link between membrane fluidity and ergosterol in iron deprived *Candida* cells [13].

In the present study, we employed cDNA microarray based transcriptome and electrospray ionization tandem mass spectrometry (ESI-MS/MS) based lipid profiling, to evaluate relationship between iron homeostasis, membrane perturbations and drug susceptibilities in *Candida* cells. Our data revealed that previously observed increased drug susceptibilities of iron deprived *Candida* cells is mediated through cellular stress regulating calcineurin signal cascade. The lipid profiling of iron deprived *Candida* cells complemented transcriptome results and demonstrate a close relationship between cellular iron, lipid homeostasis and calcineurin signaling.

## Results

### Global transcriptional response to iron deprivation

To gain an insight into the possible mechanisms involved in iron deprivation induced drug susceptibility, we performed the transcriptional profiling which revealed that host of genes were differentially regulated in response to iron deprivation that could be grouped into various functional categories (www.candida.bri.nrc). A total of 365 genes were down regulated and 175 genes displayed up regulation (Fig S1). The details of gene categories, their description and their mean log_2_ fold expression values are given in Tables S1 and S2.

The global response depicted that cellular iron pools are tightly regulated and cells have evolved regulatory mechanisms not only to maintain iron homeostasis but also to prevent futile expression of the iron dependent pathways. We observed a typical transcriptional signature profile where iron deprivation leads to down regulation of several genes of TCA cycle (*ACO1, SDH12, IDH2, IDP2)* and oxidative phosphorylation *(QCR7, QCR2, LSC1, NDH51, COX9, LSC2, SDH2, CYT1, CYC1, CYB5* and *NUC2).* Since many enzymes critical to cellular respiration are dependent upon iron and therefore reduced iron availability would limit cellular energy. The cells appear to adapt to iron deficiency in two ways. Firstly, in response to iron deprivation, cells switched to fermentation mode of metabolism by up regulating several genes involved in carbohydrate metabolism particularly of glycolysis (*PGI1, FBP1, GLK4, ADH1*, *ADH5* and *IFE2*) and on the other hand, cells down play energy driven ribosomal protein synthesis machinery. Notably, 14% of the down regulated genes belonged to this category (Table S2). Exposure to iron deprivation also elicited changes in the expression of cell stress related genes. Among these were up-regulated genes included *DDR48*, *TRP99*, *ECM4*, *ASR1*, *ASR2*, and *ASR3* along with the heat shock proteins *HSP78*, *HSP70* and *HSP30*. The *RHR2*, *ALO1*, *SOD2*, *YHB1*, *YFH1*, *DAP1, HSP90* and *HSP60* were among the down-regulated stress response genes (Table S2).

**Figure 1:**
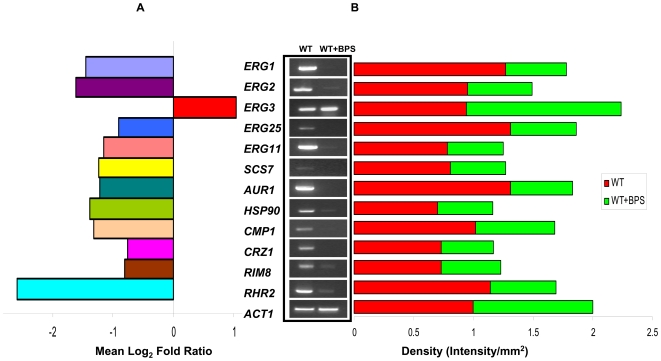
Iron Deprivation Responsive Genes. (**A**) Mean log_2_ fold expressions of genes differentially regulated in response to iron deprivation. Fold expressions of iron deprived vs control transcripts are depicted as mean log_2_ values, where values of 1.0 and -1.0 represent two fold up and down regulation, respectively. (**B**) RT-PCR of differentially regulated genes *ERG1*, *ERG2 ERG3, ERG25 ERG11, SCS7, AUR1, CMP1, HSP90, CRZ1, RIM8* and *RHR2* in response to iron deprivation in the WT *Candida* cells. The left panels show transcript levels in lanes (1) WT, (2) WT + BPS. The right panel shows the quantitation of the respective transcripts expressed as intensity per mm^2^ normalized with control *ACT1* transcript.

Interestingly, in addition to regulating several genes directly related to iron metabolism, we could detect some significant changes in genes related to MDR. For instance, among iron deprivation responsive genes in *Candida* cells included calcineurin signaling pathway which is known to be involved in drug susceptibilities in *Candida* cells (discussed below). Moreover, the status of lipid metabolism genes also deserves special mention. The down regulated genes included those involved in ergosterol biosynthesis (Fig. 1A and B) which also confirmed our previous observation that iron deprivation of *Candida* cells leads to a decrease in ergosterol levels [13]. Additionally, sphingolipid biosynthetic genes such as *AUR1* and *SCS7* were also down regulated (Fig. 1A and B). However, most of the other phospholipid biosynthesis genes did not appear to respond to iron status since their transcript levels did not change significantly. To decipher further the roles of calcineurin and lipid homeostasis in iron deprivation, these were examined closely.

### Iron deprivation reveals a link with calcineurin signaling pathway

Our transcriptome analysis as validated by RT-PCR revealed a link between cellular iron homeostasis and calcineurin pathways (Fig. 1A and B). Calcineurin is a highly conserved calcium dependent serine/threonine-specific protein phosphatase that mediates various stress responses inside the cell [18] and confers tolerance to azoles and other drugs targeting ergosterol biosynthesis in *C. albicans*. The downstream effector responses are partially governed by the calcineurin-dependent dephosphorylation and nuclear localization of the transcription factor Crz1p [19]. We observed that iron deprived *Candida* cells caused down regulation of not only the transcription factor *CRZ1* but also of upstream calcineurin genes encoding *CMP1* (encodes catalytic A subunit) and *HSP90* [20] which is a key regulator of calcineurin mediated azole resistance (Fig. 1A and B). Notably, several genes which included those involved in adaptation to alkaline pH viz. *RIM8*, salinity stress viz. *RHR2* and membrane perturbation stress viz. *ERG1*, *ERG2, ERG11, ERG25, AUR1* and *SCS7* were also down regulated (Fig. 1A and B).

### Iron deprivation phenocopies calcineurin signaling mutants

**Figure 2:**
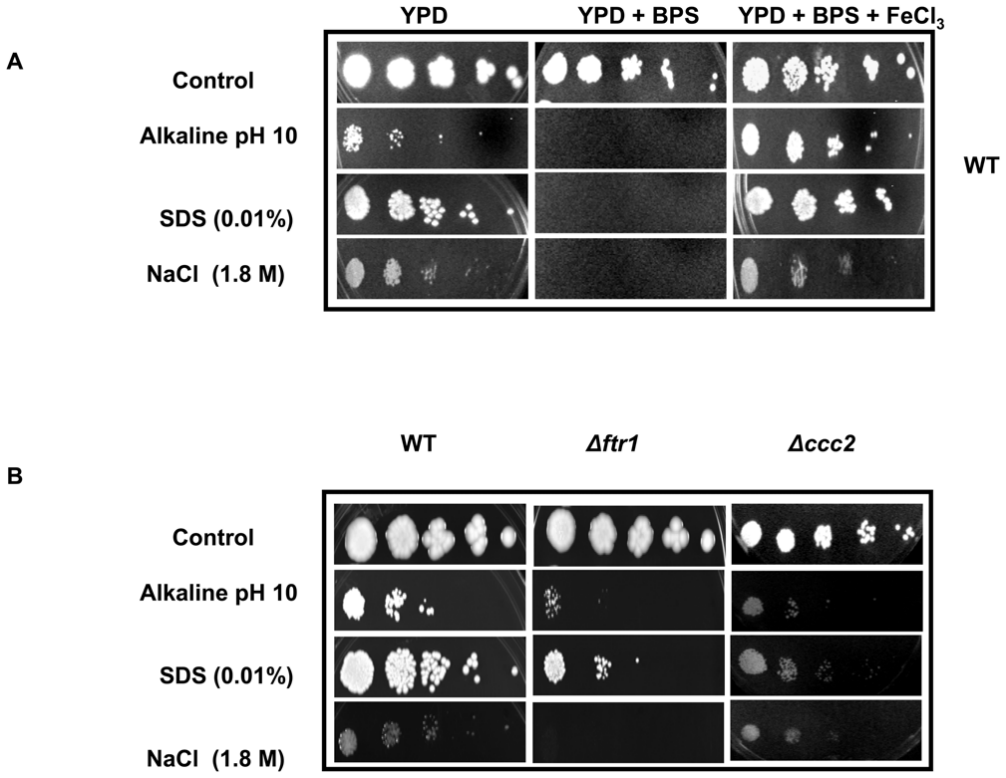
Phenotypic susceptibility assays of (A) WT (B) *Δftr1* and *Δccc2*cells, respectively as determined by spot assays described in Material and Methods in the absence (control) and presence of BPS.

We explored if the iron deprivation mediated down regulation of calcineurin signaling would also phenocopy the changes associated with the calcineurin pathway compromised cells. For this, we performed the phenotypic susceptibility assays under the conditions which required functional calcineurin. We observed that iron deprived WT cells [21] became hypersensitive to alkaline pH, salinity and membrane perturbation stresses (Fig. 2A). For example, in contrast to WT cells, iron deprived cells were hypersensitive to alkaline pH 10, sodium chloride (1.8M) and Sodium dodecyl sulphate (SDS) (0.01%) (Fig. 2A). Interestingly, the hypersensitivity associated with iron deprivation could be reversed upon iron supplementation (Fig. 2A). The role of iron in calcineurin signaling in *C. albicans* was further confirmed when two iron acquisition defective mutants such as *Δftr1* (defective in high-affinity iron uptake) [4] and *Δccc2* [defective in copper transport which is the essential component of the multicopper oxidase (*FET3*) and is required for high-affinity iron uptake] [22] were closely examined. Notably, both the iron-transport defective mutants were hypersensitive to alkaline pH 10, sodium chloride (1.8M) and SDS (0.01%) (Fig. 2B).

**Figure 3:**
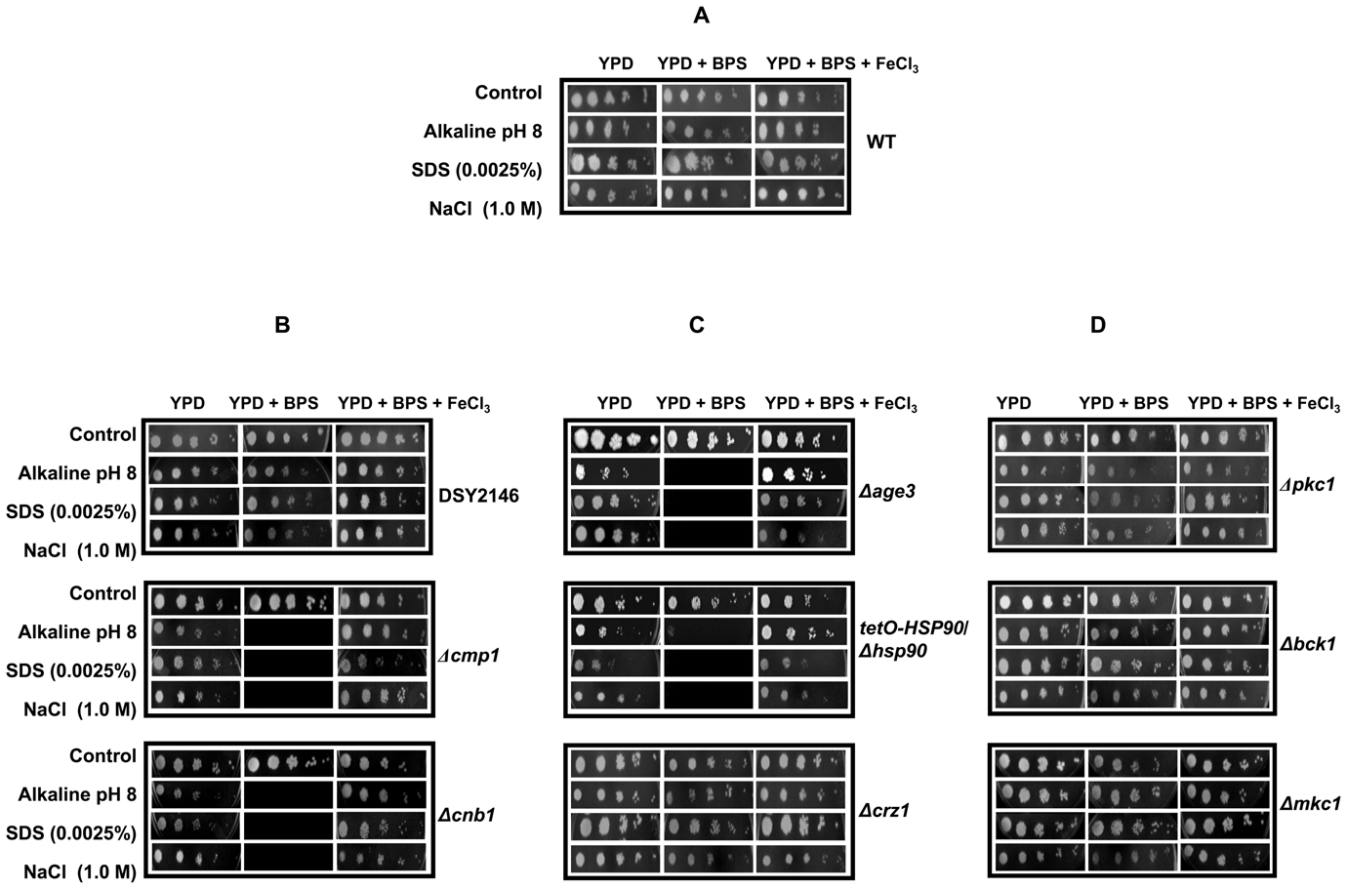
Phenotypic susceptibility assays of (A) WT (B) DSY2146, *Δcmp1* and *Δcnb1* cells, (C) *Δage3, tetO-HSP90*/*Δhsp90* and *Δcrz1* and (D) *Δpkc1, Δbck1* and *Δmkc1* cells, respectively as determined by spot assays as described in Material and Methods in the absence (control) and presence of BPS.

That iron deprivation phenocopies compromised calcineurin signaling pathway became further apparent with iron chelator bathophenanthroline disulphonic acid (BPS) treated calcineurin mutants such as *Δcmp1* (catalytic A subunit), *Δcnb1* (regulatory B subunit) and with calcineurin strain having hyperactive allele (DSY2146) [23]. Notably, since these mutant strains (*Δcmp1* and *Δcnb1*) were already hypersensitive to alkaline pH 10, sodium chloride (1.8M) and SDS (0.01%), similar threshold levels of these stresses could not be used to evaluate the effect of iron depletion. For this, we lowered the threshold of stresses like alkaline pH (from 10 to 8), SDS (0.01% to 0.0025%) and NaCl (1.8M to 1.0M). At indicated lower thresholds of various stresses, these mutant cells (including other mutants such as *Δage3,* conditional *Δhsp90* and *Δcrz1* discussed below*)* could grow reasonably well which in contrast to the WT cells (Fig. 3A), became hyper susceptible to indicated stresses upon BPS treatment (Fig. 3B). Furthermore, in contrast to calcineurin mutants namely *Δcmp1* and *Δcnb1*, a DSY2146 strain containing hyperactive allele of calcineurin remained resistant towards alkaline pH 8, sodium chloride (1M) and SDS (0.0025%) under iron deprivation (Fig. 3B).

### Iron deprivation affects other effectors of calcineurin signaling pathway

We examined if the iron deprivation mediated loss in calcineurin dependent phenotypes was due to the involvement of any of the other key regulators which crosstalk with calcineurin signaling pathway. For this, the phenotypic susceptibility assays were done with various such mutants under the similar conditions which required functional calcineurin. Firstly, we used null mutant of *AGE3* (*Δage3)*, which codes for an ADP-ribosylation factor (ARF) GTPase activating effector protein and which has been shown to be responsible for abrogation of drug tolerance in *C. albicans.* Moreover, even in strains where calcineurin pathway is stimulated or constitutively expressed, *AGE3* dependent azole sensitivity is not rescued [24]. Our results show that as compared to the WT cells [25], iron deprived *Δage3* became highly sensitive to alkaline pH, salinity and membrane perturbation which could be reversed upon iron supplementation (Fig. 3C). Calcineurin is a client protein of Hsp90 in *C. albicans* and compromising Hsp90 phenocopies abrogated calcineurin pathway [26]. Given its role in diverse stress response, we postulated that this chaperone might also regulate crucial responses to iron deprivation. Interestingly, we observed that as compared to the WT cells, iron deprived conditional *HSP90* nulls (*tetO-HSP90/Δhsp90*) were sensitive to alkaline pH, salinity and membrane perturbation which could be reversed upon iron supplementation (Fig. 3C). The regulator *CRZ1* is the downstream transcription factor which governs the expression of calcineurin dependent genes. To determine whether *CRZ1*, is also an effector of the above observed sensitivities to iron deprivation, we tested *Δcrz1* [27]. Notably, as compared to the WT cells, iron deprived *Δcrz1* did not show any affect on its sensitivity to alkaline pH, salinity and membrane perturbation (Fig. 3C).

### Iron deprivation does not crosstalk with cell wall integrity (CWI) signaling pathway

Another key cellular stress response pathway having implications in basal tolerance to azoles is the protein kinase C (PKC) mediated cell wall integrity pathway (CWI). A recent study established a novel role of CWI and calcineurin signaling pathway in *C. albicans* [28]. We explored whether CWI pathway cross talks with iron homeostasis pathway which phenocopies calcineurin mutants. For this, we tested if MAP kinase mutants namely *Δpkc1*, *Δbck1* and *Δmkc1* of CWI pathway were sensitive to alkaline pH, salinity and membrane perturbations. Our results depicted in Fig. 3D show that kinase mutants unlike calcineurin mutants were not sensitive to alkaline pH, salinity and membrane perturbations upon iron deprivation.

### Iron deprivation leads to global remodeling of cellular lipids

Our transcriptome data suggested a possible link between membrane stress (calcineurin mediated signaling) cellular iron and drug resistance. Moreover, it was also evident from our earlier [13] as well as from present studies (Fig. 1A and B) that iron deprivation also targets lipid metabolic pathways particularly those of ergosterol and sphingolipid metabolism. Therefore, by employing high throughput MS based lipid profiling, we evaluated the impact of iron deprivation in lipid homeostasis. We ensured that lipidome analysis strictly matched conditions that were used for transciptome analysis (see Methods). As described in Methods, *C. albicans* cells were harvested and their total lipids were extracted by employing modified Bligh and Dyers method [29], [30]. The extracted lipids were subjected to electron spray ionization tandem mass spectrometry (ESI-MS/MS) by direct infusion of the lipid extracts into mass spectrometer. The total lipids were quantified and at the level of absolute quantification. The most abundant lipid group was Phosphoglyceride (PGL) and was found to range from 775 to 868 nmol per mg dry lipid weight. The list of all the differentially expressed lipid species are mentioned in Tables S3, S4, S5, S6, S7.

#### Sterol homeostasis is disrupted upon iron deprivation

**Figure 4:**
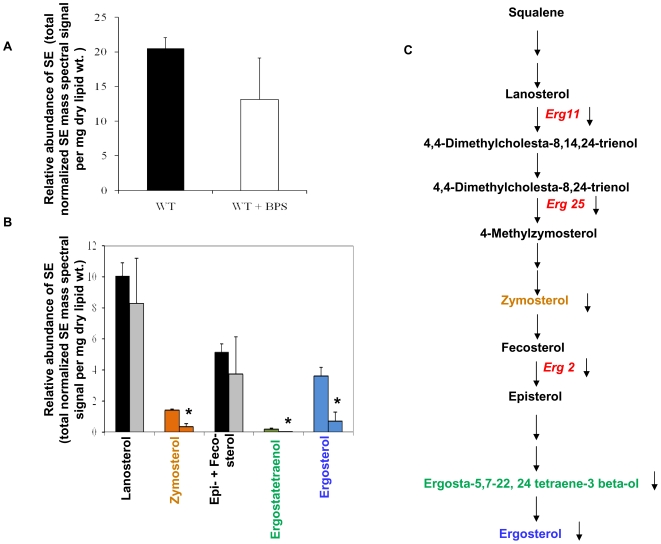
Sterol Composition of Iron Deprived Cells. **A.** The total SE content (as total normalized SE mass spectral signal per mg dry lipid wt.) in *Candida* (WT) cells in absence and presence of BPS. **B.** The relative abundance of SE intermediates (as total normalized SE mass spectral signal per mg dry lipid wt.) in *Candida* (WT) cells in absence and presence of BPS. Total for each SE class was calculated by adding the nmole content of the molecular lipid species of that class. Values are means ± SD (n = 3, 3 independent analyses of lipid extracts from 3 independent cultures and * depicts *P* value<0.05). **C.** Ergosterol pathway showing iron deprivation responsive genes in red and the affected intermediates whose colour code matches with bars in Fig 4B.

Our lipidome analysis revealed that the total sterol levels were decreased (by 36%; *P* = 0.053) in response to iron deprivation (Fig. 4A **)**. Additionally, apart from considerable decrease in ergosterol levels (∼80%), there was also a significant decrease in some of the intermediates of ergosterol biosynthetic pathway viz. zymosterol, and ergostatetraenol, ∼75% and 93%, respectively (Fig. 4B). Of note, the levels of other intermediates of ergosterol pathway were also decreased but this change was not statistically significant under our experimental conditions (*P*>0.05). Interestingly, our microarray results which showed considerable down regulation of many ergosterol biosynthetic pathway genes (Fig. 4C) in iron deprivation and also validated by RT-PCR complemented lipidome data (Fig. 1A and B).

Esterified sterols, a core component of lipid storage particles, represent a major storage of cellular sterols in most eukaryotes, including yeast [31], [32]. To determine if lipid storage was affected by iron deprivation, we estimated the total sterol ester composition and observed that there was a decrease in total mono- and poly- unsaturated fatty acyl (FA) chain containing sterol esters in iron deprived cells For example, a significant decrease in total 16∶1, 18∶3 and 18∶1 containing sterol esters by ∼1.5 to 2.5 folds was observed in iron deprived cells (Table S5).

#### Iron deprivation affects Sphingolipid Composition by inhibiting IPC synthesis

**Figure 5:**
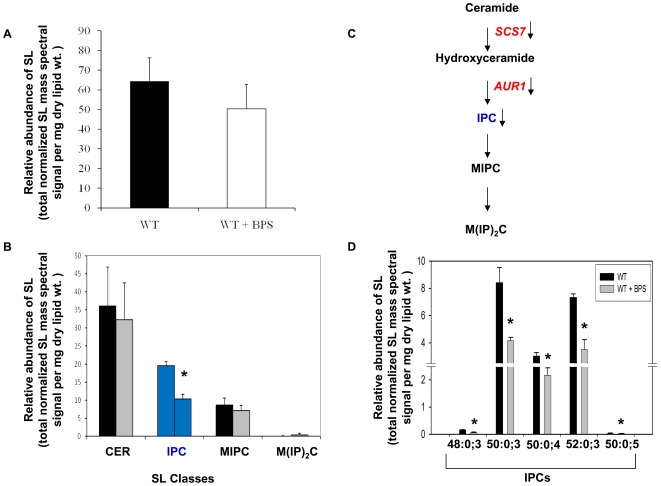
Sphingolipid Composition of Iron Deprived Cells. **A.** The relative abundance of SLs (as total normalized SL mass spectral signal per mg dry lipid wt.) in *Candida* (WT) cells in absence and presence of BPS. **B**. The relative abundance of SL classes (as total normalized SL mass spectral signal per mg dry lipid wt.). The total for each SL group was calculated by adding the normalized mass spectral signal of molecular lipid species of that group. Error bars indicate ± SD. (n = 3, 3 independent analyses of lipid extracts from 3 independent cultures). **C.** Sphingolipid pathway showing the iron responsive genes in red and accumulated intermediate IPC in blue which matches with bars in Fig 5B. **D.** Molecular lipid species compositions of IPC (as as total normalized SL mass spectral signal per mg dry lipid wt.) in *Candida* (WT) cells in the absence and presence of BPS. Only the major molecular lipid species (*P* value<0.05) are depicted in this figure. Values are means ± SD (n = 3 and * depicts *P* value<0.05).

Using sphingolipid (SL) profiling by ESI-MS/MS as discussed in Materials and Methods, our analysis detected total of 23 molecular SL species of ceramide (CER) (2 species), inositolphosphorylceramide (IPC) (9 species), mannosylinositolphosphorylceramide (MIPC) (8 species) and mannosyldiinositolphosphorylceramide [M(IP)_2_C] (4 species) (Table S4). SL molecular species are represented as “total number of carbons in the sphingoid base and acyl chains: total number of carbon-carbon double bonds in the sphingoid base and acyl chains; number of hydroxyl groups present in the sphingoid base and acyl chains”. No significant change in total sphingolipid content of the cell was observed in iron deprived cells (Fig. 5A). However, the lipidome of iron deprived cells showed specific changes in the molecular composition of some SL species. We found that there was no major change in the CER levels; however, total IPC levels were decreased significantly by 47% (Fig. 5B). This was also evident by transcriptome analysis in iron deprivation which showed considerable down regulation of *AUR1* gene, as validated by RT-PCR, which encodes for IPC synthase (Fig. 1A and B; 5C). Out of the individual IPC species, the two most abundant IPCs: IPC 50∶0;3 and IPC 52∶0;3 were down regulated by more than two folds while the minor species viz IPC 48∶0;3, IPC 50∶0;4 and IPC 50∶0;5 were down regulated between 1.4 and 2.7 folds (Fig. 5D). There were no significant changes observed in total (MIPC) and M(IP)_2_C levels, or in their respective species under iron deprived condition (Fig. 5B).

#### Iron deprivation causes minor fluctuations in Phosphoglyceride composition

**Figure 6:**
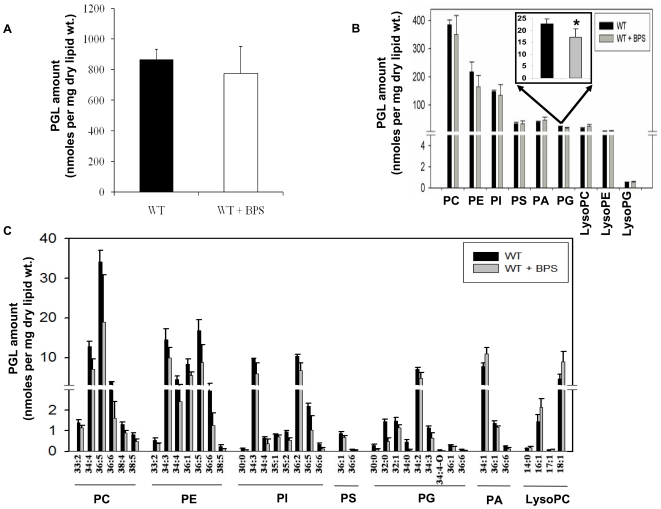
Phosphoglyceride Composition of Iron Deprived Cells. **A.** The amount of total PGLs (as nmoles per mg dry lipid wt.) in *Candida* (WT) cells in the absence and presence of BPS. **B.** The composition of PGL classes (as nmoles per mg dry lipid wt.) in *Candida* (WT) cells in absence and presence of BPS. The total for each PGL class was calculated by adding the nmoles content of the molecular lipid species of that class (* depicts *P* value<0.05). The inset highlights the magnitude of statistically significant change in PG levels in response to iron deprivation. **C.** Molecular lipid species composition of PGLs (as nmoles per mg dry lipid wt.) in *Candida* (WT) cells in absence and presence of BPS. Only the major molecular lipid species (*P* value<0.05) are depicted in this figure. Error bars indicate ± SD. (n = 3, 3 independent analyses of lipid extracts from 3 independent cultures).

The MS analysis targeted nine major PGL classes whose lipid molecular species were identified by mass of the head group plus the mass of the intact lipid, allowing determination of the number of carbon atoms (C atoms) and double bonds in the acyl chain(s) of PGLs. The relative abundance of the diacyl phospholipids was in order phosphatidyl choline (PC), phosphatidyl ethanolamine (PE), phosphatidyl inositol (PI), phosphatidyl serine (PS), phosphatidyl acid (PA) and phosphatidyl glycerol (PG), with PC, PE, and PI accounting for ∼80% of the PGLs under all conditions. PCs were the predominant PGLs in *C. albicans*, accounting for ∼45% (% of total PGLs). This was followed by PE and PI, with each accounting for around 17 to 25% (% of total PGLs). The levels for the three minor species, PS, PA, and PG, were all <10% (% of total PGLs). The minor species of PGLs like lysophophatidylcholine (LysoPCs), lysophophatidylglycerol (LysoPGs) and lysophophatidylethanolamine (LysoPEs) which account for less than 5% (% of total PGLs) were also detected. Notably, no significant differences among most of the PGL groups was observed (Fig. 6A) except in, total PG levels which were decreased significantly (by ∼25%) in response to iron deprivation (Fig. 6B). There are evidences which suggest that lack of anionic phospholipids like PG compromises the osmotic tolerance and mitochondrial functions as depicted by reduced cytochrome levels and respiratory chain activity in yeasts [33], [34]. Our transcriptome results show that several mitochondrial genes along with those for osmotic tolerance were down regulated (Table S2).It was observed that the cell undergoes some PL remodeling in its composition in response to iron deprivation as depicted by variation in molecular species. Mass spectrometry analysis revealed that 37 species of PGLs varied significantly in response to iron deficiency, which included 6 species of PCs, 7 species of PEs, 8 species of PIs, 2 species of PSs, 8 species of PGs, 3 species of PAs and 4 species of LysoPCs (Fig. 6C). Most of the molecular PL species showed significant decrease in amount upon iron deprivation except lyso-lipid species that were present in higher amounts (Fig. 6C). Overall, as compared to other groups of lipids analyzed, changes in PGLs upon iron deprivation are of minor modifications in nature. These changes corresponded well to transcriptome analysis, as no PGL metabolism genes were found to be differentially expressed in response to iron deficiency.

## Discussion

We previously demonstrated a relationship between cellular iron status, membrane perturbations and drug susceptibility of *C. albicans* cells. [13]. To gain further insights into the possible mechanisms, we explored global response of iron deprived *C. albicans* cells through genome based microarray analyses. The transcriptome data under iron limiting conditions suggested that *Candida* cells not only increased the uptake of iron by up regulating iron acquisition genes viz. *FTR1, FET34, SIT1, HMX1, FRP1* and *FTH1* but also prioritized its utilization in a manner that iron independent enzymes or metabolic pathways are preferably utilized over iron dependent enzymes. Thus iron deprived *Candida* cells are able to divert iron that would otherwise be incorporated into these pathways to other essential pathways such that metabolic homeostasis is maintained. Moreover, differential regulation of many of the stress responsive genes such as (*DDR48*, *TRP99*, *ECM4*, *ASR1*, *ASR2*, *ASR3, HSP78*, *HSP70, HSP30, RHR2*, *ALO1*, *SOD2*, *YHB1*, *YFH1*, *DAP1, HSP90* and *HSP60*) upon iron deprivation suggests that *Candida* cells perceive iron scarcity as a nutritional stress.

Most strikingly, we could detect a possible linkage between conserved calcineurin signaling pathway and iron homeostasis. It was observed that iron deprivation down regulates genes encoding the catalytic subunit of calcineurin *CMP1* along with its downstream transcription factor *CRZ1* and genes such as *RIM8*, *RHR2, ERG1*, *ERG2, ERG11, ERG25, AUR1* and *SCS7* mediating functions such as adaptation to alkaline pH, salinity and membrane stresses (Fig. 1A and B). Our phenotypic susceptibility assays in response to iron deprivation in presence of various calcineurin dependent stresses validated the microarray array data (Fig. 2A). Moreover, the mutants defective in iron acquisition such as Δ*ftr1* as well as copper transporter mutant Δ*ccc2*, which affects the high affinity iron uptake in *Candida* also showed hypersensitivity without the addition of BPS (Fig. 2B). Furthermore, in contrast to calcineurin mutants such as *Δcmp1* and *Δcnb1* which become hyper susceptible to above stresses when deprived of iron, calcineurin strain having hyperactive allele remained resistant (Fig. 3B). Thus the findings that both pharmacological and genetic blockage of iron intake resulting in hypersensitivity towards alkaline pH, salinity and membrane perturbation stresses clearly establishes a novel linkage of calcineurin signaling with iron homeostasis.

That iron mediated drug susceptibilities cross talk with calcineurin signaling was further established from other set of mutant analysis. For instance, both *AGE3* and *HSP90* are known to regulate drug susceptibilities in *C. albicans* and also cross talks with calcineurin circuitry [24], [26]. Interestingly, we observed that both *AGE3* and conditional *HSP90* (*tetO-HSP90*/*Δhsp90*) null mutants, if deprived of iron, phenocopies calcineurin mutants and thus show sensitivity towards alkaline pH, salt and membrane perturbations that could be reversed by the supplementation of iron (Fig. 3C). However, despite of down regulation of the down stream regulator of calcineurin signaling encoding gene *CRZ1*, it did not appear to have any significant role in iron mediated stress responses as the phenotypic consequences in *Δcrz1* mutant differs from that of calcineurin and conditional *HSP90* mutants (Fig. 3C). This is in agreement with earlier observations that *CRZ1* only modestly governs drug resistance downstream to calcineurin and probably has no major role in some of other effectors functions. [35], [36]. Interestingly, the PKC mediated CWI pathway seems to be functionally dispensable for the phenotypes governed by iron deprivation since compromised PKC signaling mutants had negligible affect on calcineurin dependent stress responses under iron deprivation (Fig. 3D). Given the importance of calcineurin in the virulence of *C. albicans* [37], [18], its iron mediated regulation is an interesting aspect which merits further evaluation.

Lipids are considered to be the most adaptable molecules in response to environmental changes and targets for stress adaptation [38].Several observations such as relationship between iron availability, membrane fluidity and drug susceptibilities of *C. albicans* cells [13], functional indispensability of calcineurin to sustain membrane stress [39] and a possible link of calcineurin with iron homeostasis (present study), necessitated an in depth analyses of lipid profile of iron deprived *Candida* cells. Considering that iron deprived cells had decreased levels of ergosterol [13] and that several *ERG* genes are down regulated (this study), we particularly examined the effects of iron deprivation on ergosterol biosynthetic intermediates. The levels of sterol intermediates in cells grown in iron-deficient media, not only confirmed a decrease in ergosterol levels as compared to WT cells (Fig. 4B), but also exhibited depletion of the other major intermediates of the ergosterol pathway like zymosterol and ergostatetraenol. The levels of various ergosterol pathway intermediates suggested that iron deprivation inhibits several biosynthetic genes including the azoles target *ERG11*. This is well supported by our previous [13] as well as present observations that iron deprivation causes down regulation of *ERG11* and a parallel up regulation of *ERG3*. (Fig. 1A and B). Notably, the down regulation of ergosterol biosynthetic genes could be explained if one considers the fact that post-lanosterol step also includes iron dependent enzymatic steps. The down regulated *ERG11* and *ERG25* genes which encode a haem dependent enzyme of the cytochrome P450 family, and an iron containing enzyme of the fatty acid hydroxylase desaturase family, respectively [40], [41] are the two such examples. The reduced ergosterol could also be attributed to the fact that the levels of haem binding protein Dap1p required for Erg11p [42], [43] and heme biosynthesis encoding genes such as *HEM1, HEM3, HEM4* and *HEM14* are also compromised in iron deprived cells (Table S2).

That iron deficiency stress perturbs membrane structure and function was further evident from the fact that sphingolipid biosythesis of *Candida* was also compromised. It was more reflected in significantly changed sphingolipid composition (Fig. 5B) rather in its total content (Fig. 5A). For example, IPC levels under iron deprivation were significantly reduced while ceramide levels remained unchanged (Fig. 5B). Consistent with this, IPC synthase encoded by *AUR1* which is also exploited as an antifungal target [44], [45], was correspondingly down regulated (Fig. 1A and B). The decrease in IPC levels was not reflected in the levels of down stream products such as MIPC and M(IP)_2_C, however, we could detect significant differences in composition of IPC molecular species under iron deprivation (Fig. 5D). This could be due to the fact that M(IP)_2_C can be synthesized independent of *IPT1* mediated enzymatic step in sphingolipid biosynthetic pathway [46] which converts MIPC to M(IP)_2_C. Our results support an earlier study [47] where it was shown that IPC synthesis could also regulate the generation of DAG and cell cycle progression. Interestingly, our transcriptome results also depicts the down regulation of genes involved in cell cycle progression (Table S2) which could be correlated with the observed decrease in DAGs in iron deprived cells. In an earlier study, *Cryptococcus neoformans* deficient in IPC synthase was shown to contain reduce levels of DAG which lead to enhanced phagocytosis by alveolar macrophages and reduced pathogenecity [48]. Since, iron deprivation of *Candida* cells also results in decrease levels of IPC with a simultaneous down regulation of *AUR1*, it may account for lower DAG levels. The compromised biosynthetic pathways of ergosterol and sphingolipid biosynthesis under iron deprivation, which are also major contributors of membrane rafts, further highlights the interdependence of homeostasis between membrane rafts constituents [49].

Taken together, for the first time, we demonstrate a link between drug susceptibility of iron deprived *C. albicans* with calcineurin signaling pathway. Transcriptome and complimentary lipidome profile show that iron deprivation leads to down regulation of calcineurin pathway leading to hypersensitive responses to stresses including membrane perturbations and subsequent remodeling of membrane lipids, thereby making *Candida* cells susceptible to drugs.

## Materials and Methods

Media chemicals were obtained from Difco (Detroit, Mich., USA) and HiMedia (Mumbai, India). Bathophenathrolin disulphonic acid (BPS), Sodium Chloride (NaCl), Sodium dodoceyl sulphate (SDS) and Ferric Chloride (FeCl_3_) were obtained from Sigma Chemical Co. (St. Louis, Mo., USA). Synthetic lipids with FA compositions that are not found or are of very low abundance in *Candida* were used as internal standards. Lipid standards were obtained from Avanti Polar Lipids (Alabaster, AL).

### Growth media and strains used

*C. albicans* strains used in this study are listed in Table S8. The *C. albicans* strains were stored in 15% (*v/v*) glycerol stock at -80°C. The cells were freshly revived on YEPD (1% yeast extract, 2% glucose, and 2% bactopeptone) plates from this stock before each experiment. For iron depletion we used BPS, a well-known iron chelator at a concentration (150 µM) that depleted iron from the media without affecting the growth of the *Candida* cells. We measured intracellular iron levels by the enzymatic method using Calcein-AM, a fluorescent dye to establish iron-deficient conditions (Fig S2) [50]. The cells were diluted into 50 ml fresh YEPD broth at OD_600_ of 0.1 (∼10^6^ cells/ml) in absence and presence of BPS (150 µM), and grown at 30°C till OD_600_ of 1.0. Cells were harvested by transferring the cells into a centrifuge tube. All the experiments were carried out on cells that were iron-replete to begin with and they suffered iron stress only during the experiment. Three separate experimental replicate cultures of each condition were used.

### RNA Isolation

RNA isolation was done essentially by following Trizol (Sigma) method as per manufacturer's specifications, except that 300 µl acid washed 0.4-0.6 mm glass beads (Sigma, St. Louis, MO) were used during cell lysis. RNA was precipitated using absolute ethanol and washed twice with 80% ethanol, dried and resuspended in 50 µl DEPC treated water at 58°C. RNA obtained was quantitated spectrophometrically and it was also electrophoresed in denaturing formaldehyde gel.

### cDNA synthesis and Hybridization

10 µg of purified total RNA was used to synthesize cDNA by using the protocol described in www.transcriptome.ens.fr/sgdb/protocols/labelling.yeast.php. We used direct-labeling method to label cDNAs with Cy3 and Cy5 dyes. Labeled cDNA was then purified using Qiaquick PCR purification columns with final elution volume of 30 µl. Labeled cDNA was mixed with hybridization buffer and applied immediately on the microarray slide. It was covered with 24*60 mm coverslip, sealed in Corning hybridization chambers and allowed to incubate overnight at 42°C. Slides were washed and scanned. Each experimental condition was independently repeated thrice including a dye swap.

### Scanning and Data Analysis

Slides were scanned using Scan Array Express microarray scanner from Perkin Elmer. Raw data obtained was quantitated using adaptive circle method and normalized using LOWESS. The values obtained by this was background corrected and ratios of treated versus untreated intensity were converted in Log_2_ ratios which was averaged for replicates of same set of experiment. We have used www.candida.bri.nrc website to obtain the annotations for the ORFs. GO cluster were found using GO term finder option in www.candidagenome.org. Threshold value was set to two-fold to obtain affected genes which were statistically significant. However, some of the important differentially responsive genes (for this study) which were between 1.5 to 2.0 fold were also included. Notably, the additional few genes (e.g. *ERG25* (1.9 fold), *CRZ1* (1.5 fold) of lower threshold cut off were also included since they fell under highly responsive categories of genes which were 2 folds and above. All the responsive genes were subsequently validated by RT-PCR (Fig. 1B).

### Microarray Data accession number

Microarray data used in this study is fully described in MIAME compliant database, GEO and the raw as well as normalized data files has been deposited under accession number GSE25394.

### RT-PCR

RT-PCR was done using the RevertAid™ H Minus kit (MBI, Fermentas).Briefly, 1 µg isolated RNA was DNase treated at 37°C for 30 min and reaction was terminated by adding 1 µl of 25mM EDTA and incubated at 65°C for 60 min. RNA was subsequently primed with oligo (dT)_18_ for cDNA syntheis at 42°C for 60 min. Reverse transcription reaction was terminated by heating at 70°C for 5 min. The synthesized cDNA product (2 µl) was directly used for PCR amplification reaction (50 µl) using gene specific forward and reverse primers (Table S9). The amplified products were gel electrophoresed and quantitated as described in figure legends.

### Phenotypic susceptibility assays

Phenotypic susceptibilities were measured using spot assays [13], [14]. The following stock solutions were used (the solvents used are given in parenthesis): BPS, 100mM (water); SDS, 10% (water); NaCl, 5M (water); FeCl_3_, 100mM (water). The final chemical concentrations used for this study are specified below. In spot assay, five microlitres of five fold serial dilutions of each yeast culture (each with cells suspended in normal saline to an OD_600_ of 0.1) was spotted on to YEPD plates in the absence (control) and presence of the chemicals at the following concentrations: BPS (150 µM), FeCl_3_ (100 µM), alkaline pH 10.0, SDS (0.01%), NaCl (1.8M) for WT, Δ*ftr1*, Δ*ccc2* and BPS (150 µM), FeCl_3_ (100 µM), alkaline pH 8.0, SDS (0.0025%), NaCl (1M) for rest of all mutants. For alkaline pH, YEPD plates buffered with 155mM of Tris-Cl at pH 8.0 and 10 were used. Growth differences were recorded following incubation of the plates for 48 hours at 30°C.

### Lipid Extraction and ESI-MS/MS lipid profiling

Lipids were extracted from *Candida* cells using a slight modification of the method of Bligh and Dyer [29], [30]. An automated ESI-MS/MS approach was used. Data acquisition and analysis were carried out as described previously [30].The ESI-MS/MS procedure for SL quantification was similar to that for phosphoglyceride quantification. Lipid species were detected with the scans described in Table S10. The ESI-MS/MS procedure for SE (Sterol ester), DAG (Di-acyl glycerol) and TAG (Tri-acyl glycerol) quantification was similar to that for phosphoglyceride quantification. Precise amounts of internal standards were added in the quantities with some small variation in amounts in different batches of internal standards (Table S11).

### Statistical Analysis

The mean of three independent biological replicates ± standard deviation (SD) from the individual samples was used for transcriptome and lipidome analysis. To get a list of statistically significant genes, we combined the two methods commonly in use in most microarray articles: we applied sequentially a cutoff based on the gene expression variation and a cutoff based on the reproducibility of the measurement of this variation [51]. More precisely, the expression of a gene was considered significantly and reproducibly changed when satisfying both criteria. To assess the statistical significance of the difference in datasets, the Student *t*-test was performed using the significance level of 0.05. Any attribute was considered to be statistically significant and reported ‘increased’ or ‘decreased’ as a function of iron availability only if the *P* values was less than 0.05.

## Supporting Information

Figure S1 **Iron Deprivation Responsive Genes.** X- axis shows percentage of total genes (n = 540) falling into each category. Y-axis shows the various functional categories based upon www.candida.bri.nrc **A**. Distribution of up regulated genes in response to iron deprivation assigned to various functional categories. **B.** Distribution of down regulated genes in response to iron deprivation assigned to various functional categories.
(DOC)

Figure S2 **Labile Iron Pool (LIP) Measurement.** **A.** LIP was measured by Calcein-AM fluorescence method as described elsewhere [50]. Upper panel depicts the fluorescence intensity (higher fluorescence depicts higher iron chelation or less LIP) as measured by fluorescence microscope for WT, WT + BPS and WT + BPS + FeCl_3_ treated cell respectively. Lower panel depicts the phase contrast micrographs of the upper panel. (Magnification 63X). **B.** Quantitative measurement of LIP in WT, WT + BPS and WT + BPS + FeCl_3_ treated cells depicted on x-axis, as measured by Relative Fluorescence (Rf) values. Mean of the Relative Fluorescence (Rf) values ± S.D. of the three sets of experiments are depicted on y-axis.
(DOC)

Table S1 **Genes up regulated in response to iron deprivation.** Fold expressions of treated vs control are depicted as mean log_2_ values where a value of 1.0 represents two fold up regulation.
(DOC)

Table S2 **Genes down regulated in response to iron deprivation.** Fold expressions of treated vs control are depicted as mean log_2_ values where a value of -1.0 represents two fold down regulation.
(DOC)

Table S3 **PGL composition in response to iron deprivation.** Values are mean of ± SD (n = 3 for conditions, * depicts *P* value<0.05). Data is represented as nmoles/mg dry lipid weight (normalized mass spectral signal).
(DOC)

Table S4 **The relative abundance of SL compositions in response to iron deprivation.** Values are mean of ± SD (n = 3 for conditions, * depicts *P* value<0.05). Data is represented as nmoles/mg dry lipid weight (normalized SL mass spectral signal).
(DOC)

Table S5 **The relative abundance of SE compositions in response to iron deprivation.** Values are means of ± SD (n = 3 for conditions, * depicts *P* value<0.05). Data is represented as nmoles/mg dry lipid weight (normalized SE mass spectral signal).
(DOC)

Table S6 **The relative abundance of DAG based on fatty acid chain compositions in response to iron deprivation.** Values are mean of ± SD (n = 3 for conditions, * depicts *P* value<0.05). Data is represented as nmoles/mg dry lipid weight (normalized DAG mass spectral signal).
(DOC)

Table S7 **The relative abundance of TAG based on fatty acid chain compositions in response to iron deprivation.** Values are mean of ± SD (n = 3 for conditions, * depicts *P* value<0.05). Data is represented as nmoles/mg dry lipid weight (normalized TAG mass spectral signal).
(DOC)

Table S8 **List of** ***C. albicans*** **strains used in the study.**
(DOC)

Table S9 **List of primer sequences for RT-PCR used in the study.**
(DOC)

Table S10 **Scans used to detect the lipid species containing a common head group fragment.**
(DOC)

Table S11 **Amount of internal standard used for the lipid quantification.**
(DOC)
