# Supplementary material for: Calcineurin Signaling and Membrane Lipid Homeostasis Regulates Iron Mediated MultiDrug Resistance Mechanisms in Candida albicans
Source: PLoS One. 2011 Apr 12;6(4):e18684. doi: 10.1371/journal.pone.0018684 (PMC3075269; doi:10.1371/journal.pone.0018684)
Supplement: Figure S1 — Iron Deprivation Responsive Genes. X- axis shows percentage of total genes (n = 540) falling into each category. Y-axis shows the various functional categories based upon www.candida.bri.nrc A. Distribution of up regulated genes in response to iron deprivation assigned to various functional categories. B. Distribution of down regulated genes in response to iron deprivation assigned to various functional categories. (DOC) [file pone.0018684.s001.doc]

**Figure S1.**

**A**

**B**
