# Supplementary material for: Calcineurin Signaling and Membrane Lipid Homeostasis Regulates Iron Mediated MultiDrug Resistance Mechanisms in Candida albicans
Source: PLoS One. 2011 Apr 12;6(4):e18684. doi: 10.1371/journal.pone.0018684 (PMC3075269; doi:10.1371/journal.pone.0018684)
Supplement: Figure S2 — Labile Iron Pool (LIP) Measurement. A. LIP was measured by Calcein-AM fluorescence method as described elsewhere [50]. Upper panel depicts the fluorescence intensity (higher fluorescence depicts higher iron chelation or less LIP) as measured by fluorescence microscope for WT, WT + BPS and WT + BPS + FeCl3 treated cell respectively. Lower panel depicts the phase contrast micrographs of the upper panel. (Magnification 63X). B. Quantitative measurement of LIP in WT, WT + BPS and WT + BPS + FeCl3 treated cells depicted on x-axis, as measured by Relative Fluorescence (Rf) values. Mean of the Relative Fluorescence (Rf) values ± S.D. of the three sets of experiments are depicted on y-axis. (DOC) [file pone.0018684.s002.doc]

**Figure S2.**

**A**

**B**
