# Supplementary material for: Calcineurin Signaling and Membrane Lipid Homeostasis Regulates Iron Mediated MultiDrug Resistance Mechanisms in Candida albicans
Source: PLoS One. 2011 Apr 12;6(4):e18684. doi: 10.1371/journal.pone.0018684 (PMC3075269; doi:10.1371/journal.pone.0018684)
Supplement: Table S1 — Genes up regulated in response to iron deprivation. Fold expressions of treated vs control are depicted as mean log2 values where a value of 1.0 represents two fold up regulation. (DOC) [file pone.0018684.s003.doc]

**Table: S1**

| **Systematic Name** | **Standard Name** | **Description** | **Mean Log_2_ Fold Expression** |
| --- | --- | --- | --- |
| *Carbohydrate Metabolism* | | | |
| orf19.3888 | PGI1 | Protein described as glucose-6-phosphate isomerase, enzyme of glycolysis; antigenic in human; regulated by Efg1p; induced in biofilm, upon adherence to polystyrene; down regulated in the presence of human neutrophils, upon phagocytosis | 0.79 |
| orf19.7021 | GPH1 | Putative glycogen phosphorylase; gene regulated by Ssk1p, Mig1p, and Tup1p; fluconazole-induced; localizesto cell surface of hyphal cells, but not yeast-form cells; S. cerevisiae Gph1p is a stress-regulated protein of glycogen metabolism | 2.60 |
| orf19.4393 | CIT1 | Protein described as citrate synthase; soluble protein in hyphae; biofilm induced; expression greater in high iron; upregulated upon phagocytosis; Hog1p-downregulated; regulated by Efg1p under yeast-form but not hyphal growth conditions | 1.44 |
| orf19.3278 | GSY1 | Protein described as glycogen synthase; enzyme of glycogen metabolism; transcription down regulated upon yeast-hyphal switch and regulated by Efg1p; strong oxidative stress induced; shows colony morphology-related gene regulation by Ssn6p | 1.62 |
| orf19.5622 | GLC3 | Protein described as similar to 1,4-glucanbranching enzyme; fluconazole-induced; shows colony morphology-related gene regulation by Ssn6p | 1.80 |
| orf19.4833 | MLS1 | Malate synthase; enzyme of the glyoxylate cycle; peroxisomal; no mammalian homolog; transcription isinduced upon phagocytosis by macrophage; regulated upon white-opaque switching; biofilm-repressed; strong oxidative stress induced | 2.36 |
| orf19.6844 | ICL1 | Isocitrate lyase; enzyme of glyoxylate cycle; required for wild-type virulence in murine systemic infection; no mammalian homolog; gene induced upon phagocytosis by macrophage; farnesol regulated; Pex5p-dependent peroxisomal localization | 1.75 |
| orf19.6178 | FBP1 | Fructose-1,6-bisphosphatase, a key enzyme of gluconeogenesis; involved in the carbohydrate metabolism; regulated by Efg1p, Ssn6p; upregulated upon phagocytosis, effecting switch from glycolysis to gluconeogenesis in macrophage | 1.61 |
| orf19.6116 | GLK4 | Protein described as a glucokinase; decreased expression in hyphae compared to yeast-form cells | 1.89 |
| orf19.4543 | UGA2 | Protein not essential for viability; similar to S. cerevisiae Uga2p, which is succinate semialdehyde dehydrogenase with a role in glutamate catabolism; transcription regulated by Mig1p, Tup1p, Gcn4p | 0.94 |
| orf19.7306 | orf19.7306 | Protein of aldo-keto reductase family; increased transcription is associated with MDR1 overexpression, benomyl or long-term fluconazole treatment; overexpression does not affect drug or oxidative stress sensitivity | 1.15 |
| *Protein Metabolism* | | | |
| orf19.4956 | RPN1 | Predicted ORF in Assemblies 19, 20 and 21;regulated by Gcn2p and Gcn4p | 0.84 |
| orf19.1628 | LAP41 | Protein not essential for viability; similar to S. cerevisiae Lap4p, which is a vacuolar amino peptidase | 0.95 |
| orf19.2340 | CDC48 | Protein described as microsomal ATPase; regulated by Gcn2p and Gcn4p; induced in response to amino acid starvation (3-aminotriazole treatment);macrophage/pseudohyphal-repressed | 0.81 |
| orf19.441 | RPT1 | Predicted ORF in Assemblies 19, 20 and 21;regulated by Gcn2p and Gcn4p | 0.93 |
| orf19.3554 | AAT1 | Protein described as aspartate aminotransferase; soluble protein in hyphae; macrophage-induced protein; alkaline upregulated; amphotericin B repressed; gene isused for strain identification by multi locus sequence typing | 2.27 |
| orf19.6287 | AAT21 | Predicted ORF in Assemblies 19, 20 and 21; Gcn4p-regulated | 0.72 |
| orf19.5610 | ARG3 | Alkaline downregulated; Gcn4p-regulated | 0.75 |
| orf19.2551 | MET6 | Essential 5-methyltetrahydropteroyltriglutamate-homocysteine methyl transferase (cobalamin-independent methionine synthase); antigenic during murine or human systemic infection; heat shock, estrogen, possibly biofilm, and GCN-induced | 1.05 |
| orf19.6040 | SNF7 | Protein involved in echinocandin, azole sensitivity; role in proteolytic activation of Rim101p;separable roles in RIM101 pathway and in transport from MVB to vacuole; similar to S. cerevisiae Snf7p component of ESCRT III complex | 0.88 |
| orf19.5623 | ARP4 | Predicted ORF in Assemblies 19, 20 and 21 | 1.52 |
| orf19.2192 | GDH2 | Putative NAD-specific glutamate dehydrogenase; fungal-specific (no human or murine homolog);transcription is regulated by Nrg1p, Mig1p, Tup1p, andGcn4p | 1.88 |
| orf19.1153 | GDA1 | Protein described as glutamate decarboxylase; macrophage-downregulated gene; alkaline downregulated; amphotericin B induced; transcriptionally activated byMnl1p under weak acid stress | 1.50 |
| orf19.6993 | GAP2 | Protein similar to amino acid permeases; ketoconazole, flucytosine repressed; induced by histidine,and induction requires Ssy1p; regulated by Nrg1p, Tup1p;shows colony morphology-related gene regulation by Ssn6p | 1.37 |
| *Lipid Metabolism* | | | |
| orf19.1288 | FOX2 | Predicted 3-hydroxyacyl-CoA epimerase, required for fatty acid beta-oxidation; upregulated upon phagocytosis; transcription is regulated by Mig1p;expression is regulated upon white-opaque switching | 1.42 |
| orf19.272 | FAA21 | Predicted acyl CoA synthetase; upregulated upon phagocytosis; transcription is regulated by Nrg1p andMig1p | 1.44 |
| orf19.3198 | OBPA | Similar to oxysterol binding protein; gene islocated within the MTLa mating-type-like locus; Plc1p-regulated | 1.33 |
| orf19.767 | ERG3 | C-5 sterol desaturase; introduces C-5(6) double bond into episterol in ergosterol biosynthesis; clinically-isolated homozygous null mutants show azole resistance, defects in hyphal growth and virulence; | 1.05 |
| *Stress* | | | |
| orf19.4082 | DDR48 | Immunogenic stress-associated protein; regulated by filamentous growth pathways; induced by benomyl, caspofungin, or in azole-resistant strain; Hog1p, alkaline downregulated; similar to S. cerevisiae Ddr48p(ATP/GTPase, role in DNA repair) | 3.17 |
| orf19.424 | TRP99 | Protein described as thioredoxin peroxidase/alkyl hydroperoxide reductase; transcriptionally regulated by iron; expression greater in low iron; regulated by Gcn4p; induced in response to aminoacid starvation (3-aminotriazole treatment) | 1.23 |
| orf19.882 | HSP78 | Protein described as a heat-shock protein; transcriptionally regulated by macrophage response; transcription is regulated by Nrg1p, Mig1p, Gcn2p, Gcn4p,Mnl1p; heavy metal (cadmium) stress-induced | 1.84 |
| orf19.4526 | HSP30 | Protein described as similar to heat shock protein; fluconazole-downregulated; amphotericin B induced | 2.40 |
| orf19.4980 | HSP70 | Putative chaperone of Hsp70 family; role insensitivity to beta-defensin peptides; heat-shock, amphotericin B, Cd, ketoconazole-induced; farnesol-downregulated in biofilm; surface localized in yeast-form and hyphal cells; antigenic in host | 2.10 |
| orf19.4147 | GLR1 | Glutathione reductase; transcriptionally regulated by Cap1p; upregulated in the presence of human neutrophils; oxidative stress-induced via Cap1p;overexpression correlates with multidrug resistance phenotype in a mutant lacking CAP1 | 0.75 |
| orf19.3150 | GRE2 | Protein described as a reductase; transcriptionis regulated by Nrg1p and Tup1p; benomyl-induced; hyphal-induced; macrophage/pseudohyphal-repressed; expression greater in low iron; reported to be involved in osmotic stress response | 1.35 |
| orf19.2613 | ECM4 | Protein similar to S. cerevisiae Ecm4p;transcription is regulated by Nrg1p and Tup1p; induced in core stress response or in cyr1 or ras1 homozygous null mutant (yeast-form or hyphal cells); transposon mutation affects filamentous growth | 1.34 |
| orf19.1149 | MRF1 | Protein similar to mitochondrial respiratory proteins; increased transcription is observed upon benomyl treatment; induced by nitric oxide; induced in core stress response; oxidative stress-induced via Cap1p | 0.89 |
| orf19.842 | ASR3 | Gene regulated by cAMP and by osmotic stress; greater mRNA abundance observed in a cyr1 or ras1homozygous null mutant than in wild type; possibly spurious ORF (Annotation Working Group prediction) | 2.66 |
| orf19.7284 | ASR2 | Gene regulated by cAMP and by osmotic stress; greater mRNA abundance observed in a cyr1 or ras1homozygous null mutant than in wild type | 2.04 |
| orf19.2344 | ASR1 | Protein described as similar to heat shock proteins; transcription regulated by cAMP, osmotic stress, ciclopirox olamine, ketoconazole; negatively regulated byCyr1p, Ras1p; shows colony morphology-related gene regulation by Ssn6p | 2.32 |
| orf19.3612 | PST2 | Putative NADH: quinone oxidoreductase; similar to1,4-benzoquinone reductase; immunogenic in mouse; increased transcription on benomyl treatment; oxidative stress-induced via Cap1p; fungal-specific (no human or murine homolog) | 1.04 |
| orf 19.251 | orf 19.251 | Member of ThiJ/PfpI protein family; antigenic(Cand a 3 allergen); binds human immunoglobulin E; 2 N-glycosylation motifs; alkaline, fluconazole, Hog1p-downregulated; induced in core stress response or by oxidative stress (via Cap1p); possibly benomyl induced | 1.89 |
| *Iron Homeostasis* | | | |
| orf19.4720 | CTR2 | Predicted ORF in Assemblies 19, 20 and 21;induced by nitric oxide | 1.19 |
| orf19.1415 | FRE10 | Major cell-surface ferric reductase under low-iron conditions; 7 transmembrane regions and a secretion signal predicted; repressed by Tup1p, Rim101p, Ssn6p,Hog1p, caspofungin; ciclopirox olamine induced; not required for filamentous growth | 1.16 |
| orf19.7219 | FTR1 | High-affinity iron permease (ferric citrate, ferrioxamines E or B, transferrin); required for mouse virulence, low-iron growth; iron, amphotericin B, caspofungin, ciclopirox, Hog1p regulated; complements S.cerevisiae ftr1 iron transport  j | 3.00 |
| orf19.2179 | SIT1 | Transporter of ferrichrome siderophores, but not ferrioxamine B; required for wild-type invasion of human epithelial cells in vitro, but not for wild-type systemic virulence in mouse; transcription regulated by iron,Sfu1p, Rfg1p, Tup1p | 1.37 |
| orf19.4215 | FET34 | Protein similar to multicopper ferroxidase; expression greater in low iron and reduced in a fluconazole-resistant isolate; downregulated by Sfu1p,Hog1p; alkaline upregulated by Rim101p; ciclopirox olamine induced | 2.42 |
| orf19.6073 | HMX1 | Heme oxygenase; acts in utilization of hemin iron; gene transcriptionally activated by heat, low iron,or hemin; negatively regulated by Efg1p; expression greater in low iron; upregulated by Rim101p at pH 8 | 2.85 |
| orf19.3940.1 | CUP1 | Metallothionein, involved in copper resistance; transcription is induced by copper | 2.12 |
| orf19.5634 | FRP1 | Predicted ferric reductase; gene alkaline-induced directly by Rim101p; iron-chelation-induced by CCAAT-binding factor (with Hap43p); fluconazole-downregulated; ciclopirox olamine induced; colony morphology-related gene regulation by Ssn6p | 2.42 |
| orf19.4802 | FTH1 | Protein not essential for viability; similar toS. cerevisiae Fth1p (putative high affinity iron transporter for intravacuolar stores of iron); repressed by Sfu1p, amphotericin B, caspofungin; induced by alkaline pH, ciclopirox olamine | 1.73 |
| *Host Pathogen Interaction and Virulence* | | | |
| orf19.4555 | ALS4 | ALS family protein; role in adhesion and wild-type germ tube induction; growth and temperature regulated; expressed during infection of human buccal epithelial cells; down-regulated upon vaginal contact; putative GPI-anchored | 3.13 |
| orf19.1097 | ALS2 | ALS family protein; role in adhesion, biofilmformation, germ tube induction; expressed at infection of human buccal epithelial cells; putative GPI-anchor; induced by ketoconazole, low iron and at cell wall regeneration; regulated by Sfu1p | 3.44 |
| orf19.7622 | SPT3 | Functional homolog of S. cerevisiae Spt3p;required for virulence in mouse systemic infection; homozygous null mutant is hyperfilamentous | 1.09 |
| orf19.2480.1 | AUT7 | ORF Predicted by Annotation Working Group; macrophage/pseudohyphal-repressed; intron in 5'-UTR | 1.34 |
| orf19.7114 | CSA1 | Surface antigen on elongating hyphae and buds; no obvious hyphal defects in mutant; strain variation in number of repeat domains; upregulated in filaments; alkaline upregulated by Rim101p; ciclopirox induced; Efg1p- and Cph1p-regulated | 0.83 |
| orf19.3959 | SSD1 | Protein with role in resistance to host antimicrobial peptides; virulence role in murine infection; functional homolog of S. cerevisiae Ssd1p,which suppresses various mutant phenotypes; constitutively expressed and not cell-cycle regulated | 1.25 |
| orf19.4899 | GCA1 | Predicted extracellular or plasma membrane-associated glucoamylase; possible adhesin; gene is transcribed during rat oral infection; transcription is regulated by carbohydrates, pH, induced by galactose; 15 N-glycosylation sites predicted | 2.83 |
| *DNA synthesis, damage and Repair* | | | |
| orf19.4275 | RAD9 | Protein involved in regulation of DNA-damage-induced filamentous growth; putative component of DNA damage checkpoint; ortholog of <i>S. cerevisiae</i> Rad9p;transcription is induced in response to alpha pheromone in SpiderM medium | 1.02 |
| orf19.1331 | HSM3 | Protein not essential for viability; similar to S. cerevisiae Hsm3p, which may be involved in DNA mismatch repair | 2.50 |
| orf19.5061 | ADE5,7 | Enzyme of adenine biosynthesis; interacts withVps34p; required for hyphal growth and virulence; flucytosine induced; not induced during GCN response, in contrast to the S. cerevisiae ortholog | 0.83 |
| *Transport Activity* | | | |
| orf19.2370 | DSL1 | Protein similar to S. cerevisiae Dsl1p, which isa member of the t-SNARE complex of the endoplasmic reticulum | 1.02 |
| orf19.1042 | POR1 | Protein described as similar to mitochondrialouter membrane porin; in detergent-resistant membranefraction (possible lipid raft component); antigenic inhuman, mouse; flucytosine, macrophage induced; fluconazoleor caspofungin repressed | 0.93 |
| orf19.459 | ADP1 | Putative PDR-subfamily ABC transporter; similar to WHITE subfamily proteins; gene used for strain identification by multilocus sequence typing | 1.11 |
| orf19.4546 | HOL4 | Protein described as an ion transporter; alkaline upregulated by Rim101p; Plc1p-regulated;caspofungin repressed | 1.24 |
| orf19.5447 | HGT19 | Putative glucose/myo-inositol transporter ofmajor facilitator superfamily; 12 transmembrane segments,extended N terminus; expressed in rich medium, 2% glucose;phagocytosis-induced; C. albicans glucose transporterfamily has 20 members | 1.04 |
| orf19.2584 | OPT9 | Probable pseudogene similar to fragments of OPT1oligopeptide transporter gene; decreased expression in hyphae compared to yeast-form cells; transcriptionally induced upon phagocytosis by macrophage | 1.69 |
| orf19.2942 | DIP5 | Putative permease for dicarboxylic amino acids; transcriptionally induced upon phagocytosis by macrophage;Gcn4p-regulated; upregulated by Rim101p at pH 8 | 0.96 |
| orf19.4003 | TIP20 | Protein interacting with Sec20p, possibly involved in retrograde transport between the Golgi and the endoplasmic reticulum; similar to S. cerevisiae Tip20p | 0.88 |
| orf19.3668 | HGT2 | Putative glucose transporter of the major facilitator superfamily; the C. albicans glucose transporter family comprises 20 members; 12 probable membrane-spanning segments; expressed in rich medium with2% glucose | 2.85 |
| orf19.5640 | PEX5 | Protein of the Pex5p family; required for PTS1-mediated peroxisomal protein import, fatty acid beta-oxidation; similar to S. cerevisiae Pas10p peroxisomaltargeting receptor; macrophage/pseudohypl-repressed | 1.16 |
| *Yeast to Hyphal transition* | | | |
| orf19.3548.1 | WH11 | Cytoplasmic protein expressed specifically in white phase yeast-form cells; expression in opaque cells increases virulence and frequency of opaque-to-whites witching; null mutant shows wild-type switching; similar to S. cerevisiae Hsp12p | 2.20 |
| orf19.1354 | UCF1 | Transcriptionally regulated by iron or by yeast-hyphal switch; expression greater in high iron, decreasedupon yeast-hyphal switch; downregulation correlates with clinical development of fluconazole resistance; Ras1p-regulated | 3.37 |
| orf19.2014 | BCY1 | Regulatory subunit of protein kinase A; required for nuclear localization of Tpk1p; physically interacts with Tpk1p; essential for viability; apoptosis-regulated;bcy1 heterozygous or bcy1 tpk2 mutant has filamentous growth defect | 0.94 |
| orf19.729 | SHE3 | Protein similar to S. cerevisiae She3p;transposon mutation affects filamentous growth | 1.13 |
| orf19.2277 | TPK2 | Catalytic subunit of cAMP-dependent protein kinase (PKA), isoform of Tpk1p; required for wild-type epithelial cell damage and engulfment and oral (not systemic) virulence in mouse; involved in control of morphogenesis and stress response | 2.08 |
| orf19.6645 | HMO1 | High mobility group-like protein; activates pseudohyphal growth when expressed in S. cerevisiae; decreased expression in hyphae compared to yeast-form cells; amphotericin B repressed | 1.25 |
| orf19.2241 | PST1 | Protein described as 1,4-benzoquinone reductase;biofilm induced; hyphal-induced expression, regulated byCyr1p, Ras1p, Efg1p, Nrg1p, Rfg1p, Tup1p | 2.56 |
| orf19.1339 | CPY1 | Carboxypeptidase Y; transcriptionally regulated at yeast-hyphal transition or macrophage response; upregulated in the presence of human neutrophils; regulated by Gcn2p and Gcn4p; putative N-glycosylation | 1.27 |
| orf19.868 | ADAEC | Transcription is specific to white cell type | 0.86 |
| orf19.1084 | CDC39 | Protein similar to S. cerevisiae Cdc39p, which is part of the CCR4-NOT transcription regulatory complex; transposon mutation affects filamentous growth | 0.76 |
| orf19.3934 | CAR1 | Transcription is regulated by Nrg1p, Mig1p, andTup1p; shows colony morphology-related gene regulation bySsn6p; alkaline upregulated | 1.33 |
| *Transcription* | | | |
| orf19.5558 | RBF1 | Transcription factor; glutamine-rich activation domain; binds RPG-box DNA sequences; predominantly nuclear; antigenic during human oral infection; mutation causes accelerated induction of filamentous growth | 1.71 |
| orf19.801 | TBF1 | Essential transcriptional activator that binds to a conserved sequence at ribosomal protein genes and the rDNA locus; acts with Cbf1p at a subset of promoters; biological role is analogous to that of S. cerevisiaeRap1p, not to S.c. Tbf1p | 1.64 |
| orf19.1228 | HAP2 | CCAAT-binding factor regulates low-iron(chelation) induction of FRP1 transcription, and under these conditions CBF comprises Hap43p and probably Hap2p and Hap3p | 0.86 |
| orf19.1453 | SPT5 | Protein similar to S. cerevisiae Spt5p transcription elongation factor; transposon mutation affects filamentous growth | 1.03 |
| orf19.6845 | orf19.6845 | Putative transcription factor with bZIP DNA-binding motif | 1.68 |
| *Drug Response* | | | |
| orf19.2693 | GST2 | Increased transcription is observed upon benomyl treatment transcription and in populations of cells exposed to fluconazole over multiple generations; regulated by Nrg1p, Tup1p; induced by nitric oxide | 1.42 |
| orf19.2839 | CIRT4B | Decreased transcription is observed in an azole-resistant strain that overexpresses CDR1 and CDR2 | 2.18 |
| orf19.1942 | SGE1 | Protein described as a multidrug resistance factor; transcriptionally regulated by iron; expression greater in low iron | 1.08 |
| orf19.1683 | PPH21 | Predicted ORF in Assemblies 19, 20 and 21;caspofungin repressed | 0.83 |
| orf19.778 | PIL1 | Echinocandin-binding protein; localizes to cell surface of hyphae, but not yeast-form cells; biofilm induced; fungal-specific (no human or murine homolog) | 1.19 |
| orf19.7350 | orf19.7350 | Soluble protein in hyphae; fluconazole-induced; mRNA abundance is elevated in a cyr1 or ras1 null mutant and decreased mRNA abundance in an efg1 null mutant; regulated by Nrg1p, Tup1p, Tbf1p, Ssn6p | 1.17 |
| *Miscellaneous* | | | |
| orf19.3311 | IFD3 | Transcription is regulated by Mig1p | 0.83 |
| orf19.2434 | NPL4 | Predicted ORF in Assemblies 19, 20 and 21;regulated by Gcn2p and Gcn4p | 0.84 |
| orf19.4416 | VPS13 | Predicted ORF in Assemblies 19, 20 and 21; gene used for multilocus sequence typing | 1.36 |
| orf19.7242 | NCR1 | Transcription is regulated by Nrg1p and Mig1p | 1.96 |
| orf19.2474 | PRC3 | Transcription is regulated by Nrg1p and Mig1p;regulated by Gcn2p and Gcn4p | 1.20 |
| orf19.5544 | SAC6 | Transcription is regulated by Nrg1p and Mig1p | 1.39 |
| orf19.6972 | SMI1B | Protein of unknown function; transcription is negatively regulated by Rim101p | 1.09 |
| orf19.2242 | PRB1 | Putative endoprotease B; regulated by heat, carbon source (GlcNAc-induced), nitrogen, macrophage response, human neutrophils; putative D200-H232-S389catalytic triad; similar to (but does not replace) S.cerevisiae vacuolar B protease Prb1p | 1.56 |
| orf19.6834.10 | TAR1 | Ortholog of S. cerevisiae Tar1p, encoded within the 25S rRNA gene on the opposite strand; transcription is positively regulated by Tbf1p | 1.19 |
| orf19.6322 | ARD | D-arabitol dehydrogenase, NAD-dependent (ArDH);enzyme of D-arabitol and D-arabinose catabolism; D-arabitol is a marker for active infection in humans; hasconserved YXXXK motif of short-chain alcohol-polyol-sugardehydrogenases | 2.22 |
| orf19.2107.1 | STF2 | Protein involved in ATP biosynthesis; decreased expression in hyphae compared to yeast-form cells; downregulated by Efg1p; transcription is upregulated in clinical isolates from HIV+ patients with oral candidiasis | 2.31 |
| orf19.4979 | KNS1 | Protein not essential for viability; similar to S. cerevisiae Kns1p, which is a protein kinase | 1.82 |
| orf19.6225 | PCL7 | Protein described as cyclin-like, possiblePho85p cyclin; hyphal downregulated; transcriptionally activated by Mnl1p under weak acid stress | 1.04 |
| orf19.3997 | ADH1 | Alcohol dehydrogenase; at surface of yeast-form cells but not hyphae; soluble in hyphae; immunogenic inhuman or mouse; complements S. cerevisiae adh1 adh2 adh3mutation; regulated by growth phase, carbon source; fluconazole-induced | 1.16 |
| orf19.2608 | ADH5 | Putative alcohol dehydrogenase; soluble proteinin hyphae; expression is regulated upon white-opaque switching; fluconazole-induced; antigenic during murine systemic infection; regulated by Nrg1p, Tup1p; macrophage-downregulated protein | 2.7 |
| orf19.5288 | IFE2 | Protein described as an alcohol dehydrogenase; decreased expression in hyphae compared to yeast-formcells; Efg1p-regulated; fluconazole-induced; Hog1p-induced | 1.80 |
| orf19.1237 | ARO9 | Aromatic transaminase of the Ehrlich fusel oil pathway of aromatic alcohol biosynthesis; Rim101p-dependent pH-regulation (alkaline induced) | 1.56 |
| orf19.7310 | orf19.7310 | Protein similar to S. cerevisiae Gin3p;transcription is upregulated in response to treatment with ciclopirox olamine; positively regulated by Sfu1p; Hog1p,fluconazole-downregulated | 2.82 |
| *Cell Wall Integrity and Maintainence* | | | |
| orf19.7586 | CHT3 | Chitinase, major; functional homolog of S.cerevisiae Cts1p; 4 N-glycosylation motifs; possible O-mannosylated region; putative signal peptide; hyphal-repressed; farnesol upregulated in biofilm; regulated byEfg1p, Cyr1p, Ras1p | 0.96 |
| orf19.5636 | RBT5 | GPI-anchored cell wall protein; has CRoW motif, not required for filamentous growth; expression is regulated by Rfg1p, Rim101p, Tbf1p, iron; repressed bySfu1p, Hog1p, Tup1p; induced by serum, alkaline pH, ketoconazole, ciclopirox olamine | 1.99 |
| orf19.2768 | AMS1 | Putative alpha-mannosidase; transcription is regulated by Nrg1p; induced during cell wall regeneration | 2.92 |
| orf19.6302 | PGA39 | Putative GPI-anchored protein of unknown function | 3.99 |
| *Uncharacterized* | | | |
| orf19.1353 | orf19.1353 | Predicted ORF in Assemblies 19, 20 and 21;transcription downregulated upon yeast-hyphal switch;Ras1p-regulated | 3.62 |
| orf 19.5063 | orf19.5063 | Predicted ORF in Assemblies 19, 20 and 21;ciclopirox olamine induced; regulated by Ssn6p; induced bynitric oxide in yhb1 mutant | 3.46 |
| orf19.822 | orf19.822 | Predicted ORF in Assemblies 19, 20 and 21;protein detected in some, not all, biofilm extracts; fluconazole-downregulated; greater mRNA abundance observedin a cyr1 or ras1 homozygous null mutant than in wildtype; transcription is upregulated in response tot | 3.44 |
| orf19.670.2 | orf19.670.2 | ORF Predicted by Annotation Working Group | 2.62 |
| orf19.2515 | orf19.2515 | Predicted ORF in Assemblies 19, 20 and 21 | 2.62 |
| orf19.2959.1 | orf19.2959.1 | ORF Predicted by Annotation Working Group | 2.49 |
| orf 19.338 | orf 19.338 | Predicted ORF in Assemblies 19, 20 and 21; Hog1p-downregulated; shows colony morphology-related gene regulation by Ssn6p | 2.49 |
| orf19.699 | orf19.699 | Predicted ORF in Assemblies 19, 20 and 21 | 2.42 |
| orf19.3047 | orf19.3047 | Predicted ORF in Assemblies 19, 20 and 21 | 2.31 |
| orf19.7445 | orf19.7445 | Predicted ORF in Assemblies 19, 20 and 21 | 2.14 |
| orf19.2701 | orf19.2701 | Predicted ORF in Assemblies 19, 20 and 21;greater mRNA abundance observed in a cyr1 or ras1homozygous null mutant than in wild type | 2.05 |
| orf19.2737 | orf19.2737 | Predicted ORF in Assemblies 19, 20 and 21 | 2.02 |
| orf19.6973 | orf19.6973 | Predicted ORF in Assemblies 19, 20 and 21;regulated by Gcn2p and Gcn4p | 1.97 |
| orf 19.1152 | orf 19.1152 | Predicted ORF in Assemblies 19, 20 and 21;regulated by Gcn2p and Gcn4p; induced in core stress response | 1.85 |
| orf19.3351 | orf19.3351 | Predicted ORF in Assemblies 19, 20 and 21 | 1.84 |
| orf19.2686 | orf19.2686 | Predicted ORF in Assemblies 19, 20 and 21 | 1.82 |
| orf19.4132 | orf19.4132 | Predicted ORF in Assemblies 19, 20 and 21 | 1.79 |
| orf19.6637 | orf19.6637 | Predicted ORF in Assemblies 19, 20 and 21 | 1.76 |
| orf 19.5952 | orf 19.5952 | Predicted ORF in Assemblies 19, 20 and 21;induced by nitric oxide in yhb1 null mutant | 1.72 |
| orf 19.3932 | orf 19.3932 | Predicted ORF in Assemblies 19, 20 and 21;induced in core caspofungin response; increased expression observed in an ssr1 homozygous null mutant; induced bynitric oxide in yhb1 mutant | 1.70 |
| orf19.7196 | orf19.7196 | Protein described as a vacuolar protease; upregulated in the presence of human neutrophils | 1.66 |
| orf 19.3984 | orf 19.3984 | Predicted ORF in Assemblies 19, 20 and 21;induced in core caspofungin response; increased expression observed in an ssr1 homozygous null mutant; induced by nitric oxide in yhb1 mutant | 1.63 |
| orf19.5095 | orf19.5095 | Predicted ORF in Assemblies 19, 20 and 21;caspofungin induced | 1.61 |
| orf19.4834 | orf19.4834 | Predicted ORF in Assemblies 19, 20 and 21 | 1.61 |
| orf19.5450 | orf19.5450 | Predicted ORF in Assemblies 19, 20 and 21 | 1.60 |
| orf19.4795 | orf19.4795 | Predicted ORF in Assemblies 19, 20 and 21 | 1.58 |
| orf19.6588 | orf19.6588 | Predicted ORF in Assemblies 19, 20 and 21 | 1.58 |
| orf19.846 | orf19.846 | Predicted ORF in Assemblies 19, 20 and 21;transcriptionally activated by Mnl1p under weak acid stress | 1.54 |
| orf19.2646 | orf19.2646 | Predicted zinc-finger protein of unknown function, not essential for viability | 1.53 |
| orf19.3458 | orf19.3458 | Predicted ORF in Assemblies 19, 20 and 21 | 1.52 |
| orf19.4873 | orf19.4873 | Predicted ORF in Assemblies 19, 20 and 21;expression is regulated upon white-opaque switching | 1.51 |
| orf19.4614 | orf19.4614 | Predicted ORF in Assemblies 19, 20 and 21 | 1.45 |
| orf19.3342 | orf19.3342 | Predicted ORF in Assemblies 19, 20 and 21 | 1.44 |
| orf19.135 | orf19.135 | Predicted ORF in Assemblies 19, 20 and 21 | 1.43 |
| orf19.4503 | orf19.4503 | Predicted ORF in Assemblies 19, 20 and 21 | 1.41 |
| orf19.1117 | orf19.1117 | Predicted ORF in Assemblies 19, 20 and 21;similar to Candida boidinii formate dehydrogenase | 1.38 |
| orf19.1152 | orf19.1152 | Predicted ORF in Assemblies 19, 20 and 21;regulated by Gcn2p and Gcn4p; induced in core stress response | 1.33 |
| orf19.6804 | orf19.6804 | Predicted ORF in Assemblies 19, 20 and 21 | 1.32 |
| orf19.4246 | orf19.4246 | Predicted ORF in Assemblies 19, 20 and 21;similar to S. cerevisiae Ykr070wp; transposon mutation affects filamentous growth; Hog1p-downregulated; shows colony morphology-related gene regulation by Ssn6p;induced during cell wall regeneration | 1.31 |
| orf19.775 | orf19.775 | Predicted ORF in Assemblies 19, 20 and 21 | 1.28 |
| orf19.4128 | orf19.4128 | Predicted ORF in Assemblies 19, 20 and 21 | 1.24 |
| orf19.2870 | orf19.2870 | Predicted ORF in Assemblies 19, 20 and 21 | 1.22 |
| orf19.2296 | orf19.2296 | Predicted ORF in Assemblies 19, 20 and 21;similar to mucins; ketoconazole-induced; fluconazole-downregulated; greater mRNA abundance observed in a cyr1homozygous null mutant than in wild type; colony morphology-related gene regulation by Ssn6p | 1.21 |
| orf19.3563 | orf19.3563 | Protein not essential for viability | 1.20 |
| orf19.4270 | orf19.4270 | Predicted ORF in Assemblies 19, 20 and 21 | 1.15 |
| orf19.3335 | orf19.3335 | Predicted ORF in Assemblies 19, 20 and 21; shows colony morphology-related gene regulation by Ssn6p;repressed by nitric oxide | 1.14 |
| orf19.4287 | orf19.4287 | Predicted ORF in Assemblies 19, 20 and 21 | 1.13 |
| orf19.2844 | orf19.2844 | Predicted ORF in Assemblies 19, 20 and 21;induced in core caspofungin response | 1.11 |
| orf19.5449 | orf19.5449 | Predicted ORF in Assemblies 19, 20 and 21 | 1.09 |
| orf19.3573 | orf19.3573 | Predicted ORF in Assemblies 19, 20 and 21 | 1.08 |
| orf19.2813 | orf19.2813 | Predicted ORF in Assemblies 19, 20 and 21 | 1.08 |
| orf19.1848.1 | orf19.1848.1 | ORF Predicted by Annotation Working Group | 1.06 |
| orf19.4918 | orf19.4918 | Predicted ORF in Assemblies 19, 20 and 21 | 1.05 |
| orf19.6983 | orf19.6983 | Predicted ORF in Assemblies 19, 20 and 21;repressed by nitric oxide | 1.04 |
| orf19.5022 | orf19.5022 | Predicted ORF in Assemblies 19, 20 and 21 | 1.04 |
| orf19.716 | orf19.716 | Predicted ORF in Assemblies 19, 20 and 21; | 1.00 |
| orf19.4842 | orf19.4842 | Predicted ORF in Assemblies 19, 20 and 21 | 0.99 |
