# Supplementary material for: Calcineurin Signaling and Membrane Lipid Homeostasis Regulates Iron Mediated MultiDrug Resistance Mechanisms in Candida albicans
Source: PLoS One. 2011 Apr 12;6(4):e18684. doi: 10.1371/journal.pone.0018684 (PMC3075269; doi:10.1371/journal.pone.0018684)
Supplement: Table S2 — Genes down regulated in response to iron deprivation. Fold expressions of treated vs control are depicted as mean log2 values where a value of -1.0 represents two fold down regulation. (DOC) [file pone.0018684.s004.doc]

**Table: S2**

| **Systematic Name** | **Standard Name** | **Description** | **Mean Log_2_ Fold Expression** |
| --- | --- | --- | --- |
| *Carbohydrate Metabolism* | | | |
| orf19.6385 | ACO1 | Protein described as aconitase; regulated byGcn4p; induced by amino acid starvation (3-AT treatment)or amphotericin B, phagocytosis; fluconazole-downregulated; expression greater in high iron; antigenicin human or murine infection | -2.83 |
| orf19.837 | GNA1 | Glucosamine-6-phosphate acetyltransferase; enzyme of UDP-GlcNAc biosynthesis; required for viability in absence of GlcNAc supplementation; required for persistent infection and wild-type virulence in mouse systemic infection | -2.65 |
| orf19.2871 | SDH12 | Protein with similarity to S. cerevisiae SDH1,which is a flavoprotein subunit of succinate dehydrogenase; soluble protein in hyphae; macrophage-downregulated protein level; downregulated by Efg1p;repressed by nitric oxide | -2.35 |
| orf19.5791 | IDH2 | Protein described as isocitrate dehydrogenase subunit; transcription is upregulated in response to treatment with ciclopirox olamine; transcriptionally regulated by iron; expression greater in high iron | -1.63 |
| orf19.3733 | IDP2 | Putative isocitrate dehydrogenase; expression is regulated upon white-opaque switching; shows colony morphology-related gene regulation by Ssn6p | -1.63 |
| orf19.5013 | AGM1 | Phosphoacetylglucosamine mutase (N-acetylglucosamine-phosphate mutase); enzyme of UDP-N-acetylglucosamine (UDP-GlcNAc) biosynthesis | -1.24 |
| orf19.3097 | PDA1 | Protein described as similar to alpha chain of pyruvate dehydrogenase; fluconazole-induced | -0.95 |
| *Cell wall Maintanence and integrity* | | | |
| orf19.1390 | PMI1 | Phosphomannose isomerase; cell wall biosynthesis enzyme; drug target; functional homolog of S. cerevisiae and E. coli phosphomannose isomerase; Gcn4p-regulated;repressed by 3-AT; induced on adherence to polystyrene, phagocytosis | -2.08 |
| orf19.2685 | PGA54 | Putative GPI-anchored protein; hyphal induced;Hog1p-downregulated; induced in a cyr1 or efg1 homozygous null mutant; shows colony morphology-related gene regulation by Ssn6p; upregulated in an RHE model | -0.95 |
| orf19.2937 | PMM1 | Phosphomannomutase; enzyme of O- and N-linked mannosylation; inter converts mannose-6-phosphate and mannose-l-phosphate; functional homolog of S. cerevisiaeSec53p; antigenic during murine systemic infection | -0.91 |
| orf19.7089 | PMR1 | Putative secretory pathway P-type Ca2+/Mn2+-ATPase, required for protein glycosylation and cell wall maintenance; putative ortholog of S. cerevisiae PMR1 | -1.55 |
| orf19.5760 | IHD1 | Putative GPI-anchored protein of unknown function; alkaline upregulated; greater transcription in hyphal form than yeast form; regulated by Nrg1p, Rfg1p,Tup1p; regulated by Tsa1p, Tsa1Bp in minimal media at 37;not essential for viability | -1.37 |
| orf19.4064 | GPI7 | Protein involved in attachment of GPI-linked proteins to cell wall; member of major facilitator superfamily; phosphodiesterase/nucleotide pyrophosphatase domain; similar to S. cerevisiae Gpi7p | -1.39 |
| orf19.832 | GPI13 | Protein of major facilitator superfamily; has phosphodiesterase/nucleotide pyrophosphatase domain; similar to S. cerevisiae Gpi13p, which acts in GPI anchor biosynthesis | -0.87 |
| *Protein Metabolism* | | | |
| orf19.2546 | TRP2 | Predicted ORF in Assemblies 19, 20 and 21;regulated by Gcn2p and Gcn4p | -0.92 |
| orf19.198 | ASN1 | Protein described as asparagine synthetase; soluble protein in hyphae; regulated by Rim101p; decreased expression at pH 4 compared to pH 8 | -1.64 |
| orf19.4060 | ARO4 | 3-Deoxy-D-arabinoheptulosonate-7-phosphatesynthase; enzyme of aromatic amino acid biosynthesis; GCN-regulated; feedback-inhibited by tyrosine when produced in S. cerevisiae; fungal-specific; Aro3p and Aro4p catalyze the same reaction | -1.08 |
| orf19.1517 | ARO3 | 3-Deoxy-D-arabinoheptulosonate-7-phosphatesynthase; enzyme of aromatic amino acid biosynthesis; GCN-regulated; feedback-inhibited by phenylalanine when heterologously produced in S. cerevisiae; Aro3p and Aro4pcatalyze the same reaction | -2.04 |
| orf19.2098 | ARO8 | Aromatic transaminase of the Ehrlich fusel oil pathway of aromatic alcohol biosynthesis; Rim101p-independent pH-regulation (alkaline induced); protein abundance is affected by URA3 expression in the CAI-4strain background; Gcn4p-regulated | -1.33 |
| orf19.5263 | SER33 | Predicted enzyme of amino acid biosynthesis;Gcn4p-regulated; upregulated in biofilm; S. cerevisiae ortholog is Gcn4p regulated | -1.31 |
| orf19.5094 | BUL1 | Protein not essential for viability;macrophage/pseudohyphal-induced; similar to S. cerevisiaeBul1p, which may be involved in selection of substrates for ubiquitination | -1.1 |
| orf19.814 | SSY1 | Amino acid sensor, required for wild-type hyphal growth on solid serum or Lees media, but not for hyphal growth under all conditions; similar to S. cerevisiaeSsy1p aminoacid sensor and to aminoacid permeases; 12 predicted membrane spanning regions | -1.01 |
| orf19.5480 | ILV1 | Protein described as threonine dehydratase;regulated by Gcn4p and Gcn2p; induced in response to aminoacid starvation (3-aminotriazole treatment) | -0.92 |
| orf19.6779 | PRO2 | Predicted ORF in Assemblies 19, 20 and 21;regulated by Gcn2p and Gcn4p | -1.00 |
| orf19.4650 | ILV6 | Putative regulatory subunit of acetolacetate synthase ; fungal-specific (no human or murine homolog);alkaline upregulated; regulated by Gcn2p and Gcn4p | -0.81 |
| orf19.1358 | GCN4 | Transcriptional activator of general amino acid control response; required for Efg1p-dependentpseudohyphal filament induction by amino acid starvation but not by serum; upregulated in the presence of human whole blood or PMN cells | -2.1 |
| orf19.6257 | GLT1 | Alkaline downregulated | -1.82 |
| orf19.56 | ARG2 | Putative enzyme of arginine biosynthesis; transcription of genes of arginine biosynthesis pathway, except for ARG2, is induced upon phagocytosis by macrophage | -0.99 |
| orf19.4026 | HIS1 | ATP phosphoribosyl transferase; enzyme of histidine biosynthesis; fungal-specific (no human, murine homolog); upregulated in biofilm; acid upregulated/alkaline downregulated by Rim101p; regulated by Gcn2p, Gcn4p; strain CA9 is a his1 mutant | -1.59 |
| orf19.3099 | TRP4 | Predicted enzyme of amino acid biosynthesis; upregulated in biofilm; regulated by Gcn2p and Gcn4p; S.cerevisiae ortholog is Gcn4p regulated | -0.88 |
| orf19.4669 | AAT22 | Protein not essential for viability; similar to S. cerevisiae Aat2p, which is an aspartate aminotransferase involved in nitrogen metabolism | -1.11 |
| orf19.269 | SES1 | Seryl-tRNA synthetase; charges the tRNA that recognizes the CUG codon, which typically specifies Leu, but specifies Ser in C. albicans; complements S.cerevisiae ses1 mutant viability; soluble protein in hyphae; macrophage-regulated | -0.97 |
| orf19.2960 | FRS2 | Putative tRNA-Phe synthetase; genes encoding ribosomal subunits, translation factors, and tRNAsynthetases are downregulated upon phagocytosis by murine macrophage | -1.04 |
| orf19.6702 | DED81 | Putative tRNA-Asn synthetase; genes encoding ribosomal subunits, translation factors, and tRNAsynthetases are downregulated upon phagocytosis by murine macrophage | -1.53 |
| orf19.2560 | CDC60 | Cytosolic leucyl tRNA synthetase; has conserved amino acid and ATP binding class I signature, tRNA binding, and proofreading motifs; likely essential for growth, based on in sertional mutagenesis strategy; similar to S. cerevisiae Cdc60p | -1.08 |
| orf19.5746 | ALA1 | Alanyl-tRNA synthetase; translational regulation generates cytoplasmic and mitochondrial forms; regulatedby Gcn4p; repressed upon amino acid starvation (3-AT);translation-related genes downregulated upon phagocytosis by murine macrophage | -1.39 |
| orf19.6749 | KRS1 | Putative tRNA-Lys synthetase; genes encoding ribosomal subunits, translation factors, and tRNA synthetases are downregulated upon phagocytosis by murine macrophage | -1.43 |
| orf19.1295 | VAS1 | Putative tRNA-Val synthetase; genes encoding ribosomal subunits, translation factors, and tRNA synthetases are downregulated upon phagocytosis by murinem acrophage | -1.37 |
| orf19.437 | GRS1 | Putative tRNA-Gly synthetase; genes encoding ribosomal subunits, translation factors, and tRNA synthetases are downregulated upon phagocytosis by murine macrophage | -0.79 |
| orf19.5226 | WRS1 | Putative tRNA-Trp synthetase; genes encoding ribosomal subunits, translation factors, and tRNA synthetases are downregulated upon phagocytosis by murine macrophage | -0.81 |
| orf19.3291 | HMT1 | Major type I protein arginine methyl transferases(PRMT) involved in asymmetric dimethylation of arginine residues; involved in nuclear export of Npl3p | -1.70 |
| *Sulfur Containing amino acids* | | | |
| orf19.6402 | CYS3 | Predicted enzyme of sulfur amino acid biosynthesis; upregulated in biofilm; alkalineupregulated; amphotericin B induced; possibly adherence-induced; induced by heavy metal (cadmium) stress,oxidative stress (via Cap1p); Hog1p regulated | -1.43 |
| orf19.923 | THR1 | Putative homoserine kinase; fungal-specific (no human or murine homolog); transcription is regulated byTup1p; amphotericin B repressed; regulated by Gcn2p andGcn4p | -1.06 |
| orf19.4536 | CYS4 | Predicted enzyme of sulfur amino acidbiosynthesis; antigenic in mouse; upregulated in biofilm;alkaline upregulated; macrophage/pseudohyphal-induced | -0.88 |
| orf19.5025 | MET3 | Putative ATP sulfurlyase of sulfate assimilation; repressed by Met or Cys, Sfu1p, or in fluconazole-resistant isolate; strongly induced on biofilm formation, even in presence of Met and Cys; Hog1p-,caspofungin-, possibly adherence-induced | -2.09 |
| orf19.946 | MET14 | Predicted ORF in Assemblies 19, 20 and 21;predicted role in sulfur metabolism; induced upon biofilm formation; possibly adherence-induced | -2.27 |
| orf19.4076 | MET10 | Predicted enzyme of sulfur amino acid metabolism; biofilm-induced expression; upregulated in the presence of human whole blood or polymorphonuclear (PMN)cells; Hog1p-induced; possibly adherence-induced | -1.47 |
| orf19.2028 | MXR1 | Protein described as a methionine sulfoxide reductase; flucytosine induced; Plc1p-regulated;upregulated in the presence of human neutrophils; macrophage regulated (gene induced, protein decreased);possibly adherence-induced | -0.75 |
| orf19.4099 | ECM17 | Predicted enzyme of sulfur amino acid biosynthesis; putative role in regulation of cell wall biogenesis; upregulated in biofilm; possibly adherence-induced; regulated by Tsa1p, Tsa1Bp under H2O2 stress;Gcn4p-regulated; Tbf1p-activated | -1.37 |
| orf19.7152 | orf19.7152 | Protein similar to Aspergillus CYSK O-acetylserine sulfhydrylase, suggesting that C. albicans uses an O-acetyl-serine (OAS) pathway of sulfur assimilation; upregulated in biofilm | -0.81 |
| *Lipid Metabolism* | | | |
| orf19.5949 | FAS2 | Alpha subunit of fatty-acid synthase; required for virulence in mouse systemic infection and rat oropharyngeal infection models; regulated by Efg1p;fluconazole-induced; amphotericin B repressed | -1.08 |
| orf19.979 | FAS1 | Beta subunit of fatty-acid synthase; multifunctional enzyme; fluconazole-induced; amphotericin B, caspofungin repressed; macrophage/pseudohyphal-induced; fungal-specific (no human or murine homolog) | -1.02 |
| orf19.406 | ERG1 | Squalene epoxidase, catalyzes epoxidation of squalene to 2,3(S)-oxidosqualene in the ergosterol biosynthetic pathway; essential; target of allylamine antifungal drugs; uses NADH as a reducing cofactor, while S. cerevisiae Erg1p uses NADPH | -1.45 |
| orf19.6026 | ERG2 | C-8 sterol isomerase; enzyme of ergosterolbiosynthesis pathway; converts fecosterol to episterol; mutant is hypersensitive to multiple drugs; ketoconazole-induced | -1.61 |
| orf19.922 | ERG11 | Lanosterol 14-alpha-demethylase, member of cytochrome P450 family that functions in ergosterol biosynthesis; target of azole antifungals; may contributeto drug resistance; azole- or biofilm-induced; subject to hypoxic regulation | -1.15 |
| orf19.3732 | ERG25 | Putative C-4 methyl sterol oxidase with role inC4-demethylation of ergosterol biosynthesis intermediates, based on similarity to S. cerevisiae Erg25p; fluconazole-induced; upregulated in biofilm and in azole-resistantstrain | -0.91 |
| orf19.6243 | VPS34 | Autophosphorylated phosphatidylinositol 3-kinase, class III, required for wild-type vesicle transport, hyphal growth, adherence to fibroblasts, and virulence in mouse systemic infection; low abundance mRNA, growth-regulated | -1.16 |
| orf19.3822 | SCS7 | Protein described as ceramide hydroxylase; transcription is regulated by Nrg1p; transcriptionally regulated by iron; expression greater in high iron; fluconazole-induced | -1.24 |
| orf19.1945 | AUR1 | Inositol phosphorylceramide (IPC) synthase, catalyzes the key step in the synthesis of sphingolipids; potential target for antifungal drugs; mutations confer resistance to aureobasidin A | -1.22 |
| orf19.118 | FAD2 | Delta-12 fatty acid desaturase, involved in production of linoleic acid, which is a major component of membranes | -0.95 |
| orf19.4817 | RAM2 | Alpha subunit of heterodimeric protein geranylgeranyl transferase type I and farnesyl transferase;a-specific transcription; lovastatin and fluconazole regulated; GGTase I enzyme binds zinc, is Mg-dependent;Cdc42p is GGTase I substrate | -0.93 |
| orf19.689 | PLB1 | Phospholipase B; required for wild-type host cell penetration and for wild-type virulence in mouse model of systemic infection; Hog1p-induced; predicted signal sequence, N-glycosylation, and possible Y phosphorylation site, not GPI anchor | -1.37 |
| *Stress* | | | |
| orf19.5437 | RHR2 | Putative glycerol 3-phosphatase; roles in osmotic tolerance, glycerol accumulation in response to salt; regulated by macrophage, stress response, yeast-hyphal switch, pheromone, GCN4, HOG1, NRG1, TUP1;antigenic in murine systemic infection | -2.59 |
| orf19.7551 | ALO1 | D-Arabinono-1,4-lactone oxidase, involved in biosynthesis of D-erythroascorbic acid, which has a protective role against oxidative damage; required fo rfull virulence in a mouse model of systemic infection | -1.16 |
| orf19.6515 | HSP90 | Chaperone of Hsp90 family; essential; evolutionary role; C-terminus is antigenic, response is immunoprotective; activated by heat shock; regulated by growth phase; Cd, estrogen-induced; localizes to surface of hyphae, but not yeast-form | -1.39 |
| orf19.717 | HSP60 | Protein described as mitochondrial heat shock protein; soluble protein in hyphae; transcription is regulated by Nrg1p; transcriptionally regulated by iron; expression greater in high iron; heavy metal (cadmium)stress-induced | -1.22 |
| orf19.3340 | SOD2 | Mitochondrial manganese-containing superoxide dismutase; role in protection against oxidative stress; active as homotetramer; N-terminal 34 amino acids removed upon import into mitochondria; H2O2-induced via Cap1p;alkaline downregulated | -1.32 |
| orf19.6033 | CMP1 | Catalytic subunit of calcineurin (Ca[2+]-calmodulin-regulated S/T protein phosphatase); required for wild-type mouse systemic virulence and for wild-type resistance to high pH, Na(+), Li(+), Mn(2+), and for fluconazole tolerance | -1.32 |
| orf19.1413 | YFH1 | Frataxin; homolog of S. cerevisiae Yfh1p,mammalian frataxin; may be required for iron storage or delivery; role in oxidative stress resistance; transcription regulated by Nrg1p, repressed if iron absent, induced by macrophage interaction | -1.53 |
| orf19.3707 | YHB1 | Nitric oxide dioxygenase; acts in nitric oxide scavenging/detoxification; role in virulence in mouse; not essential for viability; similar to S. cerevisiae Yhb1p;transcription activated by NO, macrophage interaction; hyphal downregulated | -1.78 |
| orf19.1377 | IPK2 | Predicted ORF in Assemblies 19, 20 and 21;moderately induced at 42 deg | -1.06 |
| orf19.2435 | MSI3 | Protein of the HSP70 family; antigenic; functional homolog of S. cerevisiae Msi3p; physically interacts (by 2-hybrid) with Cgr1p; transcriptionally regulated by iron; expression greater in high iron; farnesol downregulated in biofilm | -0.83 |
| orf19.7654 | CPR6 | Predicted ORF in Assemblies 19, 20 and 21;macrophage/pseudohyphal-repressed; heavy metal (cadmium) stress-induced | -0.90 |
| orf19.691 | GPD2 | Similar to glycerol 3-P dehydrogenases; regulated by Ssn6p (colony morphology-related), Nrg1p,Efg1p; induced upon osmotic and oxidative stress (viaHog1p), cell wall regeneration, macrophage/pseudohyphal growth, core stress response | -1.44 |
| orf19.4290 | TRR1 | Putative thioredoxin reductase; upregulated in presence of human neutrophils; regulated by Tsa1p, Tsa1Bp;induced by nitric oxide; peroxide-induced; oxidative stress-induced via Cap1p; fungal-specific (no human or murine homolog) | -0.85 |
| orf19.5850 | NOC2 | Protein similar to S. cerevisiae Noc2p;transposon mutation affects filamentous growth; down regulated during core stress response | -1.82 |
| orf19.3015 | ARX1 | Predicted ORF in Assemblies 19, 20 and 21;downregulated during core stress response | -1.02 |
| orf19.489 | DAP1 | Protein similar to S. cerevisiae Dap1p, which isa protein related to mammalian membrane-associated progesterone receptors involved in response to DNA damage; induced in core stress response; Hog1p regulated | -1.44 |
| orf19.2183 | KRE30 | YEF3-subfamily ABC family protein, predicted not to be a transporter; down regulated during core stress response | -2.48 |
| orf19.171 | DBP2 | Predicted ORF in Assemblies 19, 20 and 21;flucytosine induced; down regulated in core stress response | -1.36 |
| orf19.7307 | orf19.7307 | Putative oxido reductase; similar to S.cerevisiae Yml125p; possible Kex2p substrate | -1.20 |
| orf19.1253 | orf19.1253 | Predicted ORF in Assemblies 19, 20 and 21;transcriptionally activated by Mnl1p under weak acid stress | -0.93 |
| *Haem Biosynthesis* | | | |
| orf19.1813 | FLC2 | Protein involved in heme uptake; putative FAD transporter, similar to S. cerevisiae Flc2p | -1.02 |
| orf19.1742 | HEM3 | Hydroxymethylbilane synthase (uroporphyrinogen Isynthase); catalyzes conversion of 4-porphobilinogen tohydroxymethylbilane, the third step in the hemebiosynthetic pathway; transcription greater in high iron,CO2; alkaline downregulated | -1.20 |
| orf19.1744 | HEM4 | Protein described as uroporphyrinogen IIIsynthase; transcriptionally regulated by iron; expression greater in high iron or elevated CO2; alkaline downregulated | -1.98 |
| orf19.4747 | HEM14 | Putative protoporphyrinogen oxidase involved in heme biosynthesis; predicted Kex2p substrate; transcription is regulated by iron; mRNA levels are decreased in a yfh1 null mutant | -1.55 |
| orf19.2601 | HEM1 | Protein described as 5-aminolevulinate synthase; transcriptionally regulated by iron; caspofungin repressed; expression greater in high iron; regulated bySsn6p; induced by nitric oxide | -0.84 |
| orf19.4459 | orf19.4459 | Predicted ORF in Assemblies 19, 20 and 21;similar to S. cerevisiae Ynl234wp, which is a putative heme-binding stress-related protein; transposon mutation affects filamentous growth | -1.14 |
| *Host Pathogen Interaction and Virulence* | | | |
| orf19.1065 | SSA2 | Cell-surface protein of the HSP70 family; antigenic; role in sensitivity to beta-defensin peptides; localizes to surface of hyphae, but not yeast-form cells; farnesol-downregulated in biofilm; caspofungin repressed | -1.52 |
| orf19.5691 | CDC11 | Septin; required for wild-type cell and hyphal morphology, agar-invasive growth, full virulence and kidney tissue invasion in mouse, but not for kidney colonization, immunogenicity; phosphorylation is hyphal-and cell-cycle-regulated | -1.10 |
| orf19.7383 | MNN9 | Protein of N-linked outer-chain mannan biosynthesis; mutant has defective cell wall; required for wild-type hyphal growth; mutant is hygromycin B sensitive and vanadate resistant; has N-terminal membrane-spanning segment (positions 18-34) | -1.33 |
| orf19.548 | CDC10 | Septin, required for wild-type cell, hyphal, or chlamydospore morphology; role in virulence and kidney tissue invasion in a mouse model of systemic infection;forms ring at sites of cell division and filaments in mature chlamydospore | -0.90 |
| orf19.4109 | PMT4 | Protein mannosyl transferase required for normal cell wall composition and full virulence in mouse systemic infection; has roles in hyphal growth and drug sensitivity; one of 5 PMT family members; Axl2p is a substrate | -0.82 |
| orf19.526 | NHP2 | Predicted ORF in Assemblies 19, 20 and 21;macrophage/pseudohyphal-induced | -2.11 |
| orf19.5742 | ALS9 | ALS family protein; expressed during infection of human epithelial cells; confers laminin adhesion to S.cerevisiae; highly variable; ALS family includes cell-surface glycoproteins, some with adhesin function; putative GPI-anchor | -1.41 |
| orf19.6091 | RIM8 | Protein involved in the pH response pathway; required for activation of the transcription factorRim101p and for alkaline pH-induced hyphal growth; shows colony morphology-related gene regulation by Ssn6p | -0.81 |
| orf19.1721 | NCE103 | Carbonic anhydrase involved in the conversion of carbon dioxide to bicarbonate; essential for pathogenesis in host niches with limited CO2; induced upon biofilm formation; transcriptionally activated by Mnl1p under weak acid stress | -1.59 |
| *DNA synthesis, damage and Repair* | | | |
| orf19.2873 | TOP2 | DNA topoisomerase II; catalyzes ATP-dependent DNA relaxation and decatenation in vitro; Y842 predicted to be catalytic; functional homolog of S. cerevisiaeTop2p; sensitive to amsacrine or doxorubicin; farnesol-upregulated in biofilm | -0.86 |
| orf19.5873 | POL1 | Protein described as DNA directed DNA polymerase alpha; RNA abundance regulated by tyrosol and cell density | -1.88 |
| orf19.4616 | POL30 | Protein described as proliferating cell nuclear antigen (PCNA); RNA abundance regulated by tyrosol and cell density; transcriptionally induced by interaction with macrophage; flucytosine induced | -2.20 |
| orf19.2267 | RFA2 | Protein described as DNA replication factor A;RNA abundance regulated by tyrosol and cell density | -1.71 |
| orf19.96 | TOP1 | DNA topoisomerase I; required for wild-type growth and for wild-type mouse virulence; sensitive to camptothecin; induced upon adherence to polystyrene | 0.75 |
| orf19.5032 | SIM1 | Protein involved in cell wall maintenance, redundant with Sun41p; described as a DNA replication regulator; produced under vaginal conditions; macrophage-downregulated gene; transcription is negatively regulated by Rim101p, Cyr1p, Ras1p | -0.99 |
| orf19.7661 | HMI1 | ATP-dependent 3' - 5' helicase involved in maintenance of mitochondrial DNA; ortholog of <i>S.cerevisiae</> Hmi1p | -0.76 |
| orf19.3074 | TLO10 | Member of a family of telomere-proximal genes of unknown function | -1.78 |
| orf19.4195.1 | FCA1 | Cytosine deaminase; enzyme of pyrimidine salvage; functional homolog of S. cerevisiae Fcy1p;mutation is associated with resistance to flucytosine (5-FC) in a clinical isolate; hyphal down regulated; gene has intron | -0.86 |
| orf19.2640 | FUR1 | Uracil phosphoribosyltransferase; enzyme of pyrimidine salvage; predicted tetrameric enzyme; mutations are associated with resistance to flucytosine (5-FC) in clinical isolates; macrophage-induced protein; flucytosine induced | -0.92 |
| orf19.18 | IMH3 | Inosine monophosphate (IMP) dehydrogenase;enzyme of GMP biosynthesis; target of mycophenolic acid and mizoribine monophosphate; antigenic during human or murine infection; soluble protein in hyphae; down regulated in core stress response | -2.09 |
| orf19.3870 | ADE13 | Enzyme of adenine biosynthesis; soluble protein in hyphae; not induced during GCN response, in contrast to the S. cerevisiae ortholog; repressed by nitric oxide | -0.93 |
| orf19.5423 | EST3 | Telomerase subunit; required for telomere maintenance in vivo; separable roles in telomerase activity and processivity; not required for catalytic activity in vitro; related to mammalian Tpp1p | -0.91 |
| orf19.2093 | RFA1 | Protein described as DNA replication factor A;RNA abundance regulated by tyrosol and cell density | -0.75 |
| orf19.4311 | YNK1 | Nucleoside diphosphate kinase (NDP kinase); homo-hexameric; soluble protein in hyphae; flucytosine induced; biofilm induced; macrophage-induced protein | -1.82 |
| orf19.3322 | DUT1 | dUTP pyrophosphatase; converts dUTP to dUMP and pyrophosphate; cell-cycle regulated if expressed in S.cerevisiae; upstream MluI and SCB transcription regulatory elements; induced by 17-beta-estradiol, ethynyl estradiol,macrophage | -1.96 |
| orf19.5004 | RAD54 | Protein similar to S. cerevisiae Rad54p, which is a DNA-dependent ATPase involved in DNA repair; induced under hydroxyurea treatment | -0.85 |
| orf19.866 | RAD32 | Protein similar to S. cerevisiae protein with role in nucleotide excision repair; down-regulation associated with azole resistance | -1.03 |
| *Transporters* | | | |
| orf19.918 | CDR11 | Putative transporter of PDR subfamily of ABC family; Gcn4p-regulated; upregulated by Rim101p at pH 8 | -0.83 |
| orf19.1783 | YOR1 | Protein similar to S. cerevisiae Yor1p, which is a plasma membrane transporter of the ATP-binding cassette(ABC) family involved in resistance to aureobasidin A; transcription is specific to white cell type | -1.39 |
| orf19.5759 | SNQ2 | Protein similar to S. cerevisiae Snq2ptransporter; member of PDR subfamily of ABC family; transposon mutation affects filamentation; benomyl-induced transcription; detected at yeast-form cell plasma membrane by mass spec | -1.12 |
| orf19.24 | RTA2 | Putative floppase; required for sphingolipid long chain base release; mediates calcineurin-dependent resistance to azoles; stress-associated protein; Ca2+, calcineurin-regulated; ketoconazole, caspofungin induced; Plc1p-regulated | -1.58 |
| orf19.1357 | FCY21 | High affinity, high capacity, hypoxanthine-adenine-guanine-cytosine/ H+ symporter; purine-cytosine permease of pyrimidine salvage; similar to S. cerevisiaeFcy2p | -1.42 |
| orf19.1232 | VRG4 | GDP-mannose transporter; essential; required for glycosylation, hyphal growth; functional homolog of S.cerevisiae Vrg4p, which imports GDP-mannose from cytoplasmto Golgi for protein and lipid mannosylation; no mammalianhomolog | -1.03 |
| orf19.2069 | SMF3 | Protein described as vacuolar iron transporter; alkaline upregulated; caspofungin repressed; transcriptionally activated by Mnl1p under weak acid stress | -1.40 |
| orf19.7332 | ELF1 | Protein required for wild-type morphology,growth; Walker A and B (ATP/GTP binding) motifs, ABCtransporter consensus; expressed in hyphal, pseudohyphal,and yeast form, upregulated in biofilm; S. pombe homolog is ABC ATPase of mRNA export | -1.22 |
| orf19.2021 | HGT8 | Putative glucose transporter of the major facilitator superfamily; the C. albicans glucose transporter family comprises 20 members; 12 probable membrane-spanning segments; gene has intron; expressed in rich medium with 2% glucose | -2.47 |
| orf19.4118 | CNT | Cation-coupled nucleoside transporter(H(+)/nucleoside symporter); CNT family protein; transports adenosine, uridine, inosine, guanosine; variant alleles encode high- and low-affinity isoforms; S or G at residue 328 affects specificity | -0.93 |
| orf19.655 | PHO84 | Protein similar to high-affinity phosphate transporters; expression is regulated upon white-opaque switching or biofilm formation; Hog1p, ciclopirox olamineor alkaline induced; caspofungin or stress repressed; upregulated in RHE model | -1.60 |
| orf19.5392 | NGT1 | N-acetylglucosamine (GlcNAc)-specifictransporter; role in GlcNAc (but not serum) induced hyphalgrowth; localizes to plasma membrane; induced by GlcNAc,macrophage engulfment; 12 transmembrane, major facilitatorsuperfamily | -1.43 |
| orf19.3749 | IFC3 | Oligopeptide transporter; transcriptionally induced upon phagocytosis by macrophage; induced by BSA or peptides; fluconazole-induced; upregulated by Rim101p at pH 8; fungal-specific (no human or murine homolog) | -2.75 |
| orf19.4885 | MIR1 | Putative mitochondrial phosphate transporter; caspofungin repressed; expression is increased in a fluconazole-resistant isolate; induced upon adherence to polystyrene | -1.44 |
| orf19.6249 | HAK1 | Putative potassium transporter; similar to Schwanniomyces occidentalis Hak1p; amphotericin B induced; transcriptionally induced upon phagocytosis by macrophage | -0.93 |
| orf19.6176 | SEC61 | Essential protein; expected to act in protein translocation from the endoplasmic reticulum: similar to S. cerevisiae Sec61p; chimeric mutant partially functionally complements S. cerevisiae sec61 defects; 10 transmembrane regions predicted | -1.11 |
| orf19.3122 | ARR3 | Protein not essential for viability; similar to S. cerevisiae Arr3p, which is an arsenite transporter of the plasma membrane required for resistance to arsenic compounds; benomyl-induced; Gcn4p-regulated | -1.33 |
| orf19.4779 | orf19.4779 | Putative transporter; slightly similar to theSit1p siderophore transporter; Gcn4p-regulated; fungal-specific (no human or murine homolog); transcriptionally activated by Mnl1p under weak acid stress | -0.95 |
| *Yeast to Hyphal transition* | | | |
| orf19.4890 | CLA4 | S/T kinase of Ste20p family; required for wild-type filamentous growth and for wild-type organ colonization and virulence in mouse systemic infection; role in chlamydospore formation; functional homolog of S.cerevisiae Cla4p | -1.26 |
| orf19.400 | GCF1 | Protein that binds the regulatory region in the HWP1 promoter; contains an HMG box near the C terminus; decreased expression in hyphae compared to yeast-form cells | -1.02 |
| orf19.5232 | CSI2 | Protein essential for growth; transposon mutation affects filamentous growth | -3.23 |
| orf19.4909 | CBK1 | Putative S/T kinase of cell wall integrity pathway; required for hyphal growth; mutant cells show abnormal morphology and aggregation; Mob2p associated; required for wild-type transcriptional regulation of several cell-wall-associated genes | -1.45 |
| orf19.5805 | DLD1 | Transcription is specific to white cell type; shows colony morphology-related gene regulation by Ssn6p | -1.23 |
| orf19.3696 | TOM22 | Predicted ORF in Assemblies 19, 20 and 21; shows colony morphology-related gene regulation by Ssn6p | -1.04 |
| orf19.390 | CDC42 | Rho-type GTPase; required for budding and maintenance of hyphal growth; GGTase I geranylgeranylated; misexpression blocks hyphal growth, causes avirulence in mouse IV infection; shows actin-dependent localization to hyphal tip | -1.08 |
| orf19.3391 | ADK1 | Protein described as adenylate kinase; decreased expression in hyphae compared to yeast-form cells; macrophage-induced protein; adenylate kinase release can be used as a marker for cell lysis | -0.89 |
| orf19.4490.2 | QCR8 | ORF Predicted by Annotation Working Group; shows colony morphology-related gene regulation by Ssn6p;macrophage/pseudohyphal-induced | -1.25 |
| orf19.2707.1 | QCR9 | ORF Predicted by Annotation Working Group; shows colony morphology-related gene regulation by Ssn6p | -1.86 |
| orf19.1617 | orf19.1617 | Protein similar to S. cerevisiae Ydr282cp;transposon mutation affects filamentous growth | -1.73 |
| orf19.6587 | orf19.6587 | Protein similar to S. cerevisiae Ydr152wp;transposon mutation affects filamentous growth | -0.75 |
| *Transcription* | | | |
| orf19.7359 | CRZ1 | Putative transcription factor; similar to S.cerevisiae calcineurin-regulated transcription factorCrz1p; mutant is fluconazole hypersensitive; likely to act downstream of calcineurin; has C2H2 zinc fingers; not required for mouse virulence | -0.75 |
| orf19.488 | MEX67 | Nuclear export protein; has NTF2-like domain; interacts with Mtr2p via the NTF2-like domain | -1.34 |
| orf19.7577 | MSS51 | Putative mRNA maturation factor; fungal-specific(no human or murine homolog) | -1.23 |
| orf19.6851 | CHL4 | Protein described as having role in chromosome segregation; RNA abundance regulated by tyrosol and cell density | -1.89 |
| orf19.3127 | CZF1 | Transcriptional regulator of white-opaque switching frequency; hyphal growth regulator; has C-terminal zinc finger and central Glu-rich region; expression in S. cerevisiae causes dominant-negative inhibition of pheromone response | -0.97 |
| orf19.3300 | ZPR1 | Protein with putative zinc finger; regulated byGcn4p; repressed in response to amino acid starvation (3-aminotriazole treatment); upregulation correlates with clinical development of fluconazole resistance | -0.78 |
| orf19.909 | STP4 | Putative transcription factor with zinc finge rDNA-binding motif; induced in core caspofungin response; shows colony morphology-related gene regulation by Ssn6p;induced by 17-beta-estradiol, ethynyl estradiol | -1.34 |
| *Ribosomal Protein Machinary* | | | |
| orf19.5106 | DIP2 | Protein similar to S. cerevisiae Dip2p, of small ribonucleoprotein complex; transposon mutation affects filamentous growth | -1.42 |
| orf19.2960 | FRS2 | Putative tRNA-Phe synthetase; genes encodingribosomal subunits, translation factors, and tRNAsynthetases are downregulated upon phagocytosis by murinemacrophage | -1.04 |
| orf19.3788.1 | RPL30 | Similar to S. cerevisiae L30 ribosomal subunit; pre-mRNA is alternatively spliced to productive and unproductive transcripts; splicing is temperature-regulated; colony morphology-related gene regulation bySsn6p; regulated by Nrg1p, Tup1p | -1.76 |
| orf19.2992 | RPP1A | Conserved acidic ribosomal protein, likely to be involved in regulation of translation elongation; interacts with Rpp2Bp; one of four similar C. albicans ribosomal proteins (Rpp1Ap, Rpp1Bp, Rpp2Ap, Rpp2Bp) | -0.86 |
| orf19.1700 | RPS7A | Predicted ribosomal protein; genes encodingcytoplasmic ribosomal subunits, translation factors, andtRNA synthetases are downregulated upon phagocytosis by murine macrophage | -1.63 |
| orf19.7217 | RPL4B | Putative ribosomal protein; genes encoding cytoplasmic ribosomal subunits, translation factors, and tRNA synthetases are downregulated upon phagocytosis by murine macrophage | -1.18 |
| orf19.4223 | GCD11 | Protein similar to S. cerevisiae Gcd11p, which is a subunit of translation initiation factor eIF2; likely to be essential for growth, based on an insertional mutagenesis strategy | -2.17 |
| orf19.3599 | TIF4631 | Putative translation initiation factor eIF4G;overexpression causes hyperfilamentation; hyphal- and macrophage-induced; genes encoding some translation factors are downregulated upon phagocytosis by murine macrophage | -1.07 |
| orf19.4261 | TIF5 | Putative translation initiation factor; genes encoding ribosomal subunits, translation factors, and tRNA synthetases are down regulated upon phagocytosis by murine macrophage | -1.70 |
| orf19.4152 | CEF3 | Translation elongation factor 3 (EF-3);antigenic in human; predicted C-term nucleotide-binding active site, poly-Lys region; localizes to surface of yeast form, not hyphae; polystyrene adherence induced; EF-3 is fungal-specific | -1.84 |
| orf19.1601 | RPL3 | Putative ribosomal protein; similar to S.cerevisiae Rpl3p; gene induced by ciclopirox olamine treatment; genes encoding cytoplasmic ribosomal subunitsare downregulated upon phagocytosis by murine macrophage | -1.34 |
| orf19.5225.2 | RPL27A | Predicted ribosomal protein | -1.18 |
| orf19.3465 | RPL10A | Predicted ribosomal protein; genes encoding cytoplasmic ribosomal subunits, translation factors, and tRNA synthetases are down regulated upon phagocytosis by murine macrophage | -1.21 |
| orf19.4023 | MRP2 | Protein similar to S. cerevisiae Mrp2p, which is a component of the small subunit of the mitochondrial ribosome; transposon mutation affects filamentous growth | -1.07 |
| orf19.6312 | RPS3 | Putative ribosomal protein; Hog1p-induced; genes encoding cytoplasmic ribosomal subunits, translation factors, and tRNA synthetases are downregulated upon phagocytosis by murine macrophage | -0.91 |
| orf19.6873 | RPS8A | Putative ribosomal protein; similar to S.cerevisiae Rps8Bp; gene is induced by ciclopirox olaminetreatment; genes encoding cytoplasmic ribosomal subunitsare downregulated upon phagocytosis by murine macrophage;intron in 5'-UTR | -1.93 |
| orf19.3504 | RPL23A | Putative ribosomal protein; genes encoding cytoplasmic ribosomal subunits, translation factors, and tRNA synthetases are downregulated upon phagocytosis by murine macrophage | -0.97 |
| orf19.7236 | TIF35 | Putative translation initiation factor; genes encoding ribosomal subunits, translation factors, and tRNA synthetases are down regulated upon phagocytosis by murine macrophage | -1.98 |
| orf19.6785 | RPS12 | Protein described as acidic ribosomal proteinS12; regulated by Gcn4p, activated by Tbf1p; repressedunder amino acid starvation (3-aminotriazole treatment);protein abundance is affected by URA3 expression in theCAI-4 strain background | -1.01 |
| orf19.6663 | RPS25B | Predicted ribosomal protein; macrophage/pseudohyphal-induced after 16 h; downregulated upon phagocytosis by murine macrophage; transcription is positively regulated by Tbf1p; intron in 5'-UTR | -1.31 |
| orf19.2967 | TIF34 | Putative translation initiation factor; genes encoding ribosomal subunits, translation factors, and tRNA synthetases are down regulated upon phagocytosis by murine macrophage | -1.20 |
| orf19.2310.1 | RPL29 | Ribosomal protein L29; transcription is induced upon germ tube formation; shows colony morphology-related gene regulation by Ssn6p; intron in 5'-UTR | -0.92 |
| orf19.3334 | RPS21B | Predicted ribosomal protein; genes encoding cytoplasmic ribosomal subunits, translation factors, andtRNA synthetases are downregulated upon phagocytosis bymurine macrophage; transcription is positively regulatedby Tbf1p | -2.18 |
| orf19.4336 | RPS5 | Predicted ribosomal protein;macrophage/pseudohyphal-induced after 16 h; genes encoding cytoplasmic ribosomal subunits, translation factors, and tRNA synthetases are downregulated upon phagocytosis by murine macrophage | -0.92 |
| orf19.2232 | RPL11 | Predicted ribosomal protein; genes encoding cytoplasmic ribosomal subunits, translation factors, and tRNA synthetases are downregulated upon phagocytosis by murine macrophage; colony morphology-related gene regulation by Ssn6p | -1.65 |
| orf19.6403.1 | RPP2A | Conserved acidic ribosomal protein, likely involved in regulation of translation elongation;interacts with Rpp1Bp; one of four similar ribosomal proteins (Rpp1Ap, Rpp1Bp, Rpp2Ap, Rpp2Bp); CUG start codon; upstream uORFs; Tbf1p-activated | -0.90 |
| orf19.3350 | MRP20 | Protein described as component of mitochondrial ribosome; decreased expression in hyphae compared to yeast-form cells | -1.40 |
| orf19.5341 | RPS4A | Predicted ribosomal protein; genes encoding cytoplasmic ribosomal subunits, translation factors, and tRNA synthetases are downregulated upon phagocytosis bymurine macrophage; transcription is positively regulatedby Tbf1p | -1.71 |
| orf19.5081 | FUN12 | Functional homolog of S. cerevisiae Fun12p translation initiation factor eIF5B; genes encoding ribosomal subunits, translation factors, and tRNA synthetases are down regulated upon phagocytosis by murine macrophage | -0.98 |
| orf19.5927 | RPS15 | Putative ribosomal protein;macrophage/pseudohyphal-induced after 16 h; genes encoding cytoplasmic ribosomal subunits, translation factors, and tRNA synthetases are downregulated upon phagocytosis by murine macrophage | -1.79 |
| orf19.7382 | CAM1 | Putative translation elongation factor eEF1gamma | -1.61 |
| orf19.6213 | SUI2 | Putative translation initiation factor; genes encoding ribosomal subunits, translation factors, and tRNA synthetases are down regulated upon phagocytosis by murinemacrophage | -1.56 |
| orf19.3334 | RPS21 | Predicted ribosomal protein; genes encoding cytoplasmic ribosomal subunits, translation factors, and tRNA synthetases are down regulated upon phagocytosis by murine macrophage; transcription is positively regulatedby Tbf1p | -1.10 |
| orf19.3423 | TIF3 | Putative translation initiation factor; genes encoding ribosomal subunits, translation factors, and tRNA synthetases are down regulated upon phagocytosis by murine macrophage | -1.62 |
| orf19.6047 | TUF1 | Putative translation elongation factor;macrophage/pseudohyphal-induced; genes encoding ribosomalsubunits, translation factors, and tRNA synthetases aredownregulated upon phagocytosis by murine macrophage | -1.90 |
| orf19.6906 | ASC1 | Protein described as part of 40S ribosomal subunit; similar to G-beta subunits; antigenic in human or murine infection; soluble in hyphae; iron, temperature,Gcn4p regulated; amino acid starvation (3-aminotriazole),caspofungin repressed | -2.89 |
| orf19.6975 | YST1 | Ribosome-associated protein; antigenic in mouse;complements S. cerevisiae yst1 yst2 mutant; similar tolaminin-binding proteins, but does not bind laminin;possible S/T phosphorylation, N-glycosylation,myristoylation, Gcn4p-regulated | -1.33 |
| orf19.2651 | CAM1-1 | Putative translation elongation factor; genes encoding ribosomal subunits, translation factors, and tRNA synthetases are down regulated upon phagocytosis by murine macrophage | -1.08 |
| orf19.3087 | UBI3 | Fusion of ubiquitin with the S34 protein of the small ribosomal subunit; mRNA decreases upon heat shock, appears to be degraded; functional homolog of S.cerevisiae Ubi3p | -1.02 |
| orf19.7161 | SUI3 | Putative translation initiation factor; genes encoding ribosomal subunits, translation factors, and tRNA synthetases are down regulated upon phagocytosis by murine macrophage | -1.23 |
| orf19.7015 | RPPO | Putative ribosomal protein; antigenic in mouse;biofilm induced; cytoplasmic ribosomal subunit,translation factor, and tRNA synthetase genes are down regulated upon phagocytosis by murine macrophage;transcription induced by Tbf1p | -1.53 |
| orf19.840 | RPL21A | Putative ribosomal protein; genes encoding cytoplasmic ribosomal subunits, translation factors, and tRNA synthetases are downregulated upon phagocytosis by murine macrophage | -1.00 |
| orf19.5466 | RPS24 | Predicted ribosomal protein; hyphal downregulated; genes encoding cytoplasmic ribosomal subunits, translation factors, and tRNA synthetases aredownregulated upon phagocytosis by murine macrophage;transcription activated by Tbf1p | -2.09 |
| orf19.838.1 | RPS9B | Predicted ribosomal protein; genes encoding cytoplasmic ribosomal subunits, translation factors, and tRNA synthetases are downregulated upon phagocytosis by murine macrophage; possibly transcriptionally regulatedupon hyphal formation | -2.43 |
| orf19.7019 | YML6 | Protein described as a mitochondrial ribosomal protein; induced upon adherence to polystyrene | -2.21 |
| orf19.5982 | RPL18 | Predicted ribosomal protein; Plc1p-regulated,Tbf1p-activated; genes encoding cytoplasmic ribosomal subunits, translation factors, and tRNA synthetases aredownregulated upon phagocytosis by murine macrophage | -1.62 |
| orf19.5685 | THS1 | Putative threonyl-tRNA synthetase; transcription regulated by Mig1p and Tup1p; genes encoding ribosomal subunits, translation factors, and tRNA synthetases aredown regulated upon phagocytosis by murine macrophage | -1.26 |
| orf19.5341 | RPS4A | Predicted ribosomal protein; genes encoding cytoplasmic ribosomal subunits, translation factors, and tRNA synthetases are down regulated upon phagocytosis by murine macrophage; transcription is positively regulated by Tbf1p | -1.71 |
| orf19.3541 | ERF1 | Putative translation release factor 1, which interacts with stop codons and promotes release of nascent peptides from ribosomes | -1.60 |
| orf19.437 | GRS1 | Putative tRNA-Gly synthetase; genes encoding ribosomal subunits, translation factors, and tRNA synthetases are down regulated upon phagocytosis by murine macrophage | -0.80 |
| orf19.2709 | ZUO1 | Protein likely to be essential for growth, based on an insertional mutagenesis strategy; similar to S.cerevisiae Zuo1p, which is a cytosolic ribosome-associated chaperone | -0.90 |
| orf19.4931 | orf19.4931 | Putative tRNA-Cys synthetase; genes encoding ribosomal subunits, translation factors, and tRNA synthetases are down regulated upon phagocytosis by murine macrophage; transcription is induced in response to alpha pheromone in Spider medium | -1.44 |
| *Cell Division & Polarized Growth* | | | |
| orf19.2416.1 | MLC1 | Protein with microtubule-dependent localization to the Spitzenkorper, also localizes to cytokinetic ring in hyphae; cell-cycle dependent localization to bud tip (polarisome) and bud neck in yeast and pseudohyphal cells | -1.31 |
| orf19.1960 | CLN3 | G1 cyclin; depletion causes abolished buddingand hyphal growth defects; likely to be essential; farnesol regulated, upregulated in biofilm; functional in S. cerevisiae; similar to S. cerevisiae Cln3p, which is a G1-specific cyclin | -1.14 |
| orf19.4266 | SPR28 | Septin; similar to S. cerevisiae meiotic/sporulation septin; mutant has no obviousphenotype; two introns with non canonical branch site and 5' splice site, respectively; splicing inhibited upon exposure to alpha-factor | -2.41 |
| orf19.6044 | MOB2 | Mob1/phocein domain protein of RAM signaling network of cell wall integrity; role in cell separation, cortical actin polarization; required for hyphal growth; interacts with Cbk1p; functional homolog of S. cerevisiaeMob2p | -1.19 |
| *Drug Response* | | | |
| orf19.7509.2 | LSM6 | ORF Predicted by Annotation Working Group; flucytosine induced | -0.93 |
| orf19.596.1 | NOP10 | ORF Predicted by Annotation Working Group; flucytosine induced | -1.19 |
| orf19.1448 | APT1 | Predicted ORF in Assemblies 19, 20 and 21;flucytosine induced; repressed by nitric oxide | -1.02 |
| orf19.1839 | RPA190 | Predicted ORF in Assemblies 19, 20 and21;flucytosine induced | -2.59 |
| orf19.2555 | URA5 | Protein abundance is affected by URA3 expression in the CAI-4 strain background; flucytosine induced | -1.39 |
| orf19.6298 | SPB4 | Predicted ORF in Assemblies 19, 20 and 21;flucytosine repressed | -1.13 |
| orf19.661 | KRR1 | Decreased transcription is observed upon benomyl treatment or in an azole-resistant strain that overexpresses MDR1; induced upon biofilm formation | -1.19 |
| orf19.5419 | ATP5 | Predicted ORF in Assemblies 19, 20 and 21;caspofungin repressed | -1.18 |
| orf19.3757 | ATP20 | Predicted ORF in Assemblies 19, 20 and 21; shows colony morphology-related gene regulation by Ssn6p;flucytosine induced; caspofungin repressed; macrophage/pseudohyphal-induced | -1.28 |
| orf19.3941 | URA7 | Predicted ORF in Assemblies 19, 20 and 21;flucytosine induced | -1.18 |
| orf19.1839 | RPA190 | Predicted ORF in Assemblies 19, 20 and21;flucytosine induced | -2.59 |
| orf19.1964 | orf19.1964 | Putative protein of unknown function; down regulated upon fluphenazine treatment; upregulated upon benomyl treatment and in an RHE model; regulated byNrg1p, Tup1p | -1.21 |
| *Miscellaneous* | | | |
| orf19.238 | CCP1 | Similar to cytochrome-c peroxidase N terminus; transcription is negatively regulated by Rim101p or alkaline pH; transcription induced by interaction with macrophage or low iron; oxygen-induced activity | -3.38 |
| orf19.7077 | FRE7 | Protein with similarity to ferric reductases; transcription is activated by Mac1p under copper starvation; Plc1p-regulated; transcription is negatively regulated by Rim101p | -1.88 |
| orf19.7112 | FRP2 | Protein described as ferric reductase; alkaline upregulated by Rim101p; fluconazole-down regulated; upregulated in the presence of human neutrophils; possibly adherence-induced | -1.11 |
| orf19.85 | GPX2 | Similar to glutathione peroxidase; expression greater in high iron; alkaline upregulated by Rim101p;transcriptionally induced by alpha factor or interaction with macrophage; regulated by Efg1p; caspofungin repressed | -0.75 |
| orf19.3656 | COX15 | Transcription is regulated by Nrg1p and Tup1p;alkaline down regulated | -1.90 |
| orf19.5280 | MUP1 | Alkaline upregulated by Rim101p | -1.64 |
| orf19.401 | TCP1 | Protein similar to <i>S. cerevisiae</i> Tcp1p;transcription is induced in response to alpha pheromone in Spider M medium | -1.52 |
| orf19.2238 | LTE1 | Protein similar to <i>S. cerevisiae</i> Lte1p;transcription is repressed in response to alpha pheromone in Spide rM medium | -1.07 |
| orf19.3152 | AMO2 | Protein similar to A. niger predicted peroxisomal copper amino oxidase; induced upon biofilm formation | -1.22 |
| orf19.526 | NHP2 | Predicted ORF in Assemblies 19, 20 and 21;macrophage/pseudohyphal-induced | -2.11 |
| orf19.3579 | ATP4 | Predicted ORF in Assemblies 19, 20 and 21;macrophage/pseudohyphal-induced | -1.94 |
| orf19.6511 | LIG1 | tRNA ligase; functional homolog of S. cerevisiaeTrl1p; fungal-specific (no human or murine homolog) | -1.19 |
| orf19.6190 | SRB1 | Essential GDP-mannose pyrophosphorylase; synthesizes GDP-mannose for protein glycosylation; functional homolog of S. cerevisiae Psa1p; at surface of yeast-form, not hyphal, cells; alkaline upregulated; induced on adherence to polystyrene | -0.99 |
| orf19.5005 | OSM2 | Protein described as mitochondrial fumarate reductase; regulated by Ssn6p, Gcn2p, and Gcn4p; Hog1p-downregulated | -1.38 |
| orf19.3481 | orf19.3481 | Putative protein of unknown function, transcription is activated in the presence of elevatedCO2; predicted ORF in Assemblies 19, 20 and 21 | -1.78 |
| orf19.1179 | orf19.1179 | Transcriptionally regulated by iron; expression greater in high iron; possibly subject to Kex2p processing | -1.31 |
| *Oxidative Phosphorylation* | | | |
| orf19.5629 | QCR7 | Protein described as ubiquinol-cytochrome-c reductase, subunit 7 | -1.61 |
| orf19.2644 | QCR2 | Protein described as ubiquinol-cytochrome-creductase; antigenic in human; transcriptionally inducedby interaction with macrophage; repressed by nitric oxide;in detergent-resistant membrane fraction (possible lipidraft component) | -2.41 |
| orf19.3358 | LSC1 | Protein described as succinate-CoA ligase subunit; transcriptionally regulated by iron; expression greater in high iron; fluconazole-induced | -0.74 |
| orf19.4495 | NDH51 | Subunit of nicotinamide adenine dinucleotide dehydrogenase complex I, of the mitochondrial electron transport chain; required for wild-type filamentous growth; alkaline down regulated | -2.46 |
| orf19.5213.2 | COX9 | Putative subunit VIIa of cytochrome c oxidase;f lucytosine induced | -1.1 |
| orf19.1860 | LSC2 | Protein described as beta subunit of succinate-CoA ligase; transcription regulated by Mig1p and Tup1p;transcriptionally regulated by iron; expression greater in high iron; filament induced | -1.46 |
| orf19.637 | SDH2 | Succinate dehydrogenase, Fe-S subunit; localizes to surface of yeast-form cells, but not hyphae; transcriptionally regulated by iron; expression greater in high iron; repressed by nitric oxide; induced during log phase (aerobic growth) | -1.81 |
| orf19.5893 | RIP1 | Protein described as subunit of ubiquinol cytochrome c-reductase; transcriptionally regulated by iron; expression greater in high iron; alkaline down regulated; repressed by nitric oxide | -2.72 |
| orf19.3527 | CYT1 | Protein described as cytochrome c1;transcriptionally regulated by iron; expression greater in high iron; alkaline down regulated | -2.82 |
| orf19.1770 | CYC1 | Cytochrome c; complements defects of an S. cerevisiae cyc1 cyc7 double mutant; transcriptionally regulated by iron; expression greater in high iron; alkaline down regulated; repressed by nitric oxide | -2.28 |
| orf19.5000 | CYB2 | Protein described as precursor protein of cytochrome b2; transcriptionally regulated by iron; expression greater in high iron; alkaline down regulated; shows colony morphology-related gene regulation by Ssn6p | -1.71 |
| orf19.7049 | CYB5 | Cytochrome b(5); not essential for viability; similar to S. cerevisiae Cyb5p; transcriptionally regulated by iron; expression greater in high iron; fluconazole-induced; shows colony morphology-related gene regulation by Ssn6p | -1.48 |
| orf19.6531 | NUC2 | Protein described as NADH-ubiquinone oxidoreductase; identified in detergent-resistant membrane fraction (possible lipid raft component); alkaline down regulated | -1.88 |
| orf19.6794 | FESUR1 | Protein described as ubiquinone reductase; transcriptionally induced by interaction with macrophage; alkaline down regulated; repressed by nitric oxide | -1.46 |
| orf19.7590 | orf19.7590 | Protein described as NADH-ubiquinone oxido reductase; identified in detergent-resistant membrane fraction (possible lipid raft component); predicted N-terminal acetylation; repressed by nitric oxide | -2.15 |
| orf19.1710 | orf19.1710 | Protein described as NADH-ubiquinone oxidoreductase; identified in detergent-resistant membrane fraction (possible lipid raft component); predicted N-terminal acetylation; repressed by nitric oxide | -1.73 |
| orf19.1480 | orf19.1480 | Protein described as succinate dehydrogenase, enzyme of citric acid cycle; down regulated by Efg1p;repressed by nitric oxide | -1.27 |
| *Uncharacterized* | | | |
| orf19.2275 | orf19.2275 | Predicted ORF in Assemblies 19, 20 and 21 | -2.95 |
| orf19.7070 | orf19.7070 | ORF Predicted by Annotation Working Group | -2.84 |
| orf19.3354 | orf19.3354 | Predicted ORF in Assemblies 19, 20 and 21 | -2.79 |
| orf19.3559 | orf19.3559 | Predicted ORF in Assemblies 19, 20 and 21 | -2.73 |
| orf19.2091 | orf19.2091 | Predicted ORF in Assemblies 19, 20 and 21;repressed by nitric oxide | -2.54 |
| orf19.1361 | orf19.1361 | Predicted ORF in Assemblies 19, 20 and 21 | -2.39 |
| orf19.2917 | orf19.2917 | Predicted ORF in Assemblies 19, 20 and 21 | -2.34 |
| orf19.5038 | orf19.5038 | Predicted ORF in Assemblies 19, 20 and 21 | -2.28 |
| orf19.1626 | orf19.1626 | Predicted ORF in Assemblies 19, 20 and 21 | -2.27 |
| orf19.688 | orf19.688 | Predicted ORF in Assemblies 19, 20 and 21 | -2.20 |
| orf19.6220.4 | orf19.6220.4 | ORF Predicted by Annotation Working Group | -2.14 |
| orf19.1872 | orf19.1872 | Predicted ORF in Assemblies 19, 20 and 21;repressed by nitric oxide | -2.07 |
| orf19.6679 | orf19.6679 | Predicted ORF in Assemblies 19, 20 and 21 | -2.03 |
| orf19.2489 | orf19.2489 | Predicted ORF in Assemblies 19, 20 and 21;repressed by nitric oxide | -2.03 |
| orf19.2045 | orf19.2045 | Predicted ORF in Assemblies 19, 20 and 21 | -1.81 |
| orf19.5279 | orf19.5279 | Predicted ORF in Assemblies 19, 20 and 21 | -1.75 |
| orf19.2017 | orf19.2017 | Predicted ORF in Assemblies 19, 20 and 21 | -1.75 |
| orf19.6524 | orf19.6524 | Predicted ORF in Assemblies 19, 20 and 21 | -1.74 |
| orf19.1815 | orf19.1815 | Predicted ORF in Assemblies 19, 20 and 21 | -1.72 |
| orf19.828 | orf19.828 | Predicted ORF in Assemblies 19, 20 and 21 | -1.71 |
| orf19.1873 | orf19.1873 | Predicted ORF in Assemblies 19, 20 and 21;repressed by nitric oxide; possibly spurious ORF(Annotation Working Group prediction) | -1.69 |
| orf19.3547 | orf19.3547 | Predicted ORF in Assemblies 19, 20 and 21 | -1.65 |
| orf19.3556 | orf19.3556 | Predicted ORF in Assemblies 19, 20 and 21 | -1.65 |
| orf19.6177 | orf19.6177 | Predicted ORF in Assemblies 19, 20 and 21;possibly spurious ORF (Annotation Working Group prediction) | -1.64 |
| orf19.2954 | orf19.2954 | Predicted ORF in Assemblies 19, 20 and 21;repressed by nitric oxide; possibly spurious ORF(Annotation Working Group prediction) | -1.64 |
| orf19.6752 | orf19.6752 | Predicted ORF in Assemblies 19, 20 and 21 | -1.62 |
| orf19.7057 | orf19.7057 | Predicted ORF in Assemblies 19, 20 and 21 | -1.62 |
| orf19.2938 | orf19.2938 | Predicted ORF in Assemblies 19, 20 and 21 | -1.58 |
| orf19.3089 | orf19.3089 | Predicted ORF in Assemblies 19, 20 and 21 | -1.58 |
| orf19.3303 | orf19.3303 | Predicted ORF in Assemblies 19, 20 and 21 | -1.57 |
| orf19.1625 | orf19.1625 | Predicted ORF in Assemblies 19, 20 and 21;repressed by nitric oxide | -1.56 |
| orf19.478 | orf19.478 | Predicted ORF in Assemblies 19, 20 and 21 | -1.55 |
| orf19.6934 | orf19.6934 | Predicted ORF in Assemblies 19, 20 and 21 | -1.53 |
| orf19.1802 | orf19.1802 | Predicted ORF in Assemblies 19, 20 and 21 | -1.52 |
| orf19.4283 | orf19.4283 | Predicted ORF in Assemblies 19, 20 and 21 | -1.51 |
| orf19.2201 | orf19.2201 | Predicted ORF in Assemblies 19, 20 and 21 | -1.51 |
| orf19.5077 | orf19.5077 | Predicted ORF in Assemblies 19, 20 and 21;repressed by nitric oxide; possibly spurious ORF(Annotation Working Group prediction) | -1.51 |
| orf19.5684 | orf19.5684 | Predicted ORF in Assemblies 19, 20 and 21 | -1.50 |
| orf19.3938 | orf19.3938 | Predicted ORF in Assemblies 19, 20 and 21 | -1.49 |
| orf19.4375 | orf19.4375 | Predicted ORF in Assemblies 19, 20 and 21 | -1.49 |
| orf19.764 | orf19.764 | Predicted ORF in Assemblies 19, 20 and 21 | -1.47 |
| orf19.3367 | orf19.3367 | Predicted ORF in Assemblies 19, 20 and 21 | -1.47 |
| orf19.4149.1 | orf19.4149.1 | Predicted ORF in Assemblies 19, 20 and 21 | -1.45 |
| orf19.270 | orf19.270 | Predicted ORF in Assemblies 19, 20 and 21 | -1.42 |
| orf19.3022 | orf19.3022 | Predicted ORF in Assemblies 19, 20 and 21 | -1.42 |
| orf19.1833 | orf19.1833 | Predicted ORF in Assemblies 19, 20 and 21 | -1.42 |
| orf19.4665 | orf19.4665 | Predicted ORF in Assemblies 19, 20 and 21 | -1.41 |
| orf19.894 | orf19.894 | Predicted ORF in Assemblies 19, 20 and 21 | -1.40 |
| orf19.6699 | orf19.6699 | Predicted ORF in Assemblies 19, 20 and 21 | -1.39 |
| orf19.3796 | orf19.3796 | Predicted ORF in Assemblies 19, 20 and 21 | -1.37 |
| orf19.5201 | orf19.5201 | Predicted ORF in Assemblies 19, 20 and 21 | -1.36 |
| orf19.6748 | orf19.6748 | Predicted ORF in Assemblies 19, 20 and 21 | -1.35 |
| orf19.7478 | orf19.7478 | Predicted ORF in Assemblies 19, 20 and 21 | -1.34 |
| orf19.1578 | orf19.1578 | Predicted ORF in Assemblies 19, 20 and 21 | -1.33 |
| orf19.5515 | orf19.5515 | Predicted ORF in Assemblies 19, 20 and 21 | -1.32 |
| orf19.3456 | orf19.3456 | Predicted ORF in Assemblies 19, 20 and 21 | -1.32 |
| orf19.319 | orf19.319 | Predicted ORF in Assemblies 19, 20 and 21 | -1.31 |
| orf19.5281 | orf19.5281 | Predicted ORF in Assemblies 19, 20 and 21 | -1.31 |
| orf19.2953 | orf19.2953 | Predicted ORF in Assemblies 19, 20 and 21 | -1.29 |
| orf19.6887 | orf19.6887 | Predicted ORF from Assembly 19; repressed by nitric oxide; removed from Assembly 20; subsequently reinstated in Assembly 21 based on comparative genome analysis | -1.29 |
| orf19.599 | orf19.599 | Predicted ORF in Assemblies 19, 20 and 21 | -1.29 |
| orf19.4468 | orf19.4468 | Predicted ORF in Assemblies 19, 20 and 21 | -1.28 |
| orf19.4796 | orf19.4796 | Predicted ORF in Assemblies 19, 20 and 21 | -1.28 |
| orf19.6586 | orf19.6586 | Predicted ORF in Assemblies 19, 20 and 21;increased transcription is observed upon benomyl treatment or in an azole-resistant strain that overexpresses MDR1;shows colony morphology-related gene regulation by Ssn6p;induced by nitric oxide, 17-beta-estradiol | -1.27 |
| orf19.2019 | orf19.2019 | Predicted ORF in Assemblies 19, 20 and 21 | -1.27 |
| orf19.7237 | orf19.7237 | Predicted ORF in Assemblies 19, 20 and 21 | -1.25 |
| orf19.7485 | orf19.7485 | Predicted ORF in Assemblies 19, 20 and 21 | -1.23 |
| orf19.763 | orf19.763 | Predicted ORF in Assemblies 19, 20 and 21 | -1.22 |
| orf19.7386 | orf19.7386 | Predicted ORF in Assemblies 19, 20 and 21 | -1.22 |
| orf19.6600 | orf19.6600 | Predicted ORF in Assemblies 19, 20 and 21 | -1.22 |
| orf19.4758 | orf19.4758 | Predicted ORF in Assemblies 19, 20 and 21;alkaline down regulated | -1.20 |
| orf19.6230 | orf19.6230 | Predicted ORF in Assemblies 19, 20 and 21 | -1.20 |
| orf19.850 | orf19.850 | Predicted ORF in Assemblies 19, 20 and 21 | -1.19 |
| orf19.3611 | orf19.3611 | Predicted ORF in Assemblies 19, 20 and 21;repressed by nitric oxide | -1.17 |
| orf19.3175 | orf19.3175 | Predicted ORF in Assemblies 19, 20 and 21;alkaline down regulated; repressed by nitric oxide | -1.17 |
| orf19.5985 | orf19.5985 | Predicted ORF in Assemblies 19, 20 and 21 | -1.16 |
| orf19.2214 | orf19.2214 | Predicted ORF in Assemblies 19, 20 and 21 | -1.15 |
| orf19.4837 | orf19.4837 | Predicted ORF in Assemblies 19, 20 and 21 | -1.13 |
| orf19.2643 | orf19.2643 | Predicted ORF in Assemblies 19, 20 and 21 | -1.13 |
| orf19.2439.1 | orf19.2439.1 | ORF Predicted by Annotation Working Group | -1.13 |
| orf19.7041 | orf19.7041 | Predicted ORF in Assemblies 19, 20 and 21 | -1.12 |
| orf19.287 | orf19.287 | Predicted ORF in Assemblies 19, 20 and 21;repressed by nitric oxide; identified in detergent-resistant membrane fraction (possible lipid raft component) | -1.10 |
| orf19.2286 | orf19.2286 | Predicted ORF in Assemblies 19, 20 and 21;ketoconazole-induced | -1.10 |
| orf19.2208 | orf19.2208 | Predicted ORF in Assemblies 19, 20 and 21 | -1.10 |
| orf19.477 | orf19.477 | Predicted ORF in Assemblies 19, 20 and 21 | -1.10 |
| orf19.3348 | orf19.3348 | Predicted ORF in Assemblies 19, 20 and 21 | -1.10 |
| orf19.3124 | orf19.3124 | Predicted ORF in Assemblies 19, 20 and 21 | -1.10 |
| orf19.3290 | orf19.3290 | Predicted ORF in Assemblies 19, 20 and 21;repressed by nitric oxide | -1.09 |
| orf19.3268 | orf19.3268 | Ortholog of S. cerevisiae Tma19p (Ykl065cp) | -1.09 |
| orf19.4831 | orf19.4831 | Predicted ORF in Assemblies 19, 20 and 21 | -1.09 |
| orf19.1697 | orf19.1697 | Predicted ORF in Assemblies 19, 20 and 21 | -1.09 |
| orf19.6585 | orf19.6585 | Predicted ORF in Assemblies 19, 20 and 21 | -1.08 |
| orf19.805 | orf19.805 | Predicted ORF in Assemblies 19, 20 and 21 | -1.08 |
| orf19.7617 | orf19.7617 | Predicted ORF in Assemblies 19, 20 and 21 | -1.08 |
| orf19.5085 | orf19.5085 | Predicted ORF in Assemblies 19, 20 and 21 | -1.07 |
| orf19.6189 | orf19.6189 | Predicted ORF in Assemblies 19, 20 and 21 | -1.07 |
| orf19.163 | orf19.163 | Predicted ORF in Assemblies 19, 20 and 21 | -1.07 |
| orf19.5682 | orf19.5682 | Predicted ORF in Assemblies 19, 20 and 21 | -1.07 |
| orf19.4337 | orf19.4337 | Predicted ORF in Assemblies 19, 20 and 21 | -1.07 |
| orf19.415 | orf19.415 | Predicted ORF in Assemblies 19, 20 and 21 | -1.05 |
| orf19.2376 | orf19.2376 | Predicted ORF in Assemblies 19, 20 and 21 | -1.04 |
| orf19.7109 | orf19.7109 | Predicted ORF in Assemblies 19, 20 and 21 | -1.04 |
| orf19.1767 | orf19.1767 | Predicted ORF in Assemblies 19, 20 and 21 | -1.04 |
|  | orf19.1352 | Predicted ORF in Assemblies 19, 20 and 21 | -1.03 |
| orf19.5352 | orf19.5352 | Protein not essential for viability | -1.03 |
| orf19.7153 | orf19.7153 | Predicted ORF in Assemblies 19, 20 and 21 | -1.02 |
| orf19.3799 | orf19.3799 | Predicted ORF in Assemblies 19, 20 and 21 | -1.01 |
| orf19.6590 | orf19.6590 | Predicted ORF in Assemblies 19, 20 and 21 | -1.01 |
| orf19.6882.1 | orf19.6882.1 | ORF Predicted by Annotation Working Group | -1.01 |
| orf19.7215.3 | orf19.7215.3 | ORF Predicted by Annotation Working Group | -1.00 |
