# Supplementary material for: Calcineurin Signaling and Membrane Lipid Homeostasis Regulates Iron Mediated MultiDrug Resistance Mechanisms in Candida albicans
Source: PLoS One. 2011 Apr 12;6(4):e18684. doi: 10.1371/journal.pone.0018684 (PMC3075269; doi:10.1371/journal.pone.0018684)
Supplement: Table S3 — PGL composition in response to iron deprivation. Values are mean of ± SD (n = 3 for conditions, * depicts P value<0.05). Data is represented as nmoles/mg dry lipid weight (normalized mass spectral signal). (DOC) [file pone.0018684.s005.doc]

**Table: S3**

| **Phosphoglyceride amounts** | | |  |  |  |  |  |
| --- | --- | --- | --- | --- | --- | --- | --- |
|  |  |  | nmol per mg dry wt | | nmol per mg dry wt | |  |
|  |  |  | **WT** |  | **WT+BPS** |  | p-value <0.05 |
| **Sample description** | **Masses** | **Formula** | **ave** | **stdev** | **ave** | **stdev** |  |
| PC 26:1 | 648.5 | C34H67O8PN | 0.039 | 0.004 | 0.236 | 0.336 |  |
| PC 26:0 | 650.5 | C34H69O8PN | 0.093 | 0.009 | 0.346 | 0.487 | * |
| PC 28:1 | 676.5 | C36H71O8PN | 0.198 | 0.049 | 0.747 | 0.955 |  |
| PC 28:0 | 678.5 | C36H73O8PN | 0.261 | 0.012 | 0.302 | 0.264 |  |
| PC 30:2 | 702.5 | C38H73O8PN | 0.475 | 0.061 | 0.576 | 0.132 | * |
| PC 30:1 | 704.5 | C38H75O8PN | 1.073 | 0.122 | 1.233 | 0.239 |  |
| PC 30:0 | 706.5 | C38H77O8PN | 0.252 | 0.018 | 0.269 | 0.279 | * |
| PC 31:2 | 716.5 | C39H75O8PN | 0.132 | 0.019 | 0.204 | 0.198 | * |
| PC 31:1 | 718.5 | C39H77O8PN | 0.226 | 0.016 | 0.300 | 0.198 |  |
| PC 31:0 | 720.6 | C39H79O8PN | 0.029 | 0.004 | 0.097 | 0.151 | * |
| PC 32:2 | 730.5 | C40H77O8PN | 7.872 | 0.635 | 8.013 | 1.442 |  |
| PC 32:1 | 732.6 | C40H79O8PN | 9.155 | 0.650 | 8.971 | 0.419 | * |
| PC 32:0 | 734.6 | C40H81O8PN | 0.144 | 0.038 | 0.076 | 0.132 | * |
| PC 33:2 | 744.6 | C41H79O8PN | 1.390 | 0.133 | 1.145 | 0.120 |  |
| PC 33:1 | 746.6 | C41H81O8PN | 0.965 | 0.116 | 1.166 | 0.337 |  |
| PC 33:0 | 748.6 | C41H83O8PN | 0.019 | 0.003 | 0.108 | 0.168 |  |
| PC 34:4 | 754.5 | C42H77O8PN | 12.815 | 1.212 | 7.040 | 2.656 |  |
| PC 34:3 | 756.6 | C42H79O8PN | 44.571 | 1.990 | 38.286 | 11.580 | * |
| PC 34:2 | 758.6 | C42H81O8PN | 51.077 | 4.760 | 50.145 | 5.063 |  |
| PC 34:1 | 760.6 | C42H83O8PN | 27.463 | 0.447 | 30.998 | 2.990 |  |
| PC 36:6 | 778.5 | C44H77O8PN | 3.813 | 0.073 | 1.598 | 0.807 | * |
| PC 36:5 | 780.6 | C44H79O8PN | 34.136 | 2.889 | 18.925 | 11.919 |  |
| PC 36:4 | 782.6 | C44H81O8PN | 65.971 | 7.314 | 52.096 | 21.380 |  |
| PC 36:3 | 784.6 | C44H83O8PN | 67.634 | 1.867 | 70.324 | 15.312 |  |
| PC 36:2 | 786.6 | C44H85O8PN | 36.407 | 1.866 | 36.388 | 4.134 |  |
| PC 36:1 | 788.6 | C44H87O8PN | 12.446 | 0.707 | 13.007 | 1.081 |  |
| PC 38:6 | 806.6 | C46H81O8PN | 0.212 | 0.029 | 0.240 | 0.172 |  |
| PC 38:5 | 808.6 | C46H83O8PN | 0.803 | 0.097 | 0.492 | 0.093 |  |
| PC 38:4 | 810.6 | C46H85O8PN | 1.313 | 0.113 | 0.888 | 0.122 | * |
| PC 38:3 | 812.6 | C46H87O8PN | 2.056 | 0.294 | 2.239 | 0.740 |  |
| PC 38:2 | 814.6 | C46H89O8PN | 1.041 | 0.114 | 2.963 | 2.856 |  |
| PC 40:5 | 836.6 | C48H87O8PN | 0.045 | 0.003 | 0.103 | 0.119 | * |
| PC 40:4 | 838.6 | C48H89O8PN | 0.067 | 0.029 | 0.130 | 0.111 |  |
| PC 40:3 | 840.6 | C48H91O8PN | 0.150 | 0.087 | 0.244 | 0.127 | * |
| PC 40:2 | 842.7 | C48H93O8PN | 0.143 | 0.026 | 0.263 | 0.171 |  |
| **Total PC** |  |  | **384.486** | **17.263** | **350.159** | **67.460** |  |
| PE 26:1 | 606.4 | C31H61O8PN | 0.009 | 0.002 | 0.052 | 0.070 |  |
| PE 26:0 | 608.4 | C31H63O8PN | 0.046 | 0.027 | 0.148 | 0.191 |  |
| PE 28:1 | 634.4 | C33H65O8PN | 0.071 | 0.015 | 0.249 | 0.316 |  |
| PE 30:2 | 660.5 | C35H67O8PN | 0.128 | 0.019 | 0.107 | 0.013 |  |
| PE 30:1 | 662.5 | C35H69O8PN | 0.410 | 0.131 | 0.328 | 0.042 |  |
| PE 30:0 | 664.5 | C35H71O8PN | 0.030 | 0.005 | 0.036 | 0.027 |  |
| PE 31:2 | 674.5 | C36H69O8PN | 0.027 | 0.010 | 0.046 | 0.060 |  |
| PE 31:1 | 676.5 | C36H71O8PN | 0.068 | 0.023 | 0.061 | 0.037 |  |
| PE 31:0 | 678.5 | C36H73O8PN | 0.004 | 0.001 | 0.037 | 0.052 |  |
| PE 32:2 | 688.5 | C37H71O8PN | 2.896 | 0.700 | 2.081 | 0.443 |  |
| PE 32:1 | 690.5 | C37H73O8PN | 7.276 | 1.702 | 5.473 | 0.820 |  |
| PE 32:0 | 692.5 | C37H75O8PN | 0.000 | 0.000 | 0.000 | 0.000 |  |
| PE 33:2 | 702.5 | C38H73O8PN | 0.535 | 0.120 | 0.359 | 0.038 |  |
| PE 33:1 | 704.5 | C38H75O8PN | 0.636 | 0.127 | 0.473 | 0.035 |  |
| PE 33:0 | 706.5 | C38H77O8PN | 0.006 | 0.005 | 0.033 | 0.048 |  |
| PE 34:4 | 712.5 | C39H71O8PN | 4.452 | 0.870 | 2.420 | 0.798 |  |
| PE 34:3 | 714.5 | C39H73O8PN | 14.531 | 2.696 | 9.923 | 2.588 |  |
| PE 34:2 | 716.5 | C39H75O8PN | 44.089 | 7.737 | 32.497 | 8.964 |  |
| PE 34:1 | 718.5 | C39H77O8PN | 36.702 | 6.213 | 35.248 | 5.353 |  |
| PE 34:0 | 720.6 | C39H79O8PN | 0.000 | 0.000 | 0.000 | 0.000 |  |
| PE 35:2 | 730.5 | C40H77O8PN | 1.091 | 0.222 | 0.968 | 0.419 |  |
| PE 35:1 | 732.6 | C40H79O8PN | 0.586 | 0.125 | 0.434 | 0.117 |  |
| PE 35:0 | 734.6 | C40H81O8PN | 0.099 | 0.042 | 0.114 | 0.067 |  |
| PE 36:6 | 736.5 | C41H71O8PN | 2.924 | 0.532 | 1.239 | 0.608 |  |
| PE 36:5 | 738.5 | C41H73O8PN | 16.778 | 2.720 | 8.742 | 4.547 |  |
| PE 36:4 | 740.5 | C41H75O8PN | 27.847 | 4.344 | 19.565 | 8.860 |  |
| PE 36:3 | 742.5 | C41H77O8PN | 24.555 | 4.194 | 20.415 | 6.937 |  |
| PE 36:2 | 744.6 | C41H79O8PN | 21.921 | 3.261 | 16.367 | 3.711 |  |
| PE 36:1 | 746.6 | C41H81O8PN | 8.391 | 1.207 | 5.436 | 0.868 |  |
| PE 36:0 | 748.6 | C41H83O8PN | 0.000 | 0.000 | 0.000 | 0.000 | * |
| PE 37:2 | 758.6 | C42H81O8PN | 0.140 | 0.038 | 0.259 | 0.259 |  |
| PE 37:1 | 760.6 | C42H83O8PN | 0.143 | 0.010 | 0.128 | 0.067 |  |
| PE 37:0 | 762.6 | C42H85O8PN | 0.116 | 0.017 | 0.092 | 0.036 | * |
| PE 38:6 | 764.5 | C43H75O8PN | 0.110 | 0.015 | 0.107 | 0.075 | * |
| PE 38:5 | 766.5 | C43H77O8PN | 0.245 | 0.062 | 0.119 | 0.016 |  |
| PE 38:4 | 768.6 | C43H79O8PN | 0.276 | 0.081 | 0.214 | 0.165 |  |
| PE 38:3 | 770.6 | C43H81O8PN | 0.267 | 0.041 | 0.213 | 0.045 |  |
| PE 38:2 | 772.6 | C43H83O8PN | 0.274 | 0.062 | 0.237 | 0.060 |  |
| PE 38:1 | 774.6 | C43H85O8PN | 0.063 | 0.020 | 0.084 | 0.064 |  |
| PE 38:0 | 776.6 | C43H87O8PN | 0.008 | 0.010 | 0.025 | 0.043 |  |
| PE 40:3 | 798.6 | C45H85O8PN | 0.004 | 0.004 | 0.028 | 0.043 | * |
| PE 40:2 | 800.6 | C45H87O8PN | 0.003 | 0.003 | 0.040 | 0.052 | * |
| PE 42:4 | 824.6 | C47H87O8PN | 0.000 | 0.000 | 0.013 | 0.016 |  |
| PE 42:3 | 826.6 | C47H89O8PN | 0.013 | 0.004 | 0.017 | 0.028 |  |
| PE 42:2 | 828.6 | C47H91O8PN | 0.081 | 0.018 | 0.062 | 0.032 |  |
| **Total PE** |  |  | **217.852** | **35.653** | **164.488** | **40.713** | * |
| PI 26:0 | 744.5 | C35H71O13PN | 0.039 | 0.016 | 0.075 | 0.106 |  |
| PI 28:0 | 772.5 | C37H75O13PN | 0.360 | 0.252 | 0.132 | 0.013 |  |
| PI 30:2 | 796.5 | C39H75O13PN | 0.013 | 0.008 | 0.024 | 0.009 |  |
| PI 30:1 | 798.5 | C39H77O13PN | 0.247 | 0.046 | 0.233 | 0.004 |  |
| PI 30:0 | 800.5 | C39H79O13PN | 0.113 | 0.030 | 0.055 | 0.010 |  |
| PI 31:2 | 810.5 | C40H77O13PN | 0.001 | 0.002 | 0.002 | 0.003 | * |
| PI 31:1 | 812.5 | C40H79O13PN | 0.170 | 0.059 | 0.096 | 0.027 |  |
| PI 31:0 | 814.5 | C40H81O13PN | 0.007 | 0.012 | 0.004 | 0.005 |  |
| PI 32:2 | 824.5 | C41H79O13PN | 1.629 | 0.035 | 1.465 | 0.525 |  |
| PI 32:1 | 826.5 | C41H81O13PN | 7.027 | 0.423 | 7.391 | 1.407 |  |
| PI 32:0 | 828.6 | C41H83O13PN | 0.048 | 0.045 | 0.000 | 0.000 |  |
| PI 33:2 | 838.5 | C42H81O13PN | 0.484 | 0.055 | 0.374 | 0.113 |  |
| PI 33:1 | 840.6 | C42H83O13PN | 0.854 | 0.034 | 0.763 | 0.120 |  |
| PI 33:0 | 842.6 | C42H85O13PN | 0.000 | 0.000 | 0.012 | 0.021 |  |
| PI 34:4 | 848.5 | C43H79O13PN | 0.647 | 0.053 | 0.367 | 0.213 |  |
| PI 34:3 | 850.5 | C43H81O13PN | 9.575 | 0.149 | 5.906 | 2.700 |  |
| PI 34:2 | 852.6 | C43H83O13PN | 44.060 | 0.467 | 35.258 | 12.398 |  |
| PI 34:1 | 854.6 | C43H85O13PN | 48.116 | 3.615 | 58.502 | 13.541 |  |
| PI 35:2 | 866.6 | C44H85O13PN | 0.936 | 0.065 | 0.548 | 0.086 |  |
| PI 35:1 | 868.6 | C44H87O13PN | 0.826 | 0.025 | 0.699 | 0.090 |  |
| PI 35:0 | 870.6 | C44H89O13PN | 0.033 | 0.058 | 0.044 | 0.038 |  |
| PI 36:6 | 872.5 | C45H79O13PN | 0.359 | 0.050 | 0.114 | 0.071 |  |
| PI 36:5 | 874.5 | C45H81O13PN | 2.175 | 0.163 | 1.032 | 0.681 |  |
| PI 36:4 | 876.6 | C45H83O13PN | 5.106 | 0.367 | 3.342 | 1.660 |  |
| PI 36:3 | 878.6 | C45H85O13PN | 7.585 | 0.254 | 5.115 | 2.065 |  |
| PI 36:2 | 880.6 | C45H87O13PN | 10.327 | 0.390 | 6.815 | 1.841 |  |
| PI 36:1 | 882.6 | C45H89O13PN | 6.933 | 0.496 | 5.678 | 0.895 |  |
| PI 38:2 | 908.6 | C47H91O13PN | 0.020 | 0.020 | 0.035 | 0.022 |  |
| PI 38:1 | 910.6 | C47H93O13PN | 0.023 | 0.009 | 0.021 | 0.007 |  |
| PI 38:0 | 912.7 | C47H95O13PN | 0.017 | 0.018 | 0.018 | 0.013 |  |
| **Total PI** |  |  | **147.729** | **4.990** | **134.119** | **37.545** | * |
| PS 30:2 | 704.4 | C36H67O10PN | 0.002 | 0.004 | 0.004 | 0.002 |  |
| PS 30:1 | 706.5 | C36H69O10PN | 0.033 | 0.013 | 0.021 | 0.008 |  |
| PS 30:0 | 708.5 | C36H71O10PN | 0.012 | 0.015 | 0.006 | 0.006 | * |
| PS 32:2 | 732.5 | C38H71O10PN | 0.426 | 0.088 | 0.417 | 0.267 |  |
| PS 32:1 | 734.5 | C38H73O10PN | 1.428 | 0.195 | 1.689 | 0.359 |  |
| PS 32:0 | 736.5 | C38H75O10PN | 0.000 | 0.000 | 0.000 | 0.000 |  |
| PS 33:2 | 746.5 | C39H73O10PN | 0.108 | 0.018 | 0.060 | 0.048 | * |
| PS 33:1 | 748.5 | C39H75O10PN | 0.185 | 0.048 | 0.127 | 0.059 |  |
| PS 33:0 | 750.5 | C39H77O10PN | 0.000 | 0.000 | 0.005 | 0.008 |  |
| PS 34:4 | 756.5 | C40H71O10PN | 0.130 | 0.020 | 0.126 | 0.058 |  |
| PS 34:3 | 758.5 | C40H73O10PN | 1.973 | 0.346 | 1.515 | 0.595 |  |
| PS 34:2 | 760.5 | C40H75O10PN | 10.482 | 1.823 | 9.341 | 3.838 |  |
| PS 34:1 | 762.5 | C40H77O10PN | 12.286 | 1.672 | 14.857 | 3.617 |  |
| PS 34:0 | 764.5 | C40H79O10PN | 0.000 | 0.000 | 0.000 | 0.000 | * |
| PS 35:2 | 774.5 | C41H77O10PN | 0.180 | 0.055 | 0.168 | 0.100 | * |
| PS 35:1 | 776.5 | C41H79O10PN | 0.100 | 0.036 | 0.122 | 0.014 |  |
| PS 35:0 | 778.6 | C41H81O10PN | 0.006 | 0.006 | 0.021 | 0.027 |  |
| PS 36:6 | 780.5 | C42H71O10PN | 0.086 | 0.008 | 0.047 | 0.029 |  |
| PS 36:5 | 782.5 | C42H73O10PN | 0.781 | 0.134 | 0.443 | 0.320 |  |
| PS 36:4 | 784.5 | C42H75O10PN | 1.274 | 0.292 | 1.129 | 0.655 |  |
| PS 36:3 | 786.5 | C42H77O10PN | 0.982 | 0.303 | 0.919 | 0.368 |  |
| PS 36:2 | 788.5 | C42H79O10PN | 1.419 | 0.283 | 1.258 | 0.438 |  |
| PS 36:1 | 790.6 | C42H81O10PN | 0.856 | 0.104 | 0.672 | 0.077 |  |
| PS 36:0 | 792.6 | C42H83O10PN | 0.000 | 0.000 | 0.000 | 0.000 |  |
| PS 38:6 | 808.5 | C44H75O10PN | 0.034 | 0.016 | 0.053 | 0.039 |  |
| PS 38:5 | 810.5 | C44H77O10PN | 0.027 | 0.030 | 0.009 | 0.015 |  |
| PS 38:4 | 812.5 | C44H79O10PN | 0.004 | 0.004 | 0.003 | 0.001 | * |
| PS 38:3 | 814.6 | C44H81O10PN | 0.000 | 0.000 | 0.002 | 0.004 |  |
| PS 38:2 | 816.6 | C44H83O10PN | 0.000 | 0.000 | 0.003 | 0.005 |  |
| PS 38:1 | 818.6 | C44H85O10PN | 0.000 | 0.000 | 0.001 | 0.001 |  |
| PS 40:4 | 840.6 | C46H83O10PN | 0.001 | 0.002 | 0.000 | 0.000 |  |
| PS 40:3 | 842.6 | C46H85O10PN | 0.000 | 0.000 | 0.000 | 0.000 |  |
| PS 40:2 | 844.6 | C46H87O10PN | 0.000 | 0.000 | 0.001 | 0.002 |  |
| PS 40:1 | 846.6 | C46H89O10PN | 0.003 | 0.003 | 0.003 | 0.005 |  |
| PS 42:4 | 868.6 | C48H87O10PN | 0.000 | 0.000 | 0.000 | 0.000 |  |
| PS 42:3 | 870.6 | C48H89O10PN | 0.000 | 0.000 | 0.002 | 0.003 |  |
| PS 42:2 | 872.6 | C48H91O10PN | 0.000 | 0.000 | 0.000 | 0.000 | * |
| PS 42:1 | 874.7 | C48H93O10PN | 0.000 | 0.000 | 0.000 | 0.000 | * |
| PS 44:3 | 898.7 | C50H93O10PN | 0.000 | 0.000 | 0.000 | 0.000 |  |
| PS 44:2 | 900.7 | C50H95O10PN | 0.000 | 0.000 | 0.000 | 0.000 |  |
| **TOTAL PS** |  |  | **32.819** | **5.158** | **33.024** | **10.507** | * |
| PG 30:1 | 710.5 | C36H73O10PN | 0.029 | 0.014 | 0.023 | 0.007 | * |
| PG 30:0 | 712.5 | C36H75O10PN | 0.283 | 0.065 | 0.096 | 0.024 |  |
| PG 32:2 | 736.5 | C38H75O10PN | 0.377 | 0.053 | 0.269 | 0.088 | * |
| PG 32:1 | 738.5 | C38H77O10PN | 1.468 | 0.161 | 1.150 | 0.135 | * |
| PG 32:0 | 740.5 | C38H79O10PN | 1.439 | 0.117 | 0.487 | 0.170 |  |
| PG 34:4 | 760.5 | C40H75O10PN | 0.126 | 0.007 | 0.095 | 0.044 |  |
| PG 34:3 | 762.5 | C40H77O10PN | 1.151 | 0.076 | 0.654 | 0.236 | * |
| PG 34:2 | 764.5 | C40H79O10PN | 7.016 | 0.546 | 4.709 | 1.433 |  |
| PG 34:1 | 766.6 | C40H81O10PN | 7.492 | 0.813 | 7.115 | 0.557 |  |
| PG 34:0 | 768.6 | C40H83O10PN | 0.447 | 0.106 | 0.038 | 0.065 |  |
| PG 34:5-O | 774.5 | C40H73O11PN | 0.006 | 0.009 | 0.002 | 0.003 |  |
| PG 34:4-O | 776.5 | C40H75O11PN | 0.046 | 0.008 | 0.008 | 0.009 |  |
| PG 36:6 | 784.5 | C42H75O10PN | 0.085 | 0.019 | 0.029 | 0.020 |  |
| PG 36:5 | 786.5 | C42H77O10PN | 0.487 | 0.031 | 0.286 | 0.183 |  |
| PG 36:4 | 788.5 | C42H79O10PN | 0.824 | 0.039 | 0.751 | 0.332 |  |
| PG 36:3 | 790.6 | C42H81O10PN | 0.530 | 0.061 | 0.616 | 0.165 |  |
| PG 36:2 | 792.6 | C42H83O10PN | 0.694 | 0.063 | 0.651 | 0.083 |  |
| PG 36:1 | 794.6 | C42H85O10PN | 0.294 | 0.030 | 0.222 | 0.020 |  |
| PG 38:6 | 812.5 | C44H79O10PN | 0.001 | 0.002 | 0.001 | 0.001 |  |
| PG 38:5 | 814.6 | C44H81O10PN | 0.001 | 0.001 | 0.001 | 0.001 |  |
| **Total PG** |  |  | **22.795** | **1.873** | **17.205** | **3.431** | * |
| PA 32:2 | 662.5 | C35H69O8PN | 0.681 | 0.061 | 0.810 | 0.209 |  |
| PA 32:1 | 664.5 | C35H71O8PN | 1.154 | 0.162 | 1.404 | 0.197 |  |
| PA 32:0 | 666.5 | C35H73O8PN | 0.032 | 0.024 | 0.004 | 0.007 |  |
| PA 34:6 | 682.4 | C37H65O8PN | 0.000 | 0.000 | 0.003 | 0.003 |  |
| PA 34:5 | 684.5 | C37H67O8PN | 0.011 | 0.009 | 0.017 | 0.016 |  |
| PA 34:4 | 686.5 | C37H69O8PN | 0.496 | 0.046 | 0.467 | 0.236 |  |
| PA 34:3 | 688.5 | C37H71O8PN | 2.874 | 0.263 | 3.042 | 1.102 |  |
| PA 34:2 | 690.5 | C37H73O8PN | 9.376 | 0.686 | 10.878 | 2.585 |  |
| PA 34:1 | 692.5 | C37H75O8PN | 7.725 | 0.918 | 10.939 | 1.629 | * |
| PA 36:6 | 710.5 | C39H69O8PN | 0.225 | 0.043 | 0.115 | 0.075 |  |
| PA 36:5 | 712.5 | C39H71O8PN | 1.622 | 0.055 | 1.263 | 0.725 |  |
| PA 36:4 | 714.5 | C39H73O8PN | 3.769 | 0.312 | 4.084 | 2.055 |  |
| PA 36:3 | 716.5 | C39H75O8PN | 4.968 | 0.359 | 5.942 | 2.192 |  |
| PA 36:2 | 718.5 | C39H77O8PN | 4.978 | 0.288 | 5.298 | 1.111 | * |
| PA 36:1 | 720.6 | C39H79O8PN | 1.360 | 0.103 | 1.157 | 0.079 |  |
| **Total PA** |  |  | **39.271** | **2.773** | **45.424** | **11.456** |  |
| LysoPC 14:1 | 466.3 | C22H45O7PN | 0.006 | 0.001 | 0.020 | 0.023 |  |
| LysoPC 14:0 | 468.3 | C22H47O7PN | 0.136 | 0.010 | 0.186 | 0.033 |  |
| LysoPC 15:1 | 480.3 | C23H47O7PN | 0.001 | 0.000 | 0.008 | 0.009 |  |
| LysoPC 15:0 | 482.3 | C23H49O7PN | 0.017 | 0.003 | 0.025 | 0.017 |  |
| LysoPC 16:1 | 494.3 | C24H49O7PN | 1.438 | 0.344 | 2.121 | 0.416 |  |
| LysoPC 16:0 | 496.3 | C24H51O7PN | 1.235 | 0.146 | 1.221 | 0.264 |  |
| LysoPC 17:1 | 508.3 | C25H51O7PN | 0.073 | 0.010 | 0.093 | 0.013 |  |
| LysoPC 17:0 | 510.4 | C25H53O7PN | 0.038 | 0.012 | 0.038 | 0.023 |  |
| LysoPC 18:3 | 518.3 | C26H49O7PN | 1.634 | 0.347 | 1.471 | 0.649 |  |
| LysoPC 18:2 | 520.3 | C26H51O7PN | 6.235 | 1.775 | 9.082 | 3.979 |  |
| LysoPC 18:1 | 522.4 | C26H53O7PN | 4.634 | 1.166 | 8.931 | 2.517 |  |
| LysoPC 18:0 | 524.4 | C26H55O7PN | 0.519 | 0.121 | 0.369 | 0.178 |  |
| **Total LysoPC** | |  | **15.966** | **3.840** | **23.563** | **7.224** |  |
| LysoPE 14:1 | 424.2 | C19H39O7PN | 0.000 | 0.000 | 0.010 | 0.017 |  |
| LysoPE 15:1 | 438.3 | C20H41O7PN | 0.000 | 0.000 | 0.000 | 0.000 |  |
| LysoPE 15:0 | 440.3 | C20H43O7PN | 0.007 | 0.012 | 0.009 | 0.016 |  |
| LysoPE 16:1 | 452.3 | C21H43O7PN | 0.500 | 0.096 | 0.544 | 0.156 |  |
| LysoPE 16:0 | 454.3 | C21H45O7PN | 1.341 | 0.276 | 1.063 | 0.204 |  |
| LysoPE 17:1 | 466.3 | C22H45O7PN | 0.003 | 0.004 | 0.011 | 0.007 |  |
| LysoPE 17:0 | 468.3 | C22H47O7PN | 0.008 | 0.015 | 0.006 | 0.005 |  |
| LysoPE 18:3 | 476.3 | C23H43O7PN | 0.529 | 0.137 | 0.372 | 0.111 |  |
| LysoPE 18:2 | 478.3 | C23H45O7PN | 1.853 | 0.542 | 2.089 | 0.903 |  |
| LysoPE 18:1 | 480.3 | C23H47O7PN | 1.798 | 0.608 | 2.640 | 0.731 |  |
| LysoPE 20:1 | 508.3 | C25H51O7PN | 0.004 | 0.006 | 0.004 | 0.003 |  |
| LysoPE 20:0 | 510.4 | C25H53O7PN | 0.000 | 0.000 | 0.000 | 0.000 | * |
| **Total LysoPE** | |  | **6.042** | **1.607** | **6.749** | **1.935** | * |
| LysoPG 16:1 | 481.3 | C22H42O9P | 0.014 | 0.023 | 0.028 | 0.007 |  |
| LysoPG 16:0 | 483.3 | C22H44O9P | 0.173 | 0.029 | 0.145 | 0.065 |  |
| LysoPG 18:3 | 505.3 | C24H42O9P | 0.063 | 0.033 | 0.107 | 0.019 |  |
| LysoPG 18:2 | 507.3 | C24H44O9P | 0.114 | 0.017 | 0.116 | 0.030 |  |
| LysoPG 18:1 | 509.3 | C24H46O9P | 0.124 | 0.016 | 0.133 | 0.014 | * |
| **Total lysoPG** | |  | **0.488** | **0.076** | **0.530** | **0.107** |  |
| **Total** |  |  | **867.448** | **64.258** | **775.261** | **177.252** |  |
