# Supplementary material for: Calcineurin Signaling and Membrane Lipid Homeostasis Regulates Iron Mediated MultiDrug Resistance Mechanisms in Candida albicans
Source: PLoS One. 2011 Apr 12;6(4):e18684. doi: 10.1371/journal.pone.0018684 (PMC3075269; doi:10.1371/journal.pone.0018684)
Supplement: Table S4 — The relative abundance of SL compositions in response to iron deprivation. Values are mean of ± SD (n = 3 for conditions, * depicts P value<0.05). Data is represented as nmoles/mg dry lipid weight (normalized SL mass spectral signal). (DOC) [file pone.0018684.s006.doc]

**Table: S4**

| **Sphingolipid amounts** | |  |  |  |  |  |  |
| --- | --- | --- | --- | --- | --- | --- | --- |
|  |  |  | nmol per mg dry wt | | nmol per mg dry wt | |  |
|  |  |  | **WT** |  | **WT+BPS** |  | p-value <0.05 |
| **Sample description** | **Masses** | **Formula** | **Average** | **Stdev** | **Average** | **Stdev** |  |
| Cer C44:0;3 | 667.0 | C44O3H76N | 35.940 | 10.715 | 32.146 | 10.017 |  |
| Cer C46:0;3 | 695.0 | C46O3H80N | 0.175 | 0.057 | 0.191 | 0.123 |  |
| **Total CER** |  |  | **36.115** | **10.770** | **32.337** | **10.127** |  |
| IPC 48:0;3 | 896.6 | C48O12H83PN | 0.155 | 0.011 | 0.057 | 0.018 | * |
| IPC 48:0;4 | 912.6 | C48O13H83PN | 0.005 | 0.004 | 0.005 | 0.005 |  |
| IPC 50:0;3 | 924.6 | C50O12H87PN | 8.434 | 1.094 | 4.170 | 0.234 | * |
| IPC 48:0;5 | 928.6 | C48O14H83PN | 0.008 | 0.003 | 0.005 | 0.005 |  |
| IPC 50:0;4 | 940.6 | C50O13H87PN | 3.034 | 0.247 | 2.170 | 0.269 | * |
| IPC 52:0;3 | 952.6 | C52O12H91PN | 7.353 | 0.250 | 3.519 | 0.711 | * |
| IPC 50:0;5 | 956.6 | C50O14H87PN | 0.030 | 0.006 | 0.019 | 0.003 | * |
| IPC 52:0;4 | 968.6 | C52O13H91PN | 0.565 | 0.084 | 0.453 | 0.078 |  |
| IPC 52:0;5 | 984.6 | C52O14H91PN | 0.023 | 0.012 | 0.013 | 0.005 |  |
| **Total IPC** |  |  | **19.606** | **1.014** | **10.410** | **1.252** | * |
| MIPC 54:0;3 | 1058.6 | C54O17H93PN | 0.036 | 0.001 | 0.011 | 0.010 | * |
| MIPC 56:0;3 | 1086.6 | C56O17H97PN | 4.013 | 0.645 | 3.127 | 0.437 |  |
| MIPC 54:0;5 | 1090.6 | C54O19H93PN | 0.002 | 0.002 | 0.006 | 0.003 |  |
| MIPC 56:0;4 | 1102.6 | C56O18H97PN | 0.902 | 0.440 | 1.075 | 0.294 |  |
| MIPC 58:0;3 | 1114.7 | C58O17H101PN | 3.519 | 0.776 | 2.657 | 0.640 |  |
| MIPC 56:0;5 | 1118.6 | C56O19H97PN | 0.004 | 0.004 | 0.008 | 0.005 |  |
| MIPC 58:0;4 | 1130.7 | C58O18H101PN | 0.167 | 0.072 | 0.219 | 0.060 |  |
| MIPC 58:0;5 | 1146.7 | C58O19H1011PN | 0.014 | 0.018 | 0.011 | 0.013 |  |
| **Total MIPC** | |  | **8.657** | **1.887** | **7.114** | **1.438** |  |
| M(IP)_2_C 62:0;3 | 1329.0 | C62O25H108P2N | 0.037 | 0.063 | 0.199 | 0.218 |  |
| M(IP)_2_C 62:0;4 | 1345.0 | C62O26H108P2N | 0.000 | 0.000 | 0.014 | 0.021 |  |
| M(IP)_2_C 64:0;3 | 1357.0 | C64O25H112P2N | 0.016 | 0.028 | 0.189 | 0.220 |  |
| M(IP)_2_C 64:0;4 | 1373.0 | C64O26H112P2N | 0.000 | 0.000 | 0.001 | 0.002 |  |
| **Total M(IP)** _2_**C** | |  | **0.053** | **0.092** | **0.403** | **0.460** |  |
| **Total SL** |  |  | **64.409** | **11.783** | **50.251** | **12.390** |  |
