# Supplementary material for: Calcineurin Signaling and Membrane Lipid Homeostasis Regulates Iron Mediated MultiDrug Resistance Mechanisms in Candida albicans
Source: PLoS One. 2011 Apr 12;6(4):e18684. doi: 10.1371/journal.pone.0018684 (PMC3075269; doi:10.1371/journal.pone.0018684)
Supplement: Table S5 — The relative abundance of SE compositions in response to iron deprivation. Values are means of ± SD (n = 3 for conditions, * depicts P value<0.05). Data is represented as nmoles/mg dry lipid weight (normalized SE mass spectral signal). (DOC) [file pone.0018684.s007.doc]

**Table: S5**

| **Sterol ester amounts** | |  |  |  |  |  |  |
| --- | --- | --- | --- | --- | --- | --- | --- |
|  |  |  | nmol per mg dry wt | | nmol per mg dry wt | |  |
|  |  |  | **WT** |  | **WT+BPS** |  | p-value <0.05 |
| **Sample description** | **Masses** | **Formula** | **Average** | **Stdev** | **Average** | **Stdev** |  |
| Zymosterol | 638.7 | 16:1 containing | 0.053 | 0.032 | 0.001 | 0.003 | * |
| Ergostatetraenol | 648.9 | 16:1 containing | 0.000 | 0.000 | 0.000 | 0.000 |  |
| Ergosterol | 650.7 | 16:1 containing | 0.029 | 0.026 | 0.000 | 0.000 |  |
| Episterol + Fecosterol | 652.7 | 16:1 containing | 0.060 | 0.017 | 0.067 | 0.048 |  |
| Lanosterol | 680.7 | 16:1 containing | 0.278 | 0.075 | 0.100 | 0.066 | * |
| **Total 16:1** |  |  | **0.420** | **0.112** | **0.169** | **0.112** | * |
| Zymosterol | 640.7 | 16:0 containing | 0.151 | 0.028 | 0.058 | 0.033 | * |
| Ergostatetraenol | 650.9 | 16:0 containing | 0.000 | 0.000 | 0.003 | 0.005 |  |
| Ergosterol | 652.7 | 16:0 containing | 0.123 | 0.051 | 0.069 | 0.052 |  |
| Episterol + Fecosterol | 654.7 | 16:0 containing | 0.562 | 0.130 | 0.578 | 0.264 |  |
| Lanosterol | 682.7 | 16:0 containing | 1.653 | 0.549 | 1.729 | 0.087 |  |
| **Total 16:0** |  |  | **2.489** | **0.648** | **2.436** | **0.336** |  |
| Zymosterol | 662.7 | 18:3 containing | 0.150 | 0.014 | 0.010 | 0.017 | * |
| Ergostatetraenol | 673.0 | 18:3 containing | 0.056 | 0.033 | 0.005 | 0.006 | * |
| Ergosterol | 674.7 | 18:3 containing | 0.749 | 0.187 | 0.086 | 0.084 | * |
| Episterol + Fecosterol | 676.7 | 18:3 containing | 0.680 | 0.045 | 0.337 | 0.294 |  |
| Lanosterol | 704.7 | 18:3 containing | 2.611 | 0.011 | 1.596 | 1.023 |  |
| **Total 18:3** |  |  | **4.257** | **0.246** | **2.041** | **1.385** | * |
| Zymosterol | 664.7 | 18:2 containing | 0.441 | 0.030 | 0.129 | 0.112 | * |
| Ergostatetraenol | 675.0 | 18:2 containing | 0.088 | 0.045 | 0.002 | 0.003 | * |
| Ergosterol | 676.7 | 18:2 containing | 1.622 | 0.263 | 0.292 | 0.249 | * |
| Episterol + Fecosterol | 678.7 | 18:2 containing | 2.053 | 0.104 | 1.396 | 1.045 |  |
| Lanosterol | 706.7 | 18:2 containing | 2.592 | 0.216 | 2.207 | 1.120 |  |
| **Total 18:2** |  |  | **6.797** | **0.330** | **4.026** | **2.447** |  |
| Zymosterol | 666.6 | 18:1 containing | 0.590 | 0.049 | 0.160 | 0.091 | * |
| Ergostatetraenol | 677.0 | 18:1 containing | 0.056 | 0.043 | 0.005 | 0.006 |  |
| Ergosterol | 678.6 | 18:1 containing | 1.000 | 0.123 | 0.249 | 0.203 | * |
| Episterol + Fecosterol | 680.7 | 18:1 containing | 1.527 | 0.251 | 1.111 | 0.655 |  |
| Lanosterol | 708.7 | 18:1 containing | 2.442 | 0.144 | 2.061 | 0.586 |  |
| **Total 18:1** |  |  | **5.615** | **0.308** | **3.586** | **1.523** | * |
| Zymosterol | 668.6 | 18:0 containing | 0.048 | 0.007 | 0.008 | 0.009 | * |
| Ergostatetraenol | 678.6 | 18:0 containing | 0.002 | 0.004 | 0.000 | 0.000 |  |
| Ergosterol | 680.6 | 18:0 containing | 0.104 | 0.048 | 0.027 | 0.017 | * |
| Episterol + Fecosterol | 682.6 | 18:0 containing | 0.289 | 0.091 | 0.251 | 0.107 |  |
| Lanosterol | 710.6 | 18:0 containing | 0.494 | 0.036 | 0.593 | 0.129 |  |
| **Total 18:0** |  |  | **0.937** | **0.141** | **0.878** | **0.204** |  |
| **Total SE** |  |  | **20.515** | **1.532** | **13.136** | **5.940** |  |
|  |  |  |  |  |  |  |  |
|  |  |  |  |  |  |  |  |
| **Zymosterol SE** | |  | 1.433 | 0.050 | 0.366 | 0.180 | * |
| **Ergostatetraenol SE** | |  | 0.202 | 0.051 | 0.015 | 0.014 | * |
| **Ergosterol SE** | |  | 3.628 | 0.545 | 0.722 | 0.565 | * |
| **Episterol + Fecosterol SE** | | | 5.171 | 0.502 | 3.739 | 2.392 |  |
| **Lanosterol SE** | |  | 10.070 | 0.834 | 8.286 | 2.904 |  |
| **Total SE** |  |  | **20.503** | **1.539** | **13.129** | **5.953** |  |
