# Supplementary material for: Calcineurin Signaling and Membrane Lipid Homeostasis Regulates Iron Mediated MultiDrug Resistance Mechanisms in Candida albicans
Source: PLoS One. 2011 Apr 12;6(4):e18684. doi: 10.1371/journal.pone.0018684 (PMC3075269; doi:10.1371/journal.pone.0018684)
Supplement: Table S6 — The relative abundance of DAG based on fatty acid chain compositions in response to iron deprivation. Values are mean of ± SD (n = 3 for conditions, * depicts P value<0.05). Data is represented as nmoles/mg dry lipid weight (normalized DAG mass spectral signal). (DOC) [file pone.0018684.s008.doc]

**Table: S6**

| **DAG amounts** | |  |  |  | |  | |  | | |  |  |
| --- | --- | --- | --- | --- | --- | --- | --- | --- | --- | --- | --- | --- |
|  |  |  |  | nmol per mg dry wt | | | nmol per mg dry wt | | |  | | |
|  |  |  |  | **WT** |  | | **WT+BPS** | |  | p-value <0.05 | | |
| **Sample description** | **Masses** | **Formula** |  | **Average** | **Stdev** | | **Average** | | **Stdev** |  | | |
| 30:1 | 556.5 | C33H66O5N | 16:1 containing | 0.005 | 0.005 | | 0.008 | | 0.008 |  | | |
| 32:1 | 584.5 | C35H70O5N | 16:1 containing | 0.138 | 0.057 | | 0.525 | | 0.587 |  | | |
| 34:4 | 606.5 | C37H68O5N | 16:1 containing | 0.128 | 0.073 | | 0.053 | | 0.047 |  | | |
| 34:3 | 608.5 | C37H70O5N | 16:1 containing | 0.626 | 0.144 | | 0.272 | | 0.111 | * | | |
| 34:2 | 610.5 | C37H72O5N | 16:1 containing | 0.524 | 0.086 | | 0.379 | | 0.048 | * | | |
| 34:1 | 612.6 | C37H74O5N | 16:1 containing | 0.030 | 0.044 | | 0.036 | | 0.032 |  | | |
| 36:2 | 638.6 | C39H76O5N | 16:1 containing | 0.053 | 0.032 | | 0.001 | | 0.003 | * | | |
| 36:1 | 640.6 | C39H78O5N | 16:1 containing | 0.009 | 0.008 | | 0.013 | | 0.007 |  | | |
| 36:0 | 642.6 | C39H80O5N | 16:1 containing | 0.356 | 0.617 | | 0.546 | | 0.649 |  | | |
| 38:7 | 656.5 | C41H70O5N | 16:1 containing | 0.000 | 0.000 | | 0.663 | | 1.148 |  | | |
| 38:6 | 658.5 | C41H72O5N | 16:1 containing | 0.000 | 0.000 | | 0.132 | | 0.229 |  | | |
| 38:4 | 662.6 | C41H76O5N | 16:1 containing | 0.000 | 0.000 | | 0.001 | | 0.003 |  | | |
| 38:3 | 664.6 | C41H78O5N | 16:1 containing | 0.002 | 0.003 | | 0.009 | | 0.010 |  | | |
| 40:7 | 684.6 | C43H74O5N | 16:1 containing | 0.006 | 0.007 | | 0.002 | | 0.003 |  | | |
| 40:5 | 688.6 | C43H78O5N | 16:1 containing | 0.002 | 0.003 | | 0.000 | | 0.000 |  | | |
| **Total 16:1** |  |  |  | **1.878** | **0.766** | | **2.640** | | **1.699** |  | | |
| 26:0 | 502.4 | C29H60O5N | 16:0 containing | 0.031 | 0.027 | | 0.032 | | 0.038 |  | | |
| 28:0 | 530.5 | C31H64O5N | 16:0 containing | 0.050 | 0.054 | | 0.019 | | 0.022 |  | | |
| 30:1 | 556.5 | C33H66O5N | 16:0 containing | 0.006 | 0.010 | | 0.033 | | 0.050 |  | | |
| 32:1 | 584.5 | C35H70O5N | 16:0 containing | 1.048 | 0.112 | | 1.423 | | 0.904 |  | | |
| 32:0 | 586.5 | C35H72O5N | 16:0 containing | 0.278 | 0.067 | | 0.469 | | 0.334 |  | | |
| 34:3 | 608.5 | C37H70O5N | 16:0 containing | 1.076 | 0.097 | | 0.448 | | 0.147 | * | | |
| 34:2 | 610.5 | C37H72O5N | 16:0 containing | 7.366 | 0.469 | | 5.023 | | 0.461 | * | | |
| 34:1 | 612.6 | C37H74O5N | 16:0 containing | 5.943 | 0.375 | | 6.417 | | 1.844 |  | | |
| 34:0 | 614.6 | C37H76O5N | 16:0 containing | 0.103 | 0.104 | | 0.162 | | 0.134 |  | | |
| 36:6 | 630.5 | C39H68O5N | 16:0 containing | 0.004 | 0.006 | | 0.206 | | 0.303 |  | | |
| 36:3 | 636.6 | C39H74O5N | 16:0 containing | 0.006 | 0.011 | | 0.002 | | 0.003 |  | | |
| 36:2 | 638.6 | C39H76O5N | 16:0 containing | 0.010 | 0.014 | | 0.056 | | 0.014 | * | | |
| 36:1 | 640.6 | C39H78O5N | 16:0 containing | 0.151 | 0.028 | | 0.057 | | 0.033 | * | | |
| 36:0 | 642.6 | C39H80O5N | 16:0 containing | 0.090 | 0.087 | | 0.157 | | 0.125 |  | | |
| 38:7 | 656.5 | C41H70O5N | 16:0 containing | 0.638 | 0.259 | | 1.417 | | 0.748 |  | | |
| 38:6 | 658.5 | C41H72O5N | 16:0 containing | 0.002 | 0.004 | | 0.197 | | 0.281 |  | | |
| 38:5 | 660.6 | C41H74O5N | 16:0 containing | 0.008 | 0.008 | | 0.025 | | 0.032 |  | | |
| 38:3 | 664.6 | C41H78O5N | 16:0 containing | 0.011 | 0.018 | | 0.024 | | 0.029 |  | | |
| 40:7 | 684.6 | C43H74O5N | 16:0 containing | 0.494 | 0.329 | | 0.771 | | 0.376 |  | | |
| 40:6 | 686.6 | C43H76O5N | 16:0 containing | 0.027 | 0.028 | | 0.037 | | 0.033 |  | | |
| 40:5 | 688.6 | C43H78O5N | 16:0 containing | 0.019 | 0.009 | | 0.060 | | 0.054 |  | | |
| **Total 16:0** |  |  |  | **17.362** | **0.891** | | **17.035** | | **4.669** |  | | |
| 34:5 | 604.5 | C37H66O5N | 18:3 containing | 0.000 | 0.000 | | 0.000 | | 0.000 |  | | |
| 34:4 | 606.5 | C37H68O5N | 18:3 containing | 0.364 | 0.133 | | 0.210 | | 0.052 |  | | |
| 34:3 | 608.5 | C37H70O5N | 18:3 containing | 0.488 | 0.067 | | 0.311 | | 0.009 | * | | |
| 36:6 | 630.5 | C39H68O5N | 18:3 containing | 0.391 | 0.166 | | 0.032 | | 0.035 | * | | |
| 36:5 | 632.5 | C39H70O5N | 18:3 containing | 1.555 | 0.046 | | 0.726 | | 0.297 | * | | |
| 36:4 | 634.5 | C39H72O5N | 18:3 containing | 1.938 | 0.173 | | 1.115 | | 0.366 | * | | |
| 36:3 | 636.6 | C39H74O5N | 18:3 containing | 0.151 | 0.021 | | 0.069 | | 0.072 |  | | |
| 38:4 | 662.6 | C41H76O5N | 18:3 containing | 0.150 | 0.014 | | 0.010 | | 0.017 | * | | |
| 40:7 | 684.6 | C43H74O5N | 18:3 containing | 0.000 | 0.000 | | 0.000 | | 0.000 |  | | |
| 40:6 | 686.6 | C43H76O5N | 18:3 containing | 0.065 | 0.102 | | 0.013 | | 0.002 |  | | |
| 40:5 | 688.6 | C43H78O5N | 18:3 containing | 0.020 | 0.015 | | 0.013 | | 0.003 |  | | |
| **Total 18:3** |  |  |  | **5.122** | **0.318** | | **2.499** | | **0.693** | * | | |
| 26:0 | 502.4 | C29H60O5N | 18:2 containing | 0.023 | 0.007 | | 0.018 | | 0.014 |  | | |
| 34:4 | 606.5 | C37H68O5N | 18:2 containing | 0.038 | 0.027 | | 0.006 | | 0.011 |  | | |
| 34:3 | 608.5 | C37H70O5N | 18:2 containing | 1.641 | 0.048 | | 1.743 | | 0.334 |  | | |
| 34:2 | 610.5 | C37H72O5N | 18:2 containing | 5.258 | 0.311 | | 3.914 | | 0.402 | * | | |
| 36:6 | 630.5 | C39H68O5N | 18:2 containing | 0.017 | 0.006 | | 0.002 | | 0.003 | * | | |
| 36:5 | 632.5 | C39H70O5N | 18:2 containing | 1.978 | 0.243 | | 0.738 | | 0.396 | * | | |
| 36:4 | 634.5 | C39H72O5N | 18:2 containing | 5.379 | 0.412 | | 3.777 | | 1.303 |  | | |
| 36:3 | 636.6 | C39H74O5N | 18:2 containing | 4.493 | 0.380 | | 3.911 | | 0.833 |  | | |
| 36:2 | 638.6 | C39H76O5N | 18:2 containing | 1.365 | 0.144 | | 0.822 | | 0.099 | * | | |
| 38:5 | 660.6 | C41H74O5N | 18:2 containing | 0.002 | 0.004 | | 0.004 | | 0.007 |  | | |
| 38:4 | 662.6 | C41H76O5N | 18:2 containing | 0.024 | 0.006 | | 0.005 | | 0.005 | * | | |
| 38:3 | 664.6 | C41H78O5N | 18:2 containing | 0.441 | 0.030 | | 0.129 | | 0.112 | * | | |
| 40:5 | 688.6 | C43H78O5N | 18:2 containing | 0.029 | 0.035 | | 0.059 | | 0.023 |  | | |
| **Total 18:2** |  |  |  | **20.687** | **0.993** | | **15.128** | | **2.329** | * | | |
| 28:1 | 528.5 | C31H62O5N | 18:1 containing | 0.002 | 0.002 | | 0.006 | | 0.005 |  | | |
| 30:1 | 556.5 | C33H66O5N | 18:1 containing | 0.035 | 0.010 | | 0.017 | | 0.011 | * | | |
| 32:1 | 584.5 | C35H70O5N | 18:1 containing | 0.233 | 0.070 | | 0.401 | | 0.102 | * | | |
| 32:0 | 586.5 | C35H72O5N | 18:1 containing | 0.000 | 0.000 | | 0.000 | | 0.000 |  | | |
| 34:6 | 602.5 | C37H64O5N | 18:1 containing | 0.009 | 0.009 | | 0.005 | | 0.006 |  | | |
| 34:3 | 608.5 | C37H70O5N | 18:1 containing | 0.044 | 0.011 | | 0.058 | | 0.061 |  | | |
| 34:2 | 610.5 | C37H72O5N | 18:1 containing | 1.592 | 0.082 | | 1.549 | | 0.251 |  | | |
| 34:1 | 612.6 | C37H74O5N | 18:1 containing | 4.675 | 0.294 | | 5.453 | | 1.058 |  | | |
| 34:0 | 614.6 | C37H76O5N | 18:1 containing | 0.000 | 0.000 | | 0.000 | | 0.000 |  | | |
| 36:5 | 632.5 | C39H70O5N | 18:1 containing | 0.017 | 0.015 | | 0.020 | | 0.018 |  | | |
| 36:4 | 634.5 | C39H72O5N | 18:1 containing | 2.313 | 0.232 | | 1.260 | | 0.305 | * | | |
| 36:3 | 636.6 | C39H74O5N | 18:1 containing | 4.552 | 0.093 | | 3.520 | | 0.326 | * | | |
| 36:2 | 638.6 | C39H76O5N | 18:1 containing | 5.199 | 0.443 | | 4.404 | | 0.508 |  | | |
| 36:1 | 640.6 | C39H78O5N | 18:1 containing | 1.175 | 0.014 | | 0.934 | | 0.178 | * | | |
| 36:0 | 642.6 | C39H80O5N | 18:1 containing | 0.000 | 0.000 | | 0.000 | | 0.000 |  | | |
| 38:7 | 656.5 | C41H70O5N | 18:1 containing | 0.004 | 0.006 | | 0.000 | | 0.000 |  | | |
| 38:4 | 662.6 | C41H76O5N | 18:1 containing | 0.002 | 0.003 | | 0.000 | | 0.000 |  | | |
| 38:3 | 664.6 | C41H78O5N | 18:1 containing | 0.005 | 0.008 | | 0.002 | | 0.004 |  | | |
| 40:7 | 684.6 | C43H74O5N | 18:1 containing | 0.000 | 0.000 | | 0.011 | | 0.010 |  | | |
| 40:5 | 688.6 | C43H78O5N | 18:1 containing | 0.000 | 0.000 | | 0.000 | | 0.000 |  | | |
| **Total 18:1** |  |  |  | **19.855** | **1.050** | | **17.641** | | **1.707** |  | | |
| 28:0 | 530.5 | C31H64O5N | 18:0 containing | 0.005 | 0.008 | | 0.007 | | 0.013 |  | | |
| 32:1 | 584.5 | C35H70O5N | 18:0 containing | 0.003 | 0.005 | | 0.002 | | 0.003 |  | | |
| 32:0 | 586.5 | C35H72O5N | 18:0 containing | 0.033 | 0.019 | | 0.011 | | 0.009 |  | | |
| 34:6 | 602.5 | C37H64O5N | 18:0 containing | 0.002 | 0.003 | | 0.000 | | 0.000 |  | | |
| 34:1 | 612.6 | C37H74O5N | 18:0 containing | 0.246 | 0.106 | | 0.283 | | 0.093 |  | | |
| 34:0 | 614.6 | C37H76O5N | 18:0 containing | 0.227 | 0.178 | | 0.292 | | 0.135 |  | | |
| 36:6 | 630.5 | C39H68O5N | 18:0 containing | 0.000 | 0.000 | | 0.000 | | 0.000 |  | | |
| 36:3 | 636.6 | C39H74O5N | 18:0 containing | 0.337 | 0.050 | | 0.106 | | 0.041 | * | | |
| 36:2 | 638.6 | C39H76O5N | 18:0 containing | 1.459 | 0.193 | | 0.909 | | 0.081 | * | | |
| 36:1 | 640.6 | C39H78O5N | 18:0 containing | 1.361 | 0.227 | | 1.107 | | 0.315 |  | | |
| 36:0 | 642.6 | C39H80O5N | 18:0 containing | 0.043 | 0.050 | | 0.147 | | 0.115 |  | | |
| 38:7 | 656.5 | C41H70O5N | 18:0 containing | 0.039 | 0.035 | | 0.255 | | 0.410 |  | | |
| 38:6 | 658.5 | C41H72O5N | 18:0 containing | 0.000 | 0.000 | | 0.000 | | 0.000 |  | | |
| 38:3 | 664.6 | C41H78O5N | 18:0 containing | 0.000 | 0.000 | | 0.004 | | 0.008 |  | | |
| 40:7 | 684.6 | C43H74O5N | 18:0 containing | 0.138 | 0.015 | | 0.347 | | 0.204 |  | | |
| **Total 18:0** |  |  |  | **3.892** | **0.494** | | **3.471** | | **1.065** |  | | |
