# Supplementary material for: Calcineurin Signaling and Membrane Lipid Homeostasis Regulates Iron Mediated MultiDrug Resistance Mechanisms in Candida albicans
Source: PLoS One. 2011 Apr 12;6(4):e18684. doi: 10.1371/journal.pone.0018684 (PMC3075269; doi:10.1371/journal.pone.0018684)
Supplement: Table S7 — The relative abundance of TAG based on fatty acid chain compositions in response to iron deprivation. Values are mean of ± SD (n = 3 for conditions, * depicts P value<0.05). Data is represented as nmoles/mg dry lipid weight (normalized TAG mass spectral signal). (DOC) [file pone.0018684.s009.doc]

**Table: S7**

| **TAG amounts** | |  |  |  | | |  |  | | |  |  |
| --- | --- | --- | --- | --- | --- | --- | --- | --- | --- | --- | --- | --- |
|  |  |  |  | nmol per mg dry wt | | nmol per mg dry wt | | | |  |  | |
|  |  |  |  | **WT** |  | **WT+BPS** | | |  | p-value <0.05 |  | |
| **Sample description** | | **Masses** | **Formula** | **Average** | **Stdev** | **Average** | | | **Stdev** |  |  | |
| 44:2 | 16:1 containing | 764.7 | C47H90O6N | 0.000 | 0.000 | 0.001 | | | 0.002 |  |  | |
| 46:3 | 16:1 containing | 790.7 | C49H92O6N | 0.000 | 0.000 | 0.045 | | | 0.077 |  |  | |
| 46:2 | 16:1 containing | 792.7 | C49H94O6N | 0.002 | 0.003 | 0.020 | | | 0.029 |  |  | |
| 46:1 | 16:1 containing | 794.7 | C49H96O6N | 0.002 | 0.003 | 0.005 | | | 0.002 |  |  | |
| 47:2 | 16:1 containing | 804.7 | C50H94O6N | 0.000 | 0.000 | 0.013 | | | 0.023 |  |  | |
| 47:1 | 16:1 containing | 806.7 | C50H96O6N | 0.001 | 0.002 | 0.002 | | | 0.004 |  |  | |
| M? | 16:1 containing | 816.7 | C51H94O6N | 0.002 | 0.002 | 0.000 | | | 0.000 |  |  | |
| 48:3 | 16:1 containing | 818.7 | C51H96O6N | 0.012 | 0.002 | 0.011 | | | 0.003 |  |  | |
| 48:2 | 16:1 containing | 820.7 | C51H98O6N | 0.017 | 0.001 | 0.025 | | | 0.004 | * |  | |
| 48:1 | 16:1 containing | 822.8 | C51H100O6N | 0.005 | 0.007 | 0.005 | | | 0.003 |  |  | |
| O? | 16:1 containing | 832.7 | C52H98O6N | 0.000 | 0.000 | 0.000 | | | 0.000 | * |  | |
| 49:2 | 16:1 containing | 834.8 | C52H100O6N | 0.001 | 0.002 | 0.003 | | | 0.001 |  |  | |
| 50:4 | 16:1 containing | 844.7 | C53H98O6N | 0.042 | 0.006 | 0.018 | | | 0.010 | * |  | |
| 50:3 | 16:1 containing | 846.8 | C53H100O6N | 0.079 | 0.015 | 0.048 | | | 0.014 | * |  | |
| 50:2 | 16:1 containing | 848.8 | C53H102O6N | 0.051 | 0.024 | 0.041 | | | 0.002 |  |  | |
| 50:1 | 16:1 containing | 850.8 | C53H104O6N | 0.002 | 0.003 | 0.004 | | | 0.004 |  |  | |
| 864? | 16:1 containing | 864.7 |  | 0.000 | 0.000 | 0.007 | | | 0.013 |  |  | |
| 866? | 16:1 containing | 866.7 |  | 0.000 | 0.000 | 0.003 | | | 0.006 |  |  | |
| 52:6 | 16:1 containing | 868.7 | C55H98O6N | 0.032 | 0.010 | 0.013 | | | 0.012 |  |  | |
| 52:5 | 16:1 containing | 870.8 | C55H100O6N | 0.081 | 0.014 | 0.031 | | | 0.020 | * |  | |
| 52:4 | 16:1 containing | 872.8 | C55H102O6N | 0.088 | 0.028 | 0.057 | | | 0.024 |  |  | |
| 52:3 | 16:1 containing | 874.8 | C55H104O6N | 0.057 | 0.017 | 0.030 | | | 0.010 | * |  | |
| 52:2 | 16:1 containing | 876.8 | C55H106O6N | 0.012 | 0.006 | 0.011 | | | 0.001 |  |  | |
| 52:1 | 16:1 containing | 878.8 | C55H108O6N | 0.000 | 0.000 | 0.009 | | | 0.014 |  |  | |
| 886? | 16:1 containing | 886.8 |  | 0.001 | 0.000 | 0.000 | | | 0.001 |  |  | |
| 53:3 | 16:1 containing | 888.8 | C56H106O6N | 0.000 | 0.000 | 0.001 | | | 0.001 |  |  | |
| 58:2 | 16:1 containing | 960.9 | C61H118O6N | 0.000 | 0.001 | 0.000 | | | 0.000 |  |  | |
| **Total 16:1 TAG** | |  |  | **0.485** | **0.122** | **0.403** | | | **0.094** |  |  | |
| 40:4 | 16:0 containing | 704.6 | C43H78O6N | 0.000 | 0.000 | 0.024 | | | 0.038 |  |  | |
| 40:0 | 16:0 containing | 712.6 | C43H86O6N | 0.003 | 0.004 | 0.005 | | | 0.003 |  |  | |
| 42:0 | 16:0 containing | 740.7 | C45H90O6N | 0.006 | 0.008 | 0.014 | | | 0.008 |  |  | |
| 43:0 | 16:0 containing | 754.7 | C46H92O6N | 0.004 | 0.007 | 0.005 | | | 0.005 |  |  | |
| E? | 16:0 containing | 756.7 | C46H94O6N | 0.004 | 0.004 | 0.011 | | | 0.009 |  |  | |
| 44:2 | 16:0 containing | 764.7 | C47H90O6N | 0.001 | 0.001 | 0.009 | | | 0.005 | * |  | |
| 44:1 | 16:0 containing | 766.7 | C47H92O6N | 0.007 | 0.007 | 0.015 | | | 0.011 |  |  | |
| 44:0 | 16:0 containing | 768.7 | C47H94O6N | 0.018 | 0.012 | 0.031 | | | 0.018 |  |  | |
| 45:3 | 16:0 containing | 776.7 | C48H90O6N | 0.000 | 0.000 | 0.051 | | | 0.070 |  |  | |
| 45:2 | 16:0 containing | 778.7 | C48H92O6N | 0.001 | 0.001 | 0.013 | | | 0.018 |  |  | |
| 45:1 | 16:0 containing | 780.7 | C48H94O6N | 0.016 | 0.014 | 0.015 | | | 0.015 |  |  | |
| 45:0 | 16:0 containing | 782.7 | C48H96O6N | 0.006 | 0.004 | 0.019 | | | 0.017 |  |  | |
| 46:2 | 16:0 containing | 792.7 | C49H94O6N | 0.026 | 0.009 | 0.031 | | | 0.032 |  |  | |
| 46:1 | 16:0 containing | 794.7 | C49H96O6N | 0.041 | 0.021 | 0.050 | | | 0.016 |  |  | |
| 46:0 | 16:0 containing | 796.7 | C49H98O6N | 0.077 | 0.031 | 0.097 | | | 0.027 |  |  | |
| 47:1 | 16:0 containing | 806.7 | C50H96O6N | 0.001 | 0.001 | 0.008 | | | 0.012 |  |  | |
| 47:0 | 16:0 containing | 808.7 | C50H98O6N | 0.011 | 0.005 | 0.012 | | | 0.011 |  |  | |
| K? | 16:0 containing | 810.8 | C50H100O6N | 0.021 | 0.020 | 0.035 | | | 0.014 |  |  | |
| 48:3 | 16:0 containing | 818.7 | C51H96O6N | 0.017 | 0.006 | 0.011 | | | 0.003 |  |  | |
| 48:2 | 16:0 containing | 820.7 | C51H98O6N | 0.094 | 0.031 | 0.079 | | | 0.013 |  |  | |
| 48:1 | 16:0 containing | 822.8 | C51H100O6N | 0.148 | 0.035 | 0.182 | | | 0.020 |  |  | |
| 48:0 | 16:0 containing | 824.8 | C51H102O6N | 0.120 | 0.041 | 0.183 | | | 0.024 | * |  | |
| O? | 16:0 containing | 832.7 | C52H98O6N | 0.003 | 0.003 | 0.002 | | | 0.003 |  |  | |
| 49:2 | 16:0 containing | 834.8 | C52H100O6N | 0.010 | 0.010 | 0.010 | | | 0.001 |  |  | |
| 49:1 | 16:0 containing | 836.8 | C52H102O6N | 0.010 | 0.010 | 0.025 | | | 0.007 |  |  | |
| P? | 16:0 containing | 838.8 | C52H104O6N | 0.009 | 0.009 | 0.016 | | | 0.008 |  |  | |
| 50:4 | 16:0 containing | 844.7 | C53H98O6N | 0.070 | 0.027 | 0.023 | | | 0.006 | * |  | |
| 50:3 | 16:0 containing | 846.8 | C53H100O6N | 0.332 | 0.086 | 0.175 | | | 0.043 | * |  | |
| 50:2 | 16:0 containing | 848.8 | C53H102O6N | 0.494 | 0.137 | 0.410 | | | 0.067 |  |  | |
| 50:1 | 16:0 containing | 850.8 | C53H104O6N | 0.391 | 0.114 | 0.509 | | | 0.078 |  |  | |
| 50:0 | 16:0 containing | 852.8 | C53H106O6N | 0.060 | 0.012 | 0.094 | | | 0.019 |  |  | |
| 864? | 16:0 containing | 864.7 |  | 0.057 | 0.021 | 0.027 | | | 0.013 |  |  | |
| 866? | 16:0 containing | 866.7 |  | 0.006 | 0.005 | 0.007 | | | 0.005 |  |  | |
| 52:6 | 16:0 containing | 868.7 | C55H98O6N | 0.027 | 0.009 | 0.006 | | | 0.004 | * |  | |
| 52:5 | 16:0 containing | 870.8 | C55H100O6N | 0.234 | 0.060 | 0.074 | | | 0.035 | * |  | |
| 52:4 | 16:0 containing | 872.8 | C55H102O6N | 0.810 | 0.189 | 0.329 | | | 0.090 | * |  | |
| 52:3 | 16:0 containing | 874.8 | C55H104O6N | 1.063 | 0.312 | 0.630 | | | 0.141 | * |  | |
| 52:2 | 16:0 containing | 876.8 | C55H106O6N | 0.620 | 0.188 | 0.490 | | | 0.072 |  |  | |
| 52:1 | 16:0 containing | 878.8 | C55H108O6N | 0.176 | 0.034 | 0.222 | | | 0.037 |  |  | |
| 886? | 16:0 containing | 886.8 |  | 0.004 | 0.002 | 0.009 | | | 0.014 |  |  | |
| 53:3 | 16:0 containing | 888.8 | C56H106O6N | 0.010 | 0.004 | 0.022 | | | 0.025 |  |  | |
| 53:2 | 16:0 containing | 890.8 | C56H108O6N | 0.010 | 0.004 | 0.021 | | | 0.025 |  |  | |
| 892? | 16:0 containing | 892.8 |  | 0.052 | 0.020 | 0.002 | | | 0.004 | * |  | |
| 900? | 16:0 containing | 900.8 |  | 0.001 | 0.001 | 0.004 | | | 0.007 |  |  | |
| 54:3 | 16:0 containing | 902.8 | C57H108O6N | 0.009 | 0.004 | 0.010 | | | 0.005 |  |  | |
| 54:2 | 16:0 containing | 904.8 | C57H110O6N | 0.013 | 0.007 | 0.015 | | | 0.010 |  |  | |
| 54:1 | 16:0 containing | 906.8 | C57H112O6N | 0.009 | 0.003 | 0.012 | | | 0.006 |  |  | |
| 54:0 | 16:0 containing | 908.9 | C57H114O6N | 0.009 | 0.004 | 0.011 | | | 0.004 |  |  | |
| 920? | 16:0 containing | 920.8 |  | 0.070 | 0.014 | 0.005 | | | 0.006 | * |  | |
| 56:1 | 16:0 containing | 934.9 | C59H116O6N | 0.017 | 0.002 | 0.014 | | | 0.008 |  |  | |
| 56:0 | 16:0 containing | 936.9 | C59H118O6N | 0.016 | 0.010 | 0.028 | | | 0.010 |  |  | |
| 948? | 16:0 containing | 948.8 |  | 0.089 | 0.012 | 0.011 | | | 0.011 | * |  | |
| 58:2 | 16:0 containing | 960.9 | C61H118O6N | 0.014 | 0.008 | 0.015 | | | 0.004 |  |  | |
| 58:1 | 16:0 containing | 962.9 | C61H120O6N | 0.025 | 0.003 | 0.029 | | | 0.011 |  |  | |
| 58:0 | 16:0 containing | 964.9 | C61H122O6N | 0.004 | 0.002 | 0.013 | | | 0.012 |  |  | |
| 976? | 16:0 containing | 976.9 |  | 0.048 | 0.013 | 0.013 | | | 0.011 | * |  | |
| 60:2 | 16:0 containing | 988.9 | C63H122O6N | 0.001 | 0.001 | 0.001 | | | 0.000 |  |  | |
| 988? | 16:0 containing | 988.9 |  | 0.001 | 0.001 | 0.001 | | | 0.000 |  |  | |
| 60:1 | 16:0 containing | 990.9 | C63H124O6N | 0.001 | 0.001 | 0.003 | | | 0.001 | * |  | |
| **Total 16:0 TAG** | |  |  | **5.398** | **1.498** | **4.186** | | | **0.534** |  |  | |
| 40:4 | 18:3 containing | 704.6 | C43H78O6N | 0.116 | 0.002 | 0.074 | | | 0.047 |  |  | |
| 40:3 | 18:3 containing | 706.6 | C43H80O6N | 0.000 | 0.000 | 0.001 | | | 0.002 |  |  | |
| L? | 18:3 containing | 812.8 | C50H102O6N | 0.002 | 0.002 | 0.000 | | | 0.000 |  |  | |
| M? | 18:3 containing | 816.7 | C51H94O6N | 0.005 | 0.003 | 0.001 | | | 0.000 | * |  | |
| 50:4 | 18:3 containing | 844.7 | C53H98O6N | 0.031 | 0.009 | 0.010 | | | 0.006 | * |  | |
| 50:3 | 18:3 containing | 846.8 | C53H100O6N | 0.006 | 0.005 | 0.002 | | | 0.002 |  |  | |
| 866? | 18:3 containing | 866.7 |  | 0.023 | 0.004 | 0.008 | | | 0.005 | * |  | |
| 52:6 | 18:3 containing | 868.7 | C55H98O6N | 0.088 | 0.014 | 0.059 | | | 0.025 |  |  | |
| 52:5 | 18:3 containing | 870.8 | C55H100O6N | 0.129 | 0.025 | 0.078 | | | 0.016 | * |  | |
| 52:4 | 18:3 containing | 872.8 | C55H102O6N | 0.091 | 0.025 | 0.059 | | | 0.011 |  |  | |
| 886? | 18:3 containing | 886.8 |  | 0.002 | 0.001 | 0.011 | | | 0.019 |  |  | |
| 53:2 | 18:3 containing | 890.8 | C56H108O6N | 0.018 | 0.001 | 0.005 | | | 0.003 | * |  | |
| 892? | 18:3 containing | 892.8 |  | 0.085 | 0.017 | 0.020 | | | 0.010 | * |  | |
| 898? | 18:3 containing | 898.8 |  | 0.150 | 0.030 | 0.069 | | | 0.019 | * |  | |
| 900? | 18:3 containing | 900.8 |  | 0.044 | 0.016 | 0.019 | | | 0.003 | * |  | |
| 54:0 | 18:3 containing | 908.9 | C57H114O6N | 0.001 | 0.002 | 0.005 | | | 0.007 |  |  | |
| 914? | 18:3 containing | 914.8 |  | 0.000 | 0.000 | 0.001 | | | 0.002 |  |  | |
| **Total 18:3 TAG** | |  |  | **0.791** | **0.137** | **0.423** | | | **0.064** | * |  | |
| 40:3 | 18:2 containing | 706.6 | C43H80O6N | 0.115 | 0.011 | 0.103 | | | 0.051 |  |  | |
| 40:2 | 18:2 containing | 708.6 | C43H82O6N | 0.002 | 0.001 | 0.000 | | | 0.000 | * |  | |
| 41:3 | 18:2 containing | 720.6 | C44H82O6N | 0.013 | 0.002 | 0.026 | | | 0.011 |  |  | |
| 41:2 | 18:2 containing | 722.6 | C44H84O6N | 0.009 | 0.001 | 0.009 | | | 0.002 |  |  | |
| 41:1 | 18:2 containing | 724.6 | C44H86O6N | 0.001 | 0.001 | 0.004 | | | 0.004 |  |  | |
| D? | 18:2 containing | 748.6 | C46H86O6N | 0.000 | 0.000 | 0.000 | | | 0.000 |  |  | |
| H? | 18:2 containing | 788.7 | C49H90O6N | 0.001 | 0.001 | 0.007 | | | 0.004 | * |  | |
| 46:3 | 18:2 containing | 790.7 | C49H92O6N | 0.007 | 0.004 | 0.010 | | | 0.005 |  |  | |
| 46:2 | 18:2 containing | 792.7 | C49H94O6N | 0.023 | 0.001 | 0.013 | | | 0.005 | * |  | |
| M? | 18:2 containing | 816.7 | C51H94O6N | 0.017 | 0.006 | 0.012 | | | 0.003 |  |  | |
| 48:3 | 18:2 containing | 818.7 | C51H96O6N | 0.046 | 0.005 | 0.025 | | | 0.009 | * |  | |
| 48:2 | 18:2 containing | 820.7 | C51H98O6N | 0.052 | 0.010 | 0.039 | | | 0.005 |  |  | |
| O? | 18:2 containing | 832.7 | C52H98O6N | 0.002 | 0.001 | 0.003 | | | 0.003 |  |  | |
| 49:2 | 18:2 containing | 834.8 | C52H100O6N | 0.003 | 0.003 | 0.003 | | | 0.002 |  |  | |
| 50:4 | 18:2 containing | 844.7 | C53H98O6N | 0.117 | 0.023 | 0.075 | | | 0.028 |  |  | |
| 50:3 | 18:2 containing | 846.8 | C53H100O6N | 0.223 | 0.056 | 0.151 | | | 0.036 |  |  | |
| 50:2 | 18:2 containing | 848.8 | C53H102O6N | 0.077 | 0.024 | 0.090 | | | 0.024 |  |  | |
| 866? | 18:2 containing | 866.7 |  | 0.008 | 0.001 | 0.001 | | | 0.001 | * |  | |
| 52:6 | 18:2 containing | 868.7 | C55H98O6N | 0.116 | 0.025 | 0.041 | | | 0.023 | * |  | |
| 52:5 | 18:2 containing | 870.8 | C55H100O6N | 0.403 | 0.114 | 0.188 | | | 0.089 | * |  | |
| 52:4 | 18:2 containing | 872.8 | C55H102O6N | 0.714 | 0.182 | 0.454 | | | 0.179 |  |  | |
| 52:3 | 18:2 containing | 874.8 | C55H104O6N | 0.641 | 0.153 | 0.480 | | | 0.124 |  |  | |
| 52:2 | 18:2 containing | 876.8 | C55H106O6N | 0.059 | 0.023 | 0.051 | | | 0.022 |  |  | |
| 886? | 18:2 containing | 886.8 |  | 0.033 | 0.013 | 0.023 | | | 0.017 |  |  | |
| 53:3 | 18:2 containing | 888.8 | C56H106O6N | 0.016 | 0.004 | 0.009 | | | 0.002 | * |  | |
| 53:2 | 18:2 containing | 890.8 | C56H108O6N | 0.004 | 0.002 | 0.001 | | | 0.000 | * |  | |
| 892? | 18:2 containing | 892.8 |  | 0.067 | 0.024 | 0.015 | | | 0.012 | * |  | |
| 898? | 18:2 containing | 898.8 |  | 0.784 | 0.160 | 0.458 | | | 0.201 | * |  | |
| 900? | 18:2 containing | 900.8 |  | 0.631 | 0.145 | 0.400 | | | 0.127 |  |  | |
| 54:3 | 18:2 containing | 902.8 | C57H108O6N | 0.248 | 0.056 | 0.151 | | | 0.044 | * |  | |
| 54:2 | 18:2 containing | 904.8 | C57H110O6N | 0.005 | 0.003 | 0.013 | | | 0.005 | * |  | |
| 54:0 | 18:2 containing | 908.9 | C57H114O6N | 0.002 | 0.001 | 0.001 | | | 0.001 | * |  | |
| 914? | 18:2 containing | 914.8 |  | 0.008 | 0.007 | 0.011 | | | 0.018 |  |  | |
| 916? | 18:2 containing | 916.8 |  | 0.003 | 0.002 | 0.004 | | | 0.006 |  |  | |
| 922? | 18:2 containing | 922.8 |  | 0.003 | 0.002 | 0.002 | | | 0.004 |  |  | |
| 928? | 18:2 containing | 928.8 |  | 0.007 | 0.005 | 0.008 | | | 0.002 |  |  | |
| 930? | 18:2 containing | 930.8 |  | 0.003 | 0.002 | 0.003 | | | 0.001 |  |  | |
| 56:2 | 18:2 containing | 932.9 | C59H114O6N | 0.005 | 0.002 | 0.005 | | | 0.005 |  |  | |
| 958? | 18:2 containing | 958.8 |  | 0.005 | 0.002 | 0.004 | | | 0.002 |  |  | |
| 58:2 | 18:2 containing | 960.9 | C61H118O6N | 0.008 | 0.008 | 0.017 | | | 0.001 |  |  | |
| 60:2 | 18:2 containing | 988.9 | C63H122O6N | 0.003 | 0.002 | 0.005 | | | 0.002 |  |  | |
| 988? | 18:2 containing | 988.9 |  | 0.003 | 0.002 | 0.005 | | | 0.002 |  |  | |
| **Total 18:2 TAG** | |  |  | **4.489** | **1.044** | **2.918** | | | **0.927** |  |  | |
| 40:2 | 18:1 containing | 708.6 | C43H82O6N | 0.105 | 0.004 | 0.094 | | | 0.026 |  |  | |
| 40:1 | 18:1 containing | 710.6 | C43H84O6N | 0.001 | 0.003 | 0.001 | | | 0.001 |  |  | |
| 41:2 | 18:1 containing | 722.6 | C44H84O6N | 0.007 | 0.004 | 0.018 | | | 0.005 | * |  | |
| 41:1 | 18:1 containing | 724.6 | C44H86O6N | 0.012 | 0.001 | 0.010 | | | 0.007 |  |  | |
| 41:0 | 18:1 containing | 726.7 | C44H88O6N | 0.000 | 0.000 | 0.003 | | | 0.003 |  |  | |
| 42:2 | 18:1 containing | 736.6 | C45H86O6N | 0.000 | 0.000 | 0.001 | | | 0.002 |  |  | |
| C? | 18:1 containing | 742.7 | C45H92O6N | 0.000 | 0.001 | 0.000 | | | 0.000 |  |  | |
| 43:2 | 18:1 containing | 750.7 | C46H88O6N | 0.002 | 0.003 | 0.000 | | | 0.000 |  |  | |
| 44:2 | 18:1 containing | 764.7 | C47H90O6N | 0.001 | 0.001 | 0.003 | | | 0.003 |  |  | |
| 44:1 | 18:1 containing | 766.7 | C47H92O6N | 0.003 | 0.003 | 0.005 | | | 0.004 |  |  | |
| 46:3 | 18:1 containing | 790.7 | C49H92O6N | 0.002 | 0.001 | 0.008 | | | 0.006 |  |  | |
| 46:2 | 18:1 containing | 792.7 | C49H94O6N | 0.003 | 0.002 | 0.016 | | | 0.011 |  |  | |
| 46:1 | 18:1 containing | 794.7 | C49H96O6N | 0.021 | 0.004 | 0.021 | | | 0.004 |  |  | |
| 47:1 | 18:1 containing | 806.7 | C50H96O6N | 0.001 | 0.002 | 0.007 | | | 0.007 |  |  | |
| 47:0 | 18:1 containing | 808.7 | C50H98O6N | 0.004 | 0.004 | 0.013 | | | 0.012 |  |  | |
| 48:3 | 18:1 containing | 818.7 | C51H96O6N | 0.013 | 0.001 | 0.015 | | | 0.004 |  |  | |
| 48:2 | 18:1 containing | 820.7 | C51H98O6N | 0.049 | 0.014 | 0.046 | | | 0.010 |  |  | |
| 48:1 | 18:1 containing | 822.8 | C51H100O6N | 0.177 | 0.069 | 0.169 | | | 0.023 |  |  | |
| O? | 18:1 containing | 832.7 | C52H98O6N | 0.001 | 0.002 | 0.001 | | | 0.001 |  |  | |
| 49:2 | 18:1 containing | 834.8 | C52H100O6N | 0.007 | 0.004 | 0.010 | | | 0.005 |  |  | |
| 49:1 | 18:1 containing | 836.8 | C52H102O6N | 0.005 | 0.005 | 0.010 | | | 0.006 |  |  | |
| 50:4 | 18:1 containing | 844.7 | C53H98O6N | 0.028 | 0.007 | 0.015 | | | 0.007 | * |  | |
| 50:3 | 18:1 containing | 846.8 | C53H100O6N | 0.133 | 0.030 | 0.109 | | | 0.027 |  |  | |
| 50:2 | 18:1 containing | 848.8 | C53H102O6N | 0.237 | 0.060 | 0.254 | | | 0.028 |  |  | |
| 50:1 | 18:1 containing | 850.8 | C53H104O6N | 0.087 | 0.030 | 0.131 | | | 0.001 | * |  | |
| 864? | 18:1 containing | 864.7 |  | 0.005 | 0.003 | 0.010 | | | 0.004 |  |  | |
| 52:6 | 18:1 containing | 868.7 | C55H98O6N | 0.008 | 0.001 | 0.001 | | | 0.001 | * |  | |
| 52:5 | 18:1 containing | 870.8 | C55H100O6N | 0.108 | 0.012 | 0.056 | | | 0.038 |  |  | |
| 52:4 | 18:1 containing | 872.8 | C55H102O6N | 0.454 | 0.110 | 0.280 | | | 0.100 |  |  | |
| 52:3 | 18:1 containing | 874.8 | C55H104O6N | 0.842 | 0.189 | 0.647 | | | 0.156 |  |  | |
| 52:2 | 18:1 containing | 876.8 | C55H106O6N | 0.729 | 0.182 | 0.708 | | | 0.079 |  |  | |
| 52:1 | 18:1 containing | 878.8 | C55H108O6N | 0.055 | 0.009 | 0.093 | | | 0.013 | * |  | |
| 886? | 18:1 containing | 886.8 |  | 0.025 | 0.005 | 0.015 | | | 0.007 |  |  | |
| 53:3 | 18:1 containing | 888.8 | C56H106O6N | 0.029 | 0.013 | 0.025 | | | 0.009 |  |  | |
| 53:2 | 18:1 containing | 890.8 | C56H108O6N | 0.023 | 0.010 | 0.016 | | | 0.009 |  |  | |
| 892? | 18:1 containing | 892.8 |  | 0.004 | 0.003 | 0.003 | | | 0.002 |  |  | |
| 898? | 18:1 containing | 898.8 |  | 0.813 | 0.113 | 0.448 | | | 0.203 | * |  | |
| 900? | 18:1 containing | 900.8 |  | 1.031 | 0.268 | 0.693 | | | 0.210 |  |  | |
| 54:3 | 18:1 containing | 902.8 | C57H108O6N | 0.731 | 0.182 | 0.575 | | | 0.085 |  |  | |
| 54:2 | 18:1 containing | 904.8 | C57H110O6N | 0.258 | 0.056 | 0.201 | | | 0.012 |  |  | |
| 54:1 | 18:1 containing | 906.8 | C57H112O6N | 0.015 | 0.002 | 0.026 | | | 0.005 | * |  | |
| 914? | 18:1 containing | 914.8 |  | 0.020 | 0.009 | 0.037 | | | 0.036 |  |  | |
| 916? | 18:1 containing | 916.8 |  | 0.014 | 0.008 | 0.028 | | | 0.020 |  |  | |
| 918? | 18:1 containing | 918.8 |  | 0.013 | 0.004 | 0.012 | | | 0.012 |  |  | |
| 920? | 18:1 containing | 920.8 |  | 0.001 | 0.002 | 0.002 | | | 0.000 |  |  | |
| 928? | 18:1 containing | 928.8 |  | 0.015 | 0.005 | 0.012 | | | 0.003 |  |  | |
| 930? | 18:1 containing | 930.8 |  | 0.016 | 0.006 | 0.024 | | | 0.013 |  |  | |
| 56:2 | 18:1 containing | 932.9 | C59H114O6N | 0.010 | 0.003 | 0.011 | | | 0.003 |  |  | |
| 56:1 | 18:1 containing | 934.9 | C59H116O6N | 0.014 | 0.004 | 0.018 | | | 0.003 |  |  | |
| 946? | 18:1 containing | 946.8 |  | 0.010 | 0.008 | 0.003 | | | 0.004 |  |  | |
| 958? | 18:1 containing | 958.8 |  | 0.001 | 0.001 | 0.004 | | | 0.004 |  |  | |
| 58:2 | 18:1 containing | 960.9 | C61H118O6N | 0.011 | 0.006 | 0.015 | | | 0.004 |  |  | |
| 58:1 | 18:1 containing | 962.9 | C61H120O6N | 0.017 | 0.001 | 0.027 | | | 0.007 | * |  | |
| 976? | 18:1 containing | 976.9 |  | 0.001 | 0.001 | 0.000 | | | 0.000 |  |  | |
| 60:2 | 18:1 containing | 988.9 | C63H122O6N | 0.023 | 0.004 | 0.024 | | | 0.005 |  |  | |
| 988? | 18:1 containing | 988.9 |  | 0.023 | 0.004 | 0.024 | | | 0.005 |  |  | |
| 60:1 | 18:1 containing | 990.9 | C63H124O6N | 0.009 | 0.002 | 0.014 | | | 0.008 |  |  | |
| **Total 18:1 TAG** | |  |  | **6.228** | **1.388** | **5.012** | | | **0.804** |  |  | |
| 40:1 | 18:0 containing | 710.6 | C43H84O6N | 0.021 | 0.002 | 0.027 | | | 0.007 |  |  | |
| 40:0 | 18:0 containing | 712.6 | C43H86O6N | 0.000 | 0.000 | 0.000 | | | 0.000 |  |  | |
| 46:1 | 18:0 containing | 794.7 | C49H96O6N | 0.001 | 0.001 | 0.005 | | | 0.004 |  |  | |
| 46:0 | 18:0 containing | 796.7 | C49H98O6N | 0.007 | 0.005 | 0.010 | | | 0.001 |  |  | |
| 48:2 | 18:0 containing | 820.7 | C51H98O6N | 0.005 | 0.003 | 0.003 | | | 0.002 |  |  | |
| 48:1 | 18:0 containing | 822.8 | C51H100O6N | 0.011 | 0.001 | 0.019 | | | 0.004 | * |  | |
| 48:0 | 18:0 containing | 824.8 | C51H102O6N | 0.058 | 0.018 | 0.073 | | | 0.008 |  |  | |
| 49:1 | 18:0 containing | 836.8 | C52H102O6N | 0.001 | 0.001 | 0.001 | | | 0.001 |  |  | |
| P? | 18:0 containing | 838.8 | C52H104O6N | 0.002 | 0.001 | 0.005 | | | 0.004 |  |  | |
| 50:3 | 18:0 containing | 846.8 | C53H100O6N | 0.006 | 0.004 | 0.002 | | | 0.001 |  |  | |
| 50:2 | 18:0 containing | 848.8 | C53H102O6N | 0.038 | 0.016 | 0.037 | | | 0.011 |  |  | |
| 50:1 | 18:0 containing | 850.8 | C53H104O6N | 0.095 | 0.025 | 0.134 | | | 0.038 |  |  | |
| 50:0 | 18:0 containing | 852.8 | C53H106O6N | 0.040 | 0.011 | 0.053 | | | 0.010 |  |  | |
| 864? | 18:0 containing | 864.7 |  | 0.018 | 0.007 | 0.030 | | | 0.002 | * |  | |
| 866? | 18:0 containing | 866.7 |  | 0.004 | 0.004 | 0.004 | | | 0.004 |  |  | |
| 52:4 | 18:0 containing | 872.8 | C55H102O6N | 0.030 | 0.002 | 0.010 | | | 0.009 | * |  | |
| 52:3 | 18:0 containing | 874.8 | C55H104O6N | 0.151 | 0.045 | 0.098 | | | 0.035 |  |  | |
| 52:2 | 18:0 containing | 876.8 | C55H106O6N | 0.279 | 0.045 | 0.278 | | | 0.018 |  |  | |
| 52:1 | 18:0 containing | 878.8 | C55H108O6N | 0.265 | 0.057 | 0.324 | | | 0.058 |  |  | |
| 886? | 18:0 containing | 886.7 |  | 0.000 | 0.000 | 0.000 | | | 0.000 |  |  | |
| 53:3 | 18:0 containing | 888.8 | C56H106O6N | 0.004 | 0.002 | 0.002 | | | 0.000 |  |  | |
| 53:2 | 18:0 containing | 890.8 | C56H108O6N | 0.009 | 0.008 | 0.010 | | | 0.004 |  |  | |
| 892? | 18:0 containing | 892.8 |  | 0.012 | 0.009 | 0.016 | | | 0.013 |  |  | |
| 898? | 18:0 containing | 898.8 |  | 0.112 | 0.029 | 0.038 | | | 0.022 | * |  | |
| 900? | 18:0 containing | 900.8 |  | 0.293 | 0.071 | 0.164 | | | 0.083 |  |  | |
| 54:3 | 18:0 containing | 902.8 | C57H108O6N | 0.405 | 0.104 | 0.249 | | | 0.067 | * |  | |
| 54:2 | 18:0 containing | 904.8 | C57H110O6N | 0.269 | 0.067 | 0.242 | | | 0.009 |  |  | |
| 54:1 | 18:0 containing | 906.8 | C57H112O6N | 0.139 | 0.019 | 0.144 | | | 0.002 |  |  | |
| 54:0 | 18:0 containing | 908.9 | C57H114O6N | 0.009 | 0.003 | 0.015 | | | 0.009 |  |  | |
| 914? | 18:0 containing | 914.8 |  | 0.002 | 0.002 | 0.003 | | | 0.004 |  |  | |
| 916? | 18:0 containing | 916.8 |  | 0.003 | 0.001 | 0.009 | | | 0.010 |  |  | |
| 918? | 18:0 containing | 918.8 |  | 0.003 | 0.003 | 0.008 | | | 0.010 |  |  | |
| 920? | 18:0 containing | 920.8 |  | 0.008 | 0.003 | 0.005 | | | 0.004 |  |  | |
| 930? | 18:0 containing | 930.8 |  | 0.001 | 0.001 | 0.004 | | | 0.006 |  |  | |
| 56:2 | 18:0  containing | 932.9 | C59H114O6N | 0.005 | 0.003 | 0.003 | | | 0.001 |  |  | |
| 56:1 | 18:0 containing | 934.9 | C59H116O6N | 0.006 | 0.003 | 0.004 | | | 0.003 |  |  | |
| 56:0 | 18:0 containing | 936.9 | C59H118O6N | 0.004 | 0.002 | 0.005 | | | 0.003 |  |  | |
| 58:1 | 18:0 containing | 962.9 | C61H120O6N | 0.007 | 0.002 | 0.013 | | | 0.000 | * |  | |
| 58:0 | 18:0 containing | 964.9 | C61H122O6N | 0.008 | 0.007 | 0.020 | | | 0.001 | * |  | |
| 976? | 18:0 containing | 976.9 |  | 0.006 | 0.004 | 0.000 | | | 0.000 | * |  | |
| 60:2 | 18:0 containing | 988.9 | C63H122O6N | 0.007 | 0.003 | 0.008 | | | 0.005 |  |  | |
| 988? | 18:0 containing | 988.9 |  | 0.007 | 0.003 | 0.008 | | | 0.005 |  |  | |
| 60:1 | 18:0 containing | 990.9 | C63H124O6N | 0.017 | 0.006 | 0.022 | | | 0.003 |  |  | |
| 60:0 | 18:0 containing | 993.0 | C63H126O6N | 0.002 | 0.002 | 0.004 | | | 0.001 | * |  | |
| **Total 18:0 TAG** | |  |  | **2.367** | **0.566** | **2.110** | | | **0.306** |  |  | |
