# Supplementary material for: Calcineurin Signaling and Membrane Lipid Homeostasis Regulates Iron Mediated MultiDrug Resistance Mechanisms in Candida albicans
Source: PLoS One. 2011 Apr 12;6(4):e18684. doi: 10.1371/journal.pone.0018684 (PMC3075269; doi:10.1371/journal.pone.0018684)
Supplement: Table S8 — List of C. albicans strains used in the study. (DOC) [file pone.0018684.s010.doc]

**Table: S8**

| **Strains** | **Relevant Genotype** | **Reference** |
| --- | --- | --- |
| **CAF2-1** | *ura3Δ::imm434/URA3 iro1Δ::imm434/IRO1* | [21] |
| ***∆ftr1*** | Δ*ura3*::*imm434/*Δ*ura3*::*imm434*  Δ*ftr1*::*hisG/*Δ*ftr1*::*hisG* | [4] |
| ***∆ccc2*** | Δ*ura3*::*imm434/*Δ*ura3*::*imm434*  Δ*ccc2*::*hisG/*Δ*ccc2*::*hisG* | [22] |
| **CMP1M3A** | *cmp1*::*URA3-FLIP/cmp1*::*FRT* | [18] |
| **JRB64** | *ura3Δ::λimm434/ura3Δ::λimm434 his1::hisG::HIS1/his1::hisG arg4::hisG/arg4::hisG*  *cnb1::UAU/cnb1::ARG4* | [37] |
| **DSY2146** | cnaΔ::hisG/cnaΔ::hisG/ LEU2::CNAtr::URA3 | [23] |
| **DSY2195** | *crz1Δ::hisG/crz1Δ::hisG::URA3::hisG* | [27] |
| **SN95** | *arg4*Δ*/arg4*Δ *his1*Δ*/his1*Δ *URA3/ura3*Δ::*imm434 IRO1/iro1*Δ::*imm434* | [25] |
| **CaEE73** | As SN95; *age3::HIS1/age3::ARG4* | [24] |
| **CaLC436** | As SN95; *HIS1/his1::TAR-FRT hsp90::CdHIS1/tetO-HSP90* | [26] |
| **CaLC700** | As SN95; *mkc1::FRT/mkc1::FRT* | [28] |
| **CaLC896** | As SN95; *bck1::FRT/bck1::FRT* | [28] |
| **CaLC1255** | As SN95; *pkc1::FRT/pkc1::FRT CaTAR::HIS3* | [28] |
