# Supplementary material for: Calcineurin Signaling and Membrane Lipid Homeostasis Regulates Iron Mediated MultiDrug Resistance Mechanisms in Candida albicans
Source: PLoS One. 2011 Apr 12;6(4):e18684. doi: 10.1371/journal.pone.0018684 (PMC3075269; doi:10.1371/journal.pone.0018684)
Supplement: Table S9 — List of primer sequences for RT-PCR used in the study. (DOC) [file pone.0018684.s011.doc]

**Table: S9**

| **Gene** | **Oligonucleotide Sequence** |
| --- | --- |
| **ERG1** | ERG1 FP 5’ GCTATTATTATTGGTGCAGGG 3’  ERG1 RP 5’ GACACTGAATATATCAATGCTG 3’ |
| **ERG2** | ERG2 FP 5’ GAAGTTATTATTAGTAGG 3’  ERG2 RP 5’ CCAAAAATCCAAATGGTAAC 3’ |
| **ERG3** | ERG3 FP 5’ GGATATCGTACTAGAAATTTGTG 3’  ERG3 RP 5’ CGGTGAAAAGAATGAATTTAGG 3’ |
| **ERG11** | ERG11 FP 5’ GCTATTGTTGAAACTGTCATTG 3’  ERG11 RP 5’ GAAGCAGAAGTATGTTGACCA 3’ |
| **ERG25** | ERG25 FP 5’ CAATGATTTATTTGCTACTGG 3’  ERG25 RP 5’ GACTTTGTCTTCTCTACCC 3’ |
| **AUR1** | AUR1 FP 5’ CCCGTTTTCACTTGGTTGGC 3’  AUR1 RP 5’ GTGCTACCCTGTGGTTCGTC 3’ |
| **SCS7** | SCS7 FP 5’ GAAGAACATCCTGGTGGTGG 3’  SCS7 RP 5’ CGATGTAAACAGTATTCCAC 3’ |
| **HSP90** | HSP90 FP 5’ CTACTCCTTGTTCTTGGTTGC 3’  HSP90 RP 5’ CTCTTCAGCATCATCAGTG 3’ |
| **CMP1** | CMP1 FP 5’ CATTTTGGATGCTTAGAGG 3’  CMP1 RP 5’GAGGTAACCAATAAGGATG 3’ |
| **CRZ1** | CRZ1 FP 5’ CACAGGAGAGATACAACTTGG 3’  CRZ1 RP 5’ CTAACATTTTCTCTCGTGATTG 3’ |
| **RIM8** | RIM8 FP 5’ GAACAAACAGACTCAGCAGAG 3’  RIM8 RP 5’ GTGCTTGCTCTTCTTGCGTTG 3’ |
| **RHR2** | RHR2 FP 5’ CGTTCACGCCGCTTTATTTG 3’  RHR2 RP 5’ GTCAGTTTCCTTGTTGTATGAG 3’ |
