# Supplementary material for: Calcineurin Signaling and Membrane Lipid Homeostasis Regulates Iron Mediated MultiDrug Resistance Mechanisms in Candida albicans
Source: PLoS One. 2011 Apr 12;6(4):e18684. doi: 10.1371/journal.pone.0018684 (PMC3075269; doi:10.1371/journal.pone.0018684)
Supplement: Table S10 — Scans used to detect the lipid species containing a common head group fragment. (DOC) [file pone.0018684.s012.doc]

**Table: S10**

| **Name** | **Positive/**  **Negative ion mode** | **Sequential precursor (Pre) and neutral loss (NL) scans** |
| --- | --- | --- |
| **PGL** | | |
| PC and LysoPC | [M + H]^+^ | +Pre 184.1 |
| PE and LysoPE | [M + H]^+^ | +NL 141.0 |
| PA | [M + NH_4_]^+^ | +NL 115.0 |
| PG | [M + NH_4_]^+^ | +NL 189.0 |
| PI | [M + NH_4_]^+^ | +NL 277.0 |
| PS | [M + H]^+^ | +NL 185.0 |
| LysoPG | [M – H]^-^ | +Pre 152.9 |
| **SL** | | |
| CER | [M + H – H_2_O]^+^ | +Pre 300.0 |
| IPC | [M – H]^-^ | -Pre 259.0 |
| MIPC | [M – H]^-^ | -Pre 421.0 |
| M(IP)_2_C | [M – H]^-^ | -Pre 663.1 |
| **SE, DAG and TAG** | | |
| 16:1 | [M + NH_4_]^+^ | +NL 271.2 |
| 16:0 | [M + NH_4_]^+^ | +NL 273.2 |
| 18:3 | [M + NH_4_]^+^ | +NL 295.2 |
| 18:2 | [M + NH_4_]^+^ | +NL 297.2 |
| 18:1 | [M + NH_4_]^+^ | +NL 299.2 |
| 18:0 | [M + NH_4_]^+^ | +NL 301.2 |
