# Supplementary material for: Calcineurin Signaling and Membrane Lipid Homeostasis Regulates Iron Mediated MultiDrug Resistance Mechanisms in Candida albicans
Source: PLoS One. 2011 Apr 12;6(4):e18684. doi: 10.1371/journal.pone.0018684 (PMC3075269; doi:10.1371/journal.pone.0018684)
Supplement: Table S11 — Amount of internal standard used for the lipid quantification. (DOC) [file pone.0018684.s013.doc]

**Table: S11**

| **Name** | **Amount (nmol)** |
| --- | --- |
| di12:0-PC | 0.60 |
| di24:1-PC | 0.60 |
| 13:0-LysoPC | 0.60 |
| 19:0-LysoPC | 0.60 |
| di12:0-PE | 0.30 |
| di23:0-PE | 0.30 |
| 14:0-LysoPE | 0.30 |
| 18:0-LysoPE | 0.30 |
| di14:0-PG | 0.30 |
| di20:0(phytanoyl)-PG | 0.30 |
| 14:0-LysoPG | 0.30 |
| 18:0-LysoPG | 0.30 |
| di14:0-PA | 0.30 |
| di20:0(phytanoyl)-PA | 0.30 |
| di14:0-PS | 0.20 |
| di20:0(phytanoyl)-PS | 0.20 |
| 16:0-18:0-PI | 0.23 |
| di18:0-PI | 0.16 |
| di15:0-DAG | 4.60 |
| tri17:0-TAG | 3.10 |
